# 1-Deazainosine-impact on RNA structure and role in exploring ribozyme catalysis

**DOI:** 10.1039/d6sc04009h

**Published:** 2026-06-08

**Authors:** Christoph Mitteregger, Raphael Bereiter, Antoine Schramm, Eric Ennifar, Christoph Kreutz, Ronald Micura

**Affiliations:** a Institute of Organic Chemistry, Center for Molecular Biosciences, Innsbruck (CMBI), University of Innsbruck Innrain 80-82 Innsbruck 6020 Austria ronald.micurauibk.ac.at; b Architecture et Réactivité de l’ARN-CNRS UPR 9002, Institut de BiologieMoléculaire et Cellulaire, Université de Strasbourg Strasbourg 67000 France

## Abstract

Synthetic RNAs bearing deazapurine nucleobases are powerful probes for dissecting RNA-catalyzed reactions by atomic mutagenesis. Here we systematically characterize RNA containing 1-deazainosine (c^1^I) and compare it with inosine (I). We first report the synthesis of a suitably protected c^1^I phosphoramidite and its incorporation into RNA by solid-phase synthesis. We then provide a comprehensive thermodynamic analysis of base-pair stability from UV-melting experiments, showing that c^1^I–C pairs are less stable than the corresponding I–C pairs. Although a two-hydrogen-bond Hoogsteen interaction between c^1^I and protonated C is conceivable, NMR spectroscopy indicates that c^1^I–C predominantly adopts a Watson–Crick-like geometry with a single hydrogen bond. These pairs are accommodated within RNA duplexes without disrupting neighboring base pairing. We also use c^1^I to probe poly(I:C) motifs that mimic viral double-stranded RNA, assessing how strand length governs duplex *versus* hairpin formation. Finally, atomic mutagenesis of the twister ribozyme with c^1^I supports the hypothesis that an active-site guanine participates directly in phosphodiester-bond cleavage. Together, these results clarify how deazapurines modulate nucleic-acid properties and provide guidance for their use in atomic mutagenesis to interrogate RNA catalysis.

## Abstract 2

Synthetic RNAs bearing deazapurine nucleobases are powerful probes for dissecting RNA-catalyzed reactions by atomic mutagenesis.

## Graphical abstract

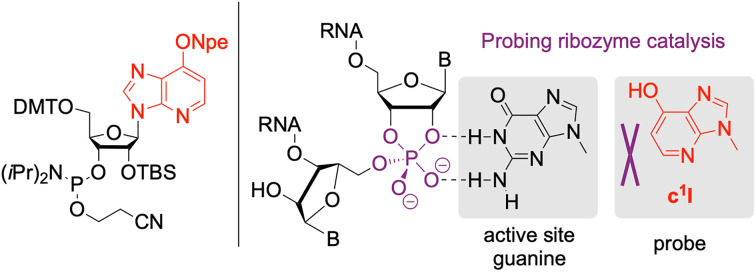

## Introduction

**Fig. 1:**
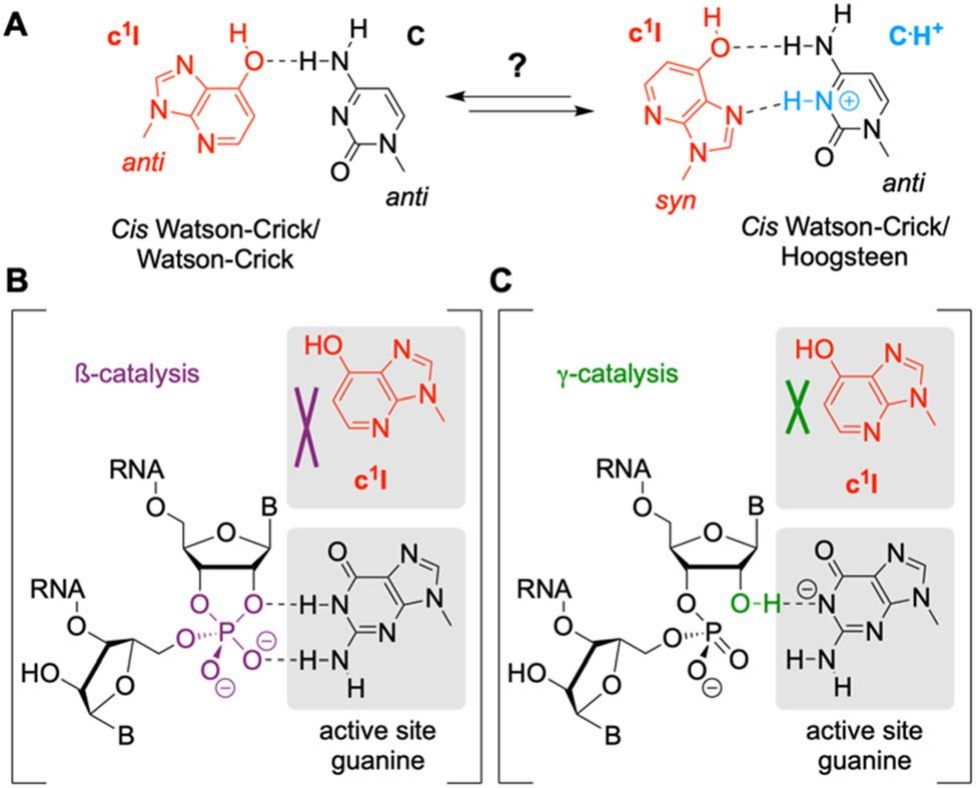
(A) Possible pairing patterns for 1-deazainosine with cytidine (c^1^I–C) in RNA: Watson–Crick *vs.* Hoogsteen. (B) The cleavage reaction of nucleolytic ribozymes can involve a pentavalent phosphorane transition state that is stabilized by interactions with an active-site guanine (β-catalysis). Access to c^1^I allows evaluation of these interactions in functional assays. (C) Same as B but for deprotonation of the attacking 2′-OH (γ-catalysis).

Deazanucleoside modifications are useful for atomic mutagenesis studies to explore RNA structure, function, and reactivity.^1–5^ Thereby, the replacement of a nitrogen atom in a nucleobase by carbon influences the properties of RNA because the hydrogen acceptor capability of an imino functionality (=N–) or the hydrogen donor capability of an amido or amino functionality (–NH–) becomes impaired at the specific position.^6–8^ This is crucial for base pairing,^8,9^ RNA-protein recognition,^3,6,8,9^ RNA-small molecule recognition,^10^ and RNA catalysis.^4,11–14^ Atomic mutagenesis has contributed to our mechanistic understanding of ribozymes,^15–18^ including the ribosome.^19–22^ So far, the selection of deazanucleosides that is available for RNA atomic mutagenesis experiments include 3-deazacytidine (c^3^C),^14,23,24^ 7-deazaadenosine (c^7^A),^4,12–14,16,17^ 3-deazaadenosine (c^3^A),^15,23^ 1-deazaadenosine (c^1^A),^12,13,15,23^ 1,3-dideazaadenosine,^14^ 7-deazaguanosine (c^7^G),^14,16^ 3-deazaguanosine (c^3^G),^25,26^ 1-deazaguanosine (c^1^G),^27^ and 1,3-dideazaguanosine.^28^ Another modification that usefully adds to the deazanucleoside toolbox is 1-deazainosine (c^1^I). This modification is particularly powerful to probe the involvement of guanine in transition state stabilization of self-cleaving ribozymes (see Fig. 1). However, there is only one report in the literature describing the application of a c^1^I-modified variant of the pistol ribozyme.^29^ Information on the chemical synthesis and biophysical properties of c^1^I RNA has been lacking so far.

In the present work, we report a robust synthesis of an appropriately protected c^1^I phosphoramidite building block and its incorporation into oligoribonucleotides by solid-phase synthesis. Furthermore, we describe the impact of c^1^I on the thermodynamic stability of RNA double helices, determined by UV spectroscopic melting experiments. In addition, we describe the pairing mode of c^1^I with cytidine in short structured RNA motifs (Fig. 1A), based on investigations by nuclear magnetic resonance (NMR) spectroscopy. The study continues with an analysis of how c^1^I modifications impact the secondary structure of poly(I:C) RNA motifs. Poly(I:C) RNA is a synthetic analog of double-stranded RNA that is commonly used as a molecular mimic of viral infection. Finally, we challenge the hypothesis that a guanine residue in the active site is involved in the catalysis of phosphodiester cleavage in the twister ribozyme (Fig. 1B and C). We do so by demonstrating the complementary and unique utility of c^1^I as an atomic mutagenesis probe for this functional RNA.

## Results and discussion

### Synthesis of 1-deazainosine (c^1^I) phosphoramidite

**Scheme 1:**
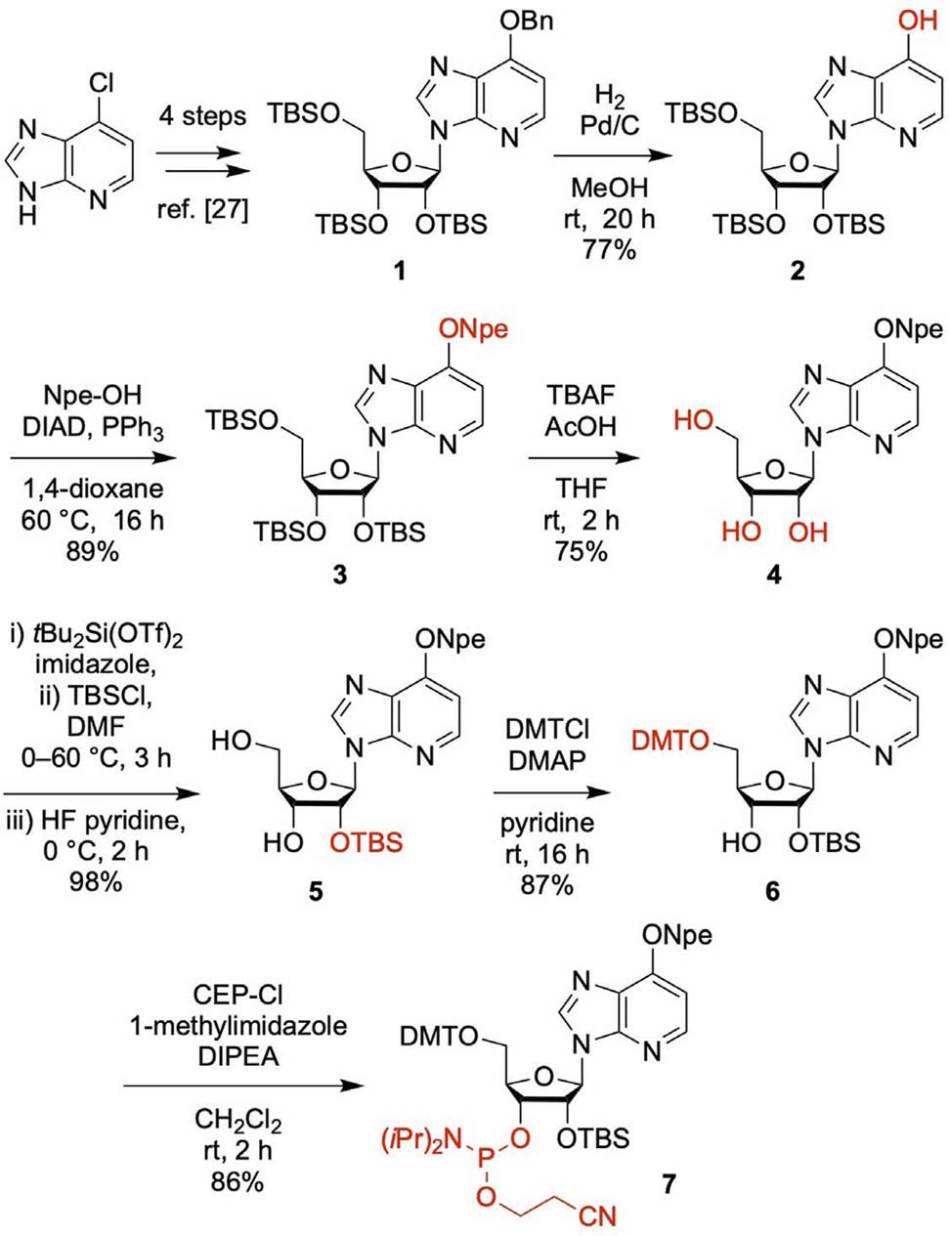
Synthesis of 1-deazainosine (c^1^I) phosphoramidite. Reaction conditions and yields as indicated. *N*,*N*-Diisopropylethyl-amine, DIPEA; (2-nitrophenyl)ethyl, NPE; diisopropyl azodicarboxlate, DIAD; trifluoroacetic anhydride, TFAA; trifluoroacetyl, Tfa; tetrabutylammonium fluoride, TBAF; *tert*-butyldimethylsilyl, TBS; 4,4′-dimethoxytrityl chloride, DMT; 4-(dimethylamino)pyridine, DMAP; cyanoethyl, CE.

The synthetic route to c^1^I phosphoramidite made use of a precursor that we introduced recently for the synthesis of 1-deazaguanosine (c^1^G) phosphoramidite.^27^ Starting from commercially available 6-chloro-1-deazapurine and 1,2,3,5-tetraacetyl-β-d-ribofuranose, the *O*^6^-benzyloxy-1-deazapurine nucleoside 1 was readily obtained in four steps (Scheme 1).^27^ Then, the benzyl protection was removed under hydrogenation conditions to release the hydroxyl group at C6. Under Mitsunobu conditions using triphenylphosphine, diisopropyl azodicarboxylate (DIAD) and 2-(*p*-nitrophenyl)ethanol (Npe-OH), the C6-OH of compound 2 was protected with a *p*-nitrophenylethyl (Npe) group to provide nucleoside 3. Subsequent deprotection of the *tert*-butyldimethylsilyl (TBS) groups with tetra-*n*-butylammonium fluoride (TBAF) afforded the triol 4. Using the Beigelmann approach,^30^ the 3′ and 5′ OH groups were first reacted with di-*tert*-butylsilyl bis(trifluoromethansulfonate) followed by selective protection of the 2′-OH group using TBS-Cl. Removal of the silyl clamp of the 3′ and 5′-OH groups with pyridine buffered hydrofluoric acid afforded the selectively 2′-*O*-TBS protected nucleoside 5. The 5′-OH was protected using 4,4′-dimethoxytrityl chloride (DMT-Cl) under standard condition to give compound 6. Finally, the phosphoramidite was synthesized under basic conditions using 2-cyanoethyl-*N*,*N*-diisopropylchlorophosphoramidite (CEP-Cl), 1-methylimidazole and diisopropylethylamine (DIPEA) to give the target compound 7. Starting from the commercially available 6-chloro-1-deazapurine, the c^1^I-phosphoramidte 7 was synthesized in ten steps, with nine chromatographic purifications and an overall yield of 17%.

### Synthesis of c^1^I-modified RNA

RNAs with site-specific c^1^I modifications were synthesized on solid-phase using the new building block 7 together with 2′-*O*-TBS protected A, C, G U phosphoramidites, or alternatively, with 2′-*O*-[(triisopropylsilyl)oxy]methyl protected (TOM) amidites.^31,32^ The novel building block was coupled with yields higher than 98% according to the trityl assay. The cleavage of the oligonucleotides from the solid support and deprotection were conducted using methylamine/ammonia in water (AMA), followed by treatment with tetra-*n*-butylammonium fluoride (TBAF) in tetrahydrofuran. Salts were removed by size-exclusion chromatography, and RNAs were purified by anion-exchange chromatography under denaturating conditions (80 °C column temperature; SI Fig. 1 and Table S1). The molecular weights of the purified RNAs were confirmed by liquid chromatography (LC) electrospray-ionization (ESI) mass spectrometry (MS). The sequences of c^1^I containing RNAs synthesized in the course of this study are listed in SI Table S1 and (see also SI Fig. 1B)

### Impact of c^1^I on RNA base pairing stability

**Fig. 2:**
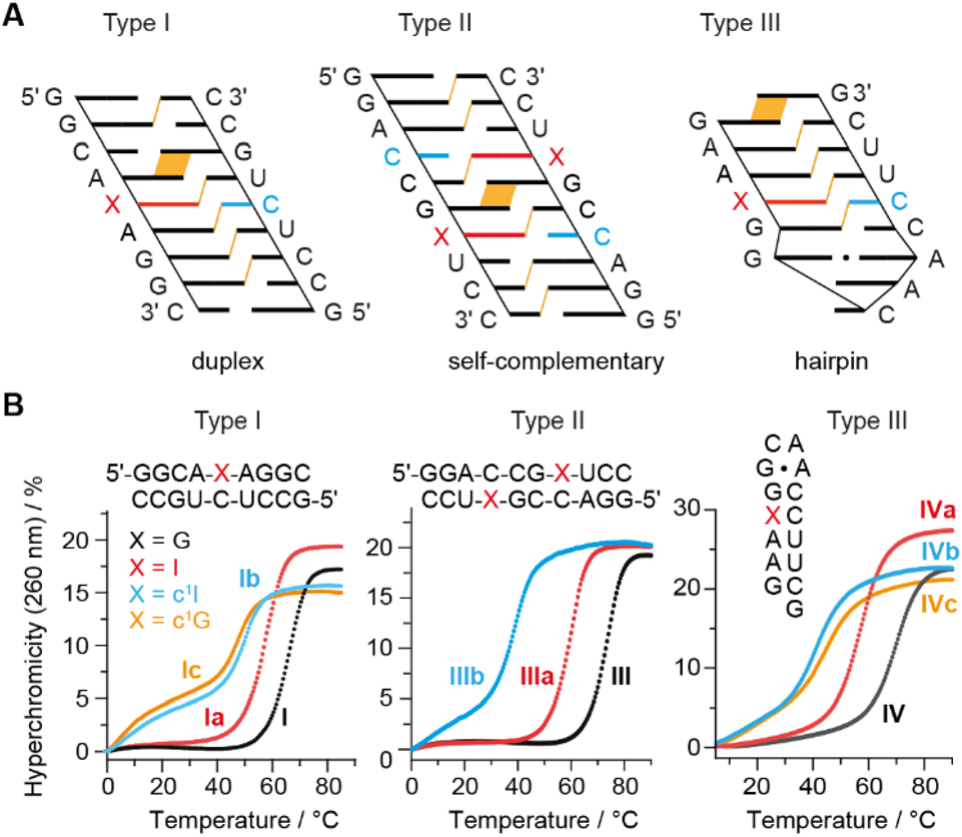
UV spectroscopic RNA melting study. (A) Sequence design for the thermodynamic analysis of RNA double helices containing c^1^I–C base pairs (types I, II and III). The cartoon presentations highlight inter-strand stacking interactions (in orange). (B) Exemplary UV melting profiles of c^1^I modified RNAs and reference systems. For conditions see Table 1 and SI.

In RNA double helices, inosine can pair with cytidine (I–C) in a typical *cis*-Watson–Crick geometry,^33^ and the pairing strength of I–C is comparable to a standard adenosine–uridine (A–U) pair.^34^ The replacement of inosine by c^1^I is expected to impair pairing strength because the N1–H of I is replaced by C–H, thereby depriving the capability for the formation of a strong hydrogen bond with N3 of C (Fig. 1A). To investigate the thermodynamic impact of c^1^I in more detail we designed different types of RNA double helices as shown in Fig. 2A. The first motif (Type I) represents a bimolecular duplex of nine base pairs with the target base interaction in the duplex center. The second RNA motif (Type II) consists of a palindromic RNA of ten base pairs with two target interactions separated by two standard base pairs. The type II design is very sensitive for the impact arising from a modification on base pairing. With only two or three regular Watson–Crick base pairs next to the modification, the nucleation of such duplexes can become substantially hindered.^35,36^ Thus, these RNA palindromes are anticipated to strongly respond to a base modification reflected in changes of the thermodynamic pairing parameters (*T*_m_, Δ*G*, Δ*H*, Δ*S*). The third RNA motif (Type III) is a hairpin with a GCAA loop (extra stable GNRA) and the modification residing in the center of its short stem.

The thermodynamic data we obtained for the three RNA systems by UV-spectroscopic melting profile measurements are illustrated in Fig. 2B and summarized in Table 1 (for the corresponding melting profiles, see the SI Fig. S2 to S5).^37–39^ The type I RNA I melts at 67.7 °C (Fig. 2B). When the central guanosine of the G–C base pair (three hydrogen bonds) is replaced by inosine (I–C, two hydrogen bonds), the duplex (Ia) is destabilized by 9.3 °C. This is about the same extent of destabilization as observed for the replacement of G–C by A–U (Δ*T* = −8.2 °C, Δ*G* = 1.8 kcal mol^−1^).^40^ Placing c^1^I opposite of cytosine (Ib) destabilizes the duplex further by 10.3 °C (Δ*G* = 2.5 kcal mol^−1^) which is consistent with the formal reduction of hydrogen bonds in the Watson–Crick mode to just one. We further compared the c^1^I–C containing duplex to the previously investigated c^1^G–C containing duplex and found that latter is only 2.8 °C more stable (Δ*G* = −0.5 kcal mol^−1^).^27^

**Table 1:** Thermodynamic parameters of modified RNAs (and references) obtained by UV melting profile analysis^a^

#	RNA sequence^a^	*T*_m_ [°C]	Δ*G*^0^ [kcal mol^−1^]^b^	Δ*H*^0^ [kcal mol^−1^]^b^	Δ*S*^0^ [cal mol^−1^ K^−1^]^b^
I	5′ GGCA**G**AGGC	67.7	−17.2 ± 0.3	−84.0 ± 2.0	−224 ± 6
	3′ CCGUCUCCG				
Ia	5′ GGCA**I**AGGC	58.4	−15.1 ± 0.5	−83.3 ± 8.2	−228 ± 20
	3′ CCGUCUCCG				
Ib	5′ GGCA**c**^1^**I**AGGC	48.1	−12.6 ± 0.8	−81.2 ± 8.2	−230 ± 25
	3′ CCGUCUCCG				
Ic^c^	5′ GGCA**c**^1^**G**AGGC	50.9	−13.1 ± 0.9	−79.9 ± 7.7	−224 ± 23
	3′ CCGUCUCCG				
II	5′ GGCUA**G**CC	60.3	−15.4 ± 0.3	−81.7 ± 2.8	−223 ± 10
IIa	5′ GGCUA**I**CC	42.1	−10.3 ± 0.1	−65.1 ± 3.4	−184 ± 11
IIb^d^	5′ GGCUA**c**^1^**I**CC^d^	21.9	−6.2 ± 0.6	−50.2 ± 8.4	−148 ± 26
III	5′ GGACCG**G**UCC	74.7	−18.7 ± 0.9	−83.9 ± 6.3	−219 ± 18
IIIa	5′ GGACCG**I**UCC	60.2	−14.4 ± 0.1	−72.7 ± 0.3	−195 ± 1
IIIb	5′ GGACCG**c**^1^**I**UCC	42.2	−11.4 ± 0.9	−93.4 ± 7.2	−274 ± 21
IV	5′ GAA**G**G-GCAA-CCUUCG	71.0	−7.5 ± 0.2	−57.9 ± 1.8	−169 ± 5
IVa	5′ GAA**I**G-GCAA-CCUUCG	57.4	−4.9 ± 0.1	−50.7 ± 0.9	−154 ± 3
IVb	5′ GAA**c**^1^**I**G-GCAA-CCUUCG	41.3	−2.7 ± 0.6	−41.8 ± 8.8	−131 ± 29
IVc^c^	5′ GAA**c**^1^**G**G-GCAACCUUCG^c^	44.8	−2.5 ± 0.3	−38.0 ± 4.5	−119 ± 14
V	5′ I_15_C_15_	50.4	−5.2 ± 0.4	−57.3 ± 3.3	−174 ± 10
Va	5′ I_7_C_7_	28.5	−7.8 ± 0.2	−88.4 ± 2.3	−271 ± 7
Vb	5′ I_6_**c**^1^**I**C_7_	9.9	−3.7 ± 0.3	−56.8 ± 4.6	−178 ± 15

a Buffer: 10 mM Na_2_HPO_4_, 150 mM NaCl, pH 7.0. *T*_m_ values are listed at a concentration of 12 µM RNA (calculated from concentration dependent measurements; see 1/*T*_m_*vs.* ln c plots in the SI). The estimated errors of UV-spectroscopically determined *T*_m_ values are ±0.2 °C. Δ*H* and Δ*S* values were obtained by van't Hoff analysis according to refs (37–39). Errors for Δ*H* and Δ*S*, arising from noninfinite cooperativity of two-state transitions and from the assumption of a temperature-independent enthalpy, are typically 10–15%. Additional error is introduced when free energies are extrapolated far from melting transitions; errors for Δ*G* are typically 3–5%.

b At 298 K.

c Thermodynamic data of c^1^G reference RNAs were taken from our earlier study, ref. 27.

d This RNA avoids a direct C-c^1^I interaction by frameshifting: see main text and Fig. 5.

The same trend of thermodynamic stabilities for inosine and 1-deazainosine modifications was also found in type II palindromic RNAs II and III (see *e.g.*, III to IIIc; G replaced by I results in: Δ*T* = −7.3 °C per bp, Δ*G* = 2.15 kcal mol^−1^ per bp; I replaced by c^1^I results in: Δ*T* = −9.0 °C per bp, Δ*G* = 1. 5 kcal mol^−1^ per bp) and type III hairpin RNA (IV to IVc; G replaced by I results in: Δ*T* = −13.5 °C, Δ*G* = 4.0 kcal mol^−1^; I replaced by c^1^I: Δ*T* = −16.0 °C, Δ*G* = 3.0 kcal mol^−1^; c^1^I replaced by c^1^G results in: Δ*T* = 3.1 °C per bp, Δ*G* = −0.3 kcal mol^−1^ per bp) (Fig. 2B and Table 1).

At this point, we mention that we measured the hairpin system (IV, IVa, and IVb) at different NaCl concentrations. As expected, the melting temperature increased with increasing concentrations of NaCl (SI Fig. S6). We found no indication for a change of the c^1^I–C pairing mode within the NaCl concentration range measured.

The thermodynamic data obtained in this study is consistent with earlier studies on the thermodynamics of I–C containing oligoribonucleotides that focused on the determination of nearest neighbor parameters.^34^ Our data is also consistent with a comparative study analyzing the loss of one hydrogen bond in A–U *versus* P–U (purine–uridine) base pairs^41^ that resembles the situation of I–C *versus* c^1^I–C containing RNA, although the extent of destabilization was higher for I–C (two H-bonds) *vs.* c^1^I–C (one H-bonds) (Ia*vs.*Ib: 
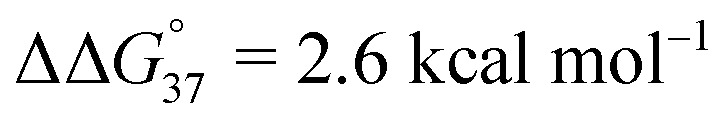
) compared to A–U (two H-bonds) *vs.* P–U (one H-bonds) (see first entry in Table 2 in ref. 41: 
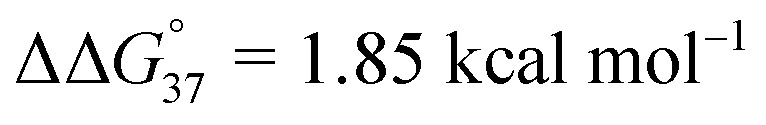
).

This observation might be rationalized by the fact that c^1^I has an altered heterocyclic core (compared to I) with reduced stacking propensity, in contrast to P whose heterocyclic core remains unchanged (compared to A).

**Fig. 3:**
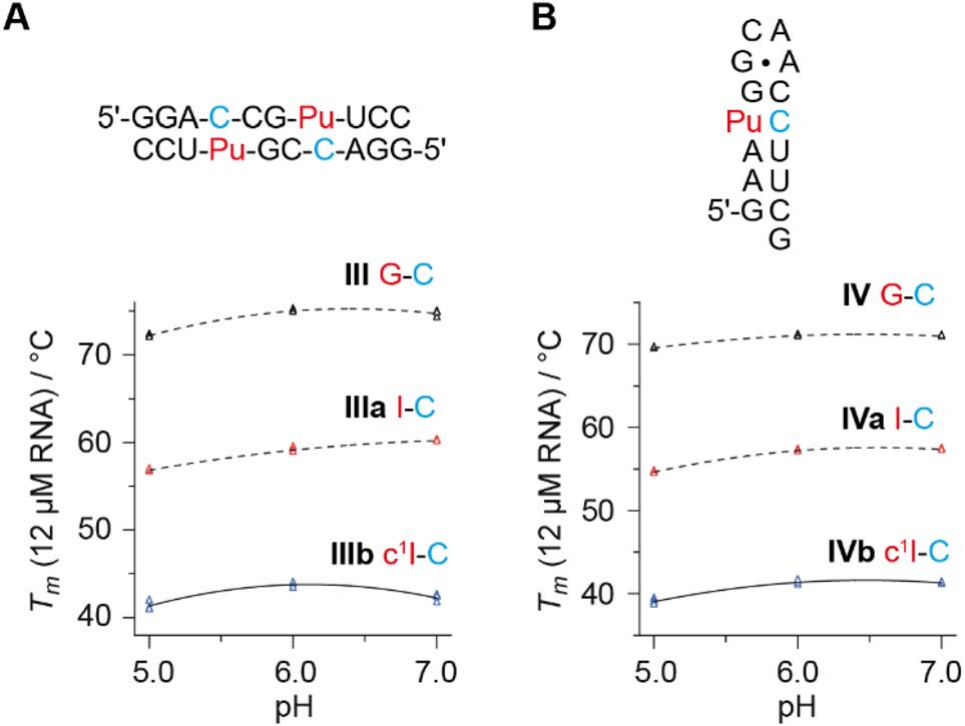
pH dependent UV-melting analysis of c^1^I modified RNAs and reference systems. (A) *T*_m_*vs.* pH plot for palindromic RNAs III (G–C), IIIa (I–C) and IIIb (c^1^I–C). (B) *T*_m_*vs.* pH plot for hairpin RNAs IV (G–C), IVa (I–C) and IVb (c^1^I–C). For the palindromic RNAs, data points were calculated for an RNA duplex concentration of 12 µM from 1/*T*_m_*vs.* ln c plots; see the SI). *T*_m_ values for the hairpins were confirmed to be concentration independent. Individual data points (open triangles) (*n* = 3 independent experiments). Buffer: 10 mM Na_2_HPO_4_, 150 mM NaCl, pH as indicated.

Notably, for c^1^I opposite to C, a *trans* Watson–Crick Hoogsteen base pair geometry with a N3-protonated C is conceivable as an alternative to the Watson–Crick pairing mode (Fig. 1). For c^1^I, the Hoogsteen pairing mode even seems to be advantageous because of the formation of two hydrogen bonds instead of only one (Fig. 1A). To find evidence for this potential pairing alternative, we performed pH-dependent UV melting experiments as shown in Fig. 3. However, since the observed *T*_m_ values showed only little dependence on the pH of the melting buffer (2 to 3 °C), the involvement of a protonated C species in base pairing seems unlikely; if a protonated C species were involved, a pronounced increase in *T*_m_ with decreasing pH is expected (∼6 to 8 °C), as recently observed for pairing of c^1^c^3^G to protonated C,^28^ or for xanthosine pairing to protonated cytosine.^40^

Further evidence against a Hoogsteen-like pairing mode of c^1^I–C, and in favor of a Watson–Crick-like geometry, originates from NMR spectroscopy as described below.

### NMR spectroscopy of inosine and 1-deazainosine containing RNA

Watson–Crick base-pairs are easily detectable by ^1^H-NMR spectroscopy. The signals for hydrogen-bonded protons (‘imino protons’) directly reflect the formation of double helices within folded RNA.^41–43^ The chemical shifts of these signals are characteristic for A–U (>13.5 ppm) and C–G base pairs (∼11.5–13.5 ppm), and the linewidths reflect proton exchange with the solvent. The chemical shifts of imino protons are sensitive to chemical modifications, in particular, if the modification concerns the nucleobase.

**Fig. 4:**
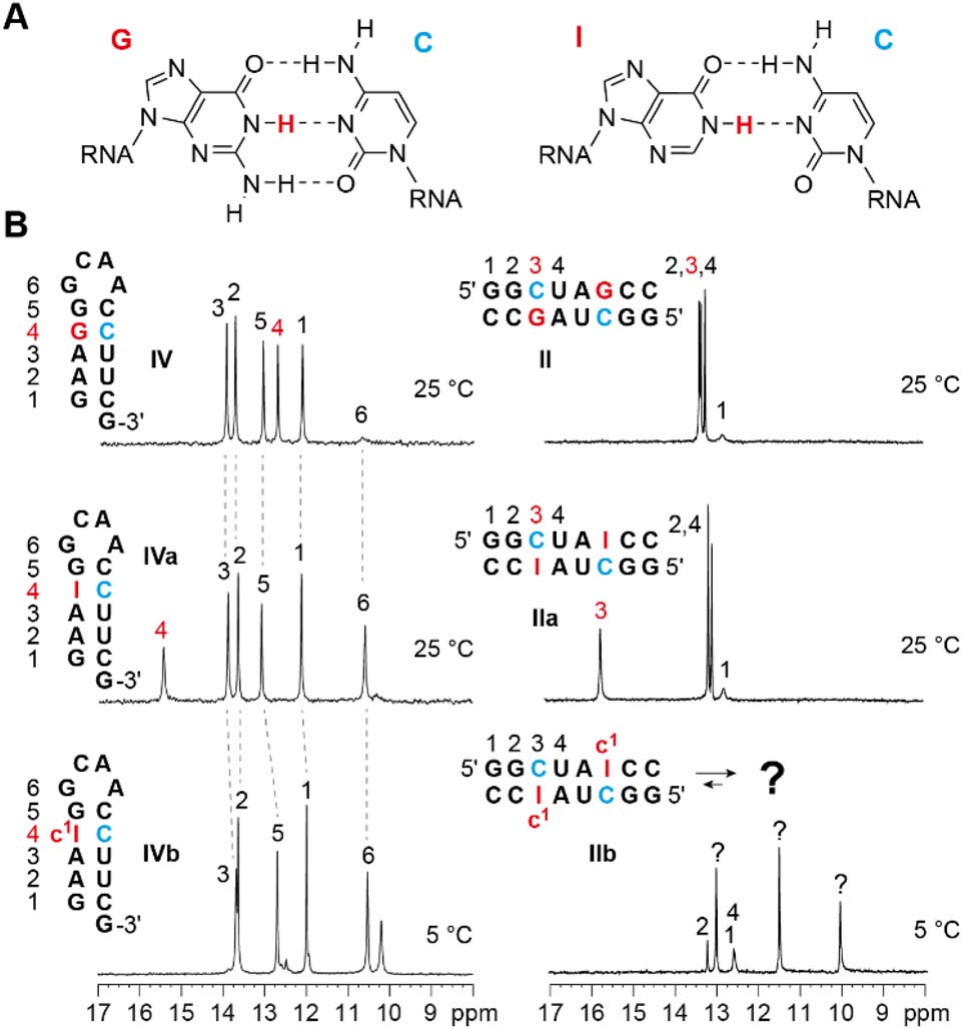
Comparative ^1^H-NMR imino proton spectroscopy of I and c^1^I modified RNA. (A) Structures of G–C and I–C Watson–Crick base pairs (top). (B) ^1^H NMR spectra of RNA hairpins IV, IVa and IVb (left) and self-complementary RNA duplexes II, IIa and IIb (right) with either G, I or c^1^I (red color), recorded at 25 °and 5 °C, respectively. Note that IIb gives a significantly distinct imino proton pattern compared to the references II and IIa, while this is not the case for IVb when compared to IV and IVa. Conditions: c(RNA) = 0.5 mM, 25 mM NaCl, 15 mM sodium phosphate buffer, H_2_O/D_2_O 9/1, pH 6.5. Numbers refer to full base pairs (and not to single nucleotides in the RNA sequence).

Comparative imino proton ^1^H NMR spectra of the hairpin 5′-GAAGG-GCAA-CCUUCG IV and the corresponding I–C, and c^1^I–C modified counterparts, IVa and IVb, are depicted in Fig. 4. They show that I–C forms a well-defined interaction at room temperature, characterized by a resonance that was shifted from 12.70 ppm (G4-C in IV) to 15.45 ppm (I4-C in IVa). All other standard Watson–Crick base pairs retained their imino proton chemical shifts (Fig. 4A, left panel; and SI Fig. 7A), indicating that an I–C pair integrates well into an A-form RNA double helix, with little effect on the neighborhood. This is consistent with previous studies reported in the literature that found that I and C can form base pairs that are isosteric to standard (*cis*) Watson–Crick pairs.^44,45^

The behavior was different for hairpin IVb which contained a c^1^I opposite to C. At room temperature, the pronounced thermodynamic destabilization is reflected in a substantial broadening of all imino proton resonances in the stem, although their chemical shift values were essentially unaffected (SI Fig. 7A). Only when the temperature was reduced to 5 °C (Fig. 4B), sharp signals were detected for all Watson–Crick base pairs and the characteristic G–A pair in the hairpin loop.^46,47^ No other resonances that would directly suggest a hydrogen-bonded c^1^I–C4 base pair (*e.g.* Hoogsteen pair involving a protonated C; see Fig. 1A) were observed in the imino proton region.

**Fig. 5:**
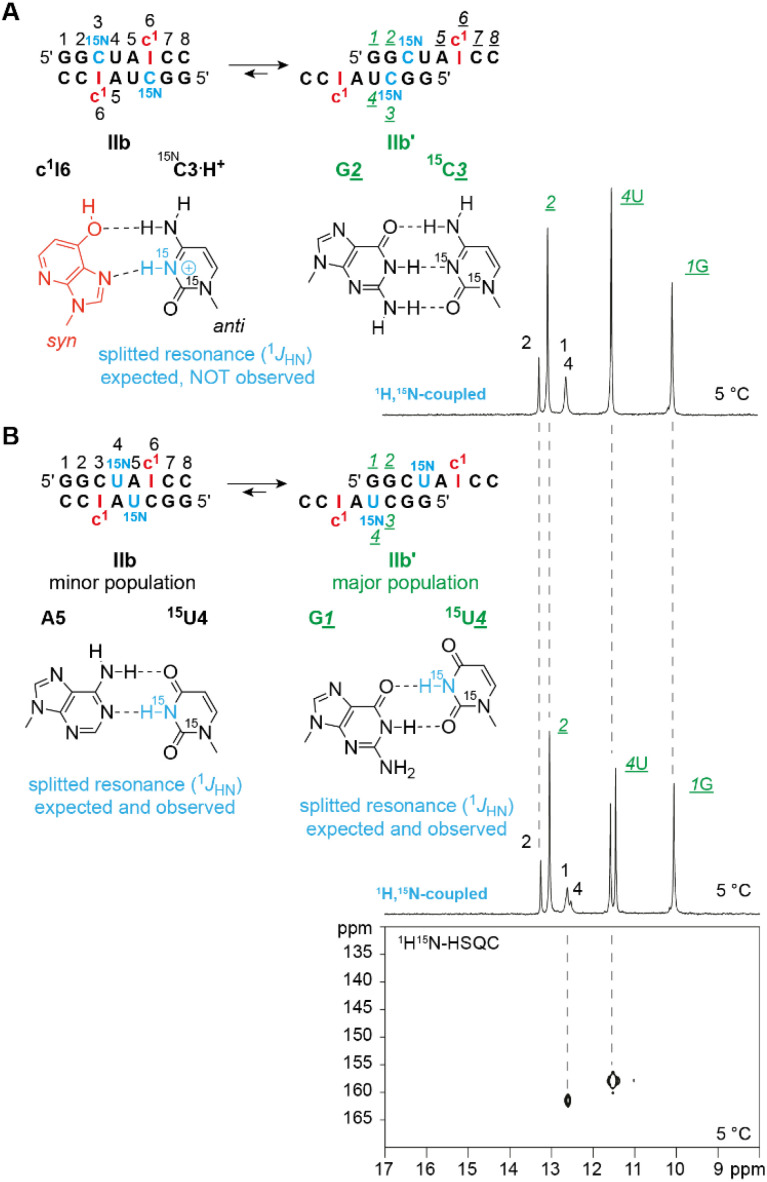
The palindrome GGCUAc^1^ICC prefers a 4-base pair (IIb′) over an 8-base pair (IIb) register in a temperature dependent equilibrium (also see the SI Fig. 7A). Evidence comes from an ^1^H,^15^N coupled ^1^H-NMR spectroscopic comparison of (A) GG^15N^CUAc^1^ICC with a single ^15^N labelled cytosine and (B) GGC^15N^UAc^1^ICC with a single ^15^N labelled uridine. For ^1^H,^1^H-NOESY and ^1^H,^15^N-HSQC NMR spectra see the SI. Conditions: c(RNA) = 0.5 mM, 25 mM NaCl, 15 mM sodium phosphate buffer, H_2_O/D_2_O 9/1, pH 6.5. Numbers refer to nucleotides in the RNA sequence.

To shed further light on the c^1^I–C base interactions in a double helix, we first investigated a short palindromic duplex of eight base pairs (GGCUAGCC, II), in which G3 was replaced by I and c^1^I, respectively. For the inosine containing duplex IIa we observed again a resonance shift of almost 3 ppm that was attributed to the formation of two I–C base pairs (Fig. 4B). For the c^1^I containing duplex IIb, however, we observed a distinct resonance pattern that, according to ^1^H,^1^H-NOESY and ^1^H,^15^N HSQC NMR spectra, suggested the formation of G–U wobble base pairs (SI Fig. 7B). This hypothesis was confirmed by using U4-^15^N3 and C3-^15^N3 labelled palindromes, respectively, for ^1^H,^15^N-coupled NMR experiments (Fig. 5). The c^1^I–C mismatches destabilize the fully paired duplex to such an extent that the formation of a frameshifted duplex (of four base pairs: 2 G–C, 2 G–U) with 3′ overhangs becomes thermodynamically favorable (Fig. 5 and SI Fig. 7B).

**Fig. 6:**
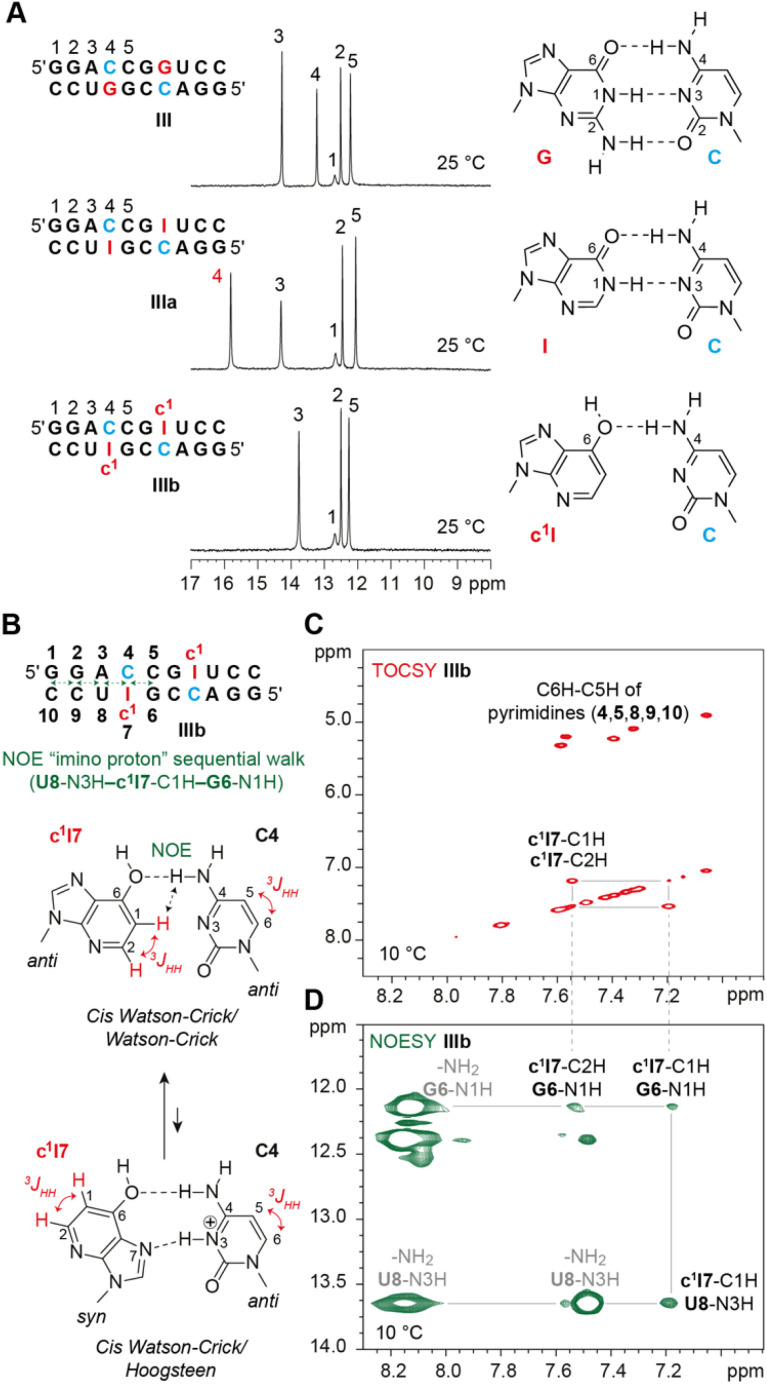
NMR analysis of a 10 nt I and c^1^I modified RNA palindrome. (A) ^1^H NMR spectra self-complementary RNA duplexes III, IIIa and IIIb (left) with either G, I or c^1^I (red color), recorded at 25 °C. Structures of Watson–Crick base pairs of G–C, I–C, and putative isosteric base pairing of c^1^I–C (right); numbers refer to base pairs. (B) Approach to validate the prevalent base pair mode of c^1^I–C of RNA IIIb; numbers refer to nucleotides in the RNA sequence. In Watson–Crick geometry, the “imino proton” sequential walk (^1^H,^1^H-NOESY) should show a correlation between the c^1^I7-C1H to the neighbouring G6-N1H and U8-N3H imino resonances. (C) Assignment of c^1^I7-C1H and c^1^I7-C2H in IIIb by ^1^H,^1^H-TOCSY experiments. (D) The ^1^H,^1^H-NOESY spectrum of IIIb indeed shows the anticipated cross peaks for c^1^I7-C in Watson Crick mode (for complete spectra see the SI Fig. S9 to S11). Conditions: c(RNA) = 0.5 mM, 25 mM NaCl, 15 mM sodium phosphate buffer, H_2_O/D_2_O 9/1, pH 6.5.

To learn more about the actual base pairing mode of c^1^I–C, we examined the 10 nt palindromic duplex (GGACCGGUCC, III) where we expected no escape possibility for the intended c^1^I replacement. RNA III exhibited the anticipated five imino proton resonances in the ^1^H NMR spectrum (Fig. 6A). When G7 (III) was substituted with I7 (IIIa), the characteristic imino proton shift of nearly 3 ppm was evident (Fig. 6A). Finally, replacing I7 with c^1^I7 (IIIb) resulted in the loss of the corresponding imino signal while all other resonances were retained (Fig. 6A). This is consistent with the notion that the base pairs around the c^1^I–C mismatch were unaffected and fully formed, even at 25 °C.

At this point, we mention that we measured pH dependent NMR spectra of the palindrome IVb to reveal potential protonation effects on cytidine. However, even at pH 5.2, we found no indication of a protonated cytosine involved in a Hoogsteen base pair (Fig. 1A, SI, S8).

Furthermore, we hypothesized that c^1^I–C in Watson–Crick geometry would result in a “sequential walk” of imino protons (accessible by ^1^H,^1^H-NOESY spectroscopy), evidenced by a potential correlation between the c^1^I7-C1H resonance and the neighboring G6-N1H and U8-N3H imino resonances. There could also be an interbase NOE between c^1^I-C1H and C4-NH_2_ (Fig. 6B). To this end, we first assigned the c^1^I7-C1H and C2H using ^1^H,^1^H-TOCSY experiments (Fig. 6C). Even more exciting was observing correlations between the c^1^I7-C1H resonance and the neighboring G6-N1H and U8-N3H imino resonances in the ^1^H,^1^H-NOESY spectrum (Fig. 6D). These results provide good evidence that the c^1^I7-C4 base pair populates a Watson–Crick-like geometry to a significant extent rather than a Hoogsteen-pair conformation (Fig. 6B). For the latter, the c^1^I7-C1H hydrogen atom is too distant to the imino protons of G6-N1H and U8-N3H to provide pronounced NOEs (Fig. 6B).

Finally, we selected the GAAIG-GCAA-CCUUCG (IVa) *vs.* GAAc^1^IG-GCAA-CCUUCG (IVb) hairpin system for additional NMR experiments, with the aim for a more detailed NMR structure determination. High quality data spectra were obtained for the I4-C11 RNA IVa, (SI Fig. S12), and the NOE, scalar coupling and residual dipolar coupling data was used to determine the solution structure (PDB ID 30SC; SI Fig. S13 and Table S3). For the corresponding c^1^I modified RNA IVb, the NMR data set strongly suggests that the sequence parts flanking the c^1^I4-C11 pair are in A-form helical arrangement (SI Fig. S14). Unfortunately, for the c^1^I4-C11 base pair in IVb, we found a very limited number of NOEs indicating a high flexibility (*i.e.* an exchange process on the intermediate time scale leading to broad resonances) with the two nucleotides c^1^I4 and C11 sampling various conformational states leading to an ill-defined structure (SI Fig. S14A). We further could not detect analogous NOE cross-peaks for the c^1^I4-C11 pair to the direct adjacent base pairs in hairpin IVb, likely due to increased local dynamics relative to the palindromic duplex IIIb.

### Poly I:C RNA oligos and impact of c^1^I

Polyinosinic:polycytidylic acid, commonly referred to as poly I:C, is a synthetic analog of double-stranded RNA.^48^ It is widely used in scientific research as a molecular mimic of viral infection because dsRNA is a common intermediate in the replication of many viruses.^49^ In immune stimulation, poly I:C is recognized specifically by Toll-like receptor 3 (TLR3), located in endosomes, or by RIG-I-like receptors, such as MDA5, found in the cytoplasm.^50^ Activation of these receptors triggers downstream signaling pathways, leading to the production of type I interferons (*e.g.*, IFN-α and IFN-β) and pro-inflammatory cytokines.^51^

**Fig. 7:**
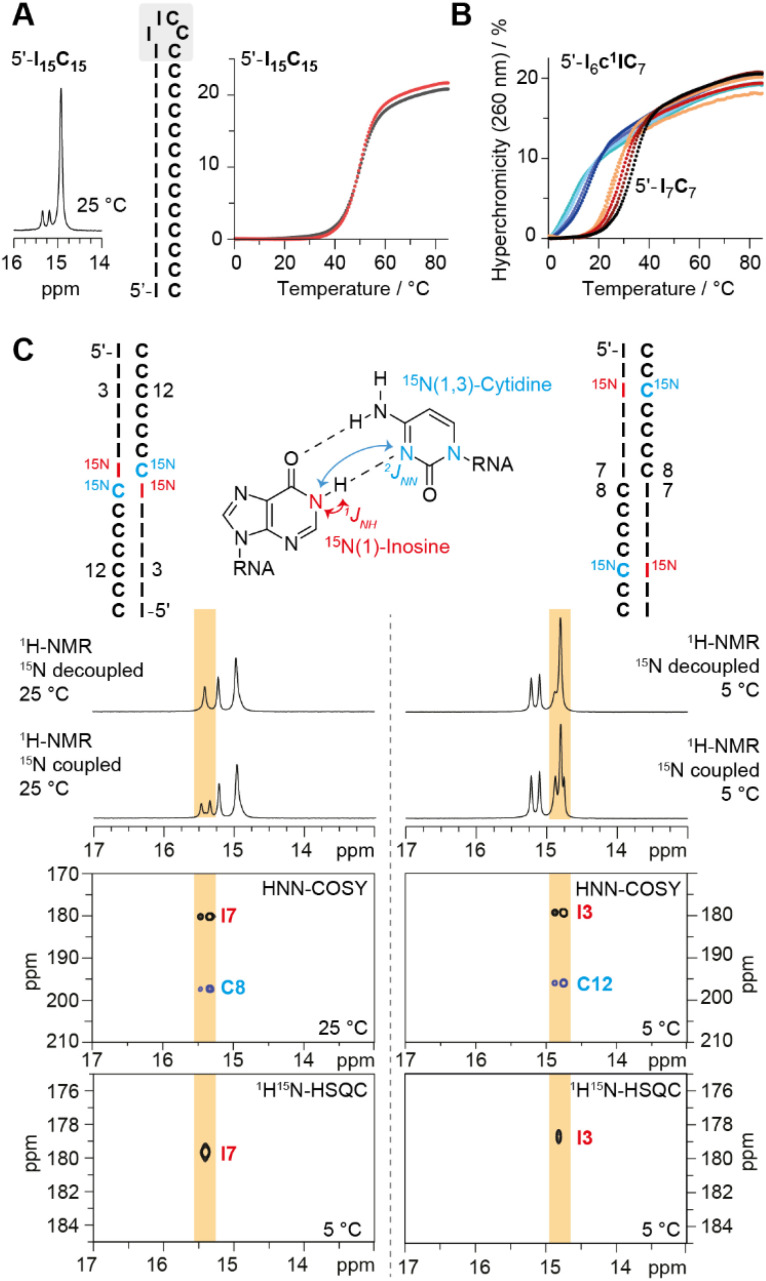
Characterization of poly-I:C RNA oligos. (A) 5′-I_15_C_15_ (V): ^1^H-NMR imino proton spectrum, secondary structure, and UV melting profiles at two different RNA concentrations (2 and 12 µM). (B) Concentration-dependent UV-melting profiles of 5′-I_7_C_7_ (Va) (red bundle) and 5′-I_6_c^1^IC_7_ (Vb) (blue bundle). (C) Site-specifically ^15^N-labelled 5′-I_7_C_7_ RNAs (^15^N patterns as indicated): ^1^H NMR, HNN, and HSQC spectra verify I–C base pair formation in Watson–Crick geometry in strand positions 7,8 and 3,12, respectively.

To investigate the base pairing properties of poly I:C RNA, we first synthesized the self-complementary model system I_15_C_15_ (V) by solid-phase synthesis and recorded a ^1^H NMR spectrum (Fig. 7A). It showed a major signal at 14.9 ppm and two minor ones at slightly higher chemical shifts, consistent with the formation of IC base pairs. Further, UV melting experiments gave biphasic sigmoid-shaped melting profiles with a defined *T*_m_ value of 50.4 °C. The melting was independent from RNA concentration suggesting a monomolecular process, which is compatible with a hairpin secondary structure of I_15_C_15_.

**Fig. 8:**
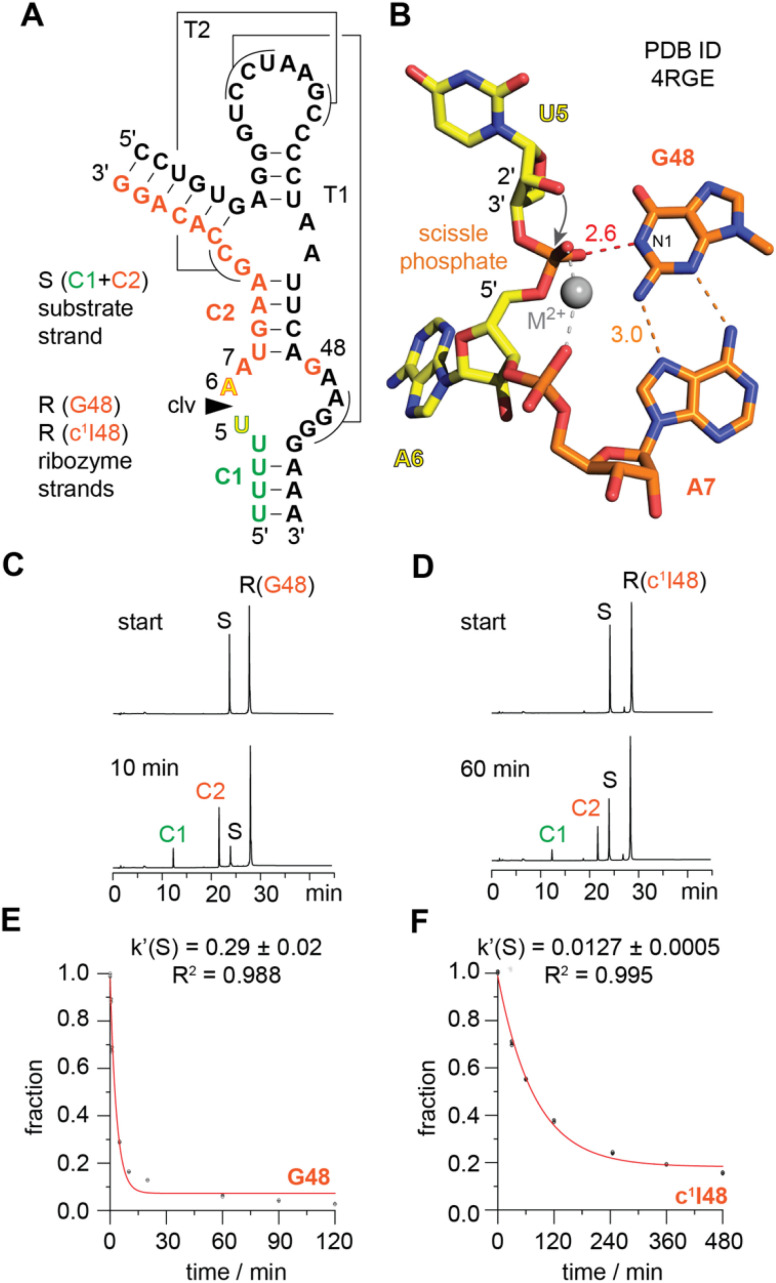
Atomic mutagenesis of the twister ribozyme: impact of an active site G-to-c^1^I mutation on activity to elucidate the mechanism of the phosphodiester cleavage. (A) Secondary structure of the two-strand ribozyme assembly used for functional cleavage assays. (B) X-ray structure of the twister ribozyme (precatalytic state; PDB ID 4RGE); interactions of guanine-48 with the scissile phosphate and A7 is shown; the 2′-OH nucleophile is modeled on dU5; distances in Å. HPLC traces of wild-typeG48 (D) and c^1^I48 modified (D) ribozyme at two time points illustrate that product formation of the c^1^I-modified ribozyme is impeded under otherwise same reaction conditions. Cleavage rate determination of wild-type G48 (E) and c1IG48 (F) ribozymes.

Interestingly, when we investigated the shorter I_7_C_7_ oligo (Va) by UV melting experiments we found a lower, and importantly, concentration-dependent melting point (Fig. 8B), consistent with a bimolecular process (duplex formation). Furthermore, the modification of the central I–C/C–I pairs by c^1^I–C/C-c^1^I (I_6_c^1^IC_7_, Vb) did not result in a switch to hairpin structures but only lowered the stability of the duplex. This is supported by the concentration dependence of the melting points and an under lying bimolecular melting process (Fig. 8B). Only for very low concentrations of I_6_c^1^IC_7_ (<5 µM), the melting profiles were biphasic, suggesting a hairpin duplex equilibrium.

To better understand the pairing properties of the I_7_C_7_ strand, we performed NMR experiments with stable isotopically labelled RNA strands. Direct evidence for hydrogen-bonds in a base pair interaction can be obtained from HNN-COSY experiments that were originally introduced by Dingley and Grzesiek.^52^ The size of these cross hydrogen-bond scalar couplings (^2^*J*_NN_ = 6–7 Hz) allows efficient magnetization transfer to correlate chemical shifts in base pairs.^52^ To date, a large number of experiments are available to measure *J*-couplings across hydrogen bonds in nucleic acids.^53–61^ For weak base pairing interactions, the BEST-sellr experiment (originally introduced by the Sattler group)^62^ provides a large sensitivity improvement for hydrogen-bond correlation experiments by the optimizations of magnetization transfers in combination with the detection of non-exchangeable protons. This experiment enables the detection of weak and transient A–U base pairs, and we therefore considered it appropriate for studying I–C interactions as well.^63,64^

We evaluated the formation of the central I–C/C–I pairs of I_7_C_7_ in solution, using a chemically synthesized, site-specifically labeled RNA with ^15^N(1)-I7 and ^15^N(1,3)-C8 (Fig. 7C, left panel). If I_7_C_7_ forms a duplex (and not a hairpin) then these interactions (^15^N(1)-I7:^15^N(1,3)-C8 base pairs) should be directly detectable. Indeed, in the ^1^H,^15^N HSQC spectrum of the RNA, we observed one resonance for I7, with a clear correlation to C8 in the HNN COSY spectrum (Fig. 7C, left panel). This observation suggests that a major population of fully paired, self-complementary duplex exists in solution. These experiments also assigned of the most down field-shifted I–C resonance to I7-C8.

The second, site-specifically labelled RNA of I_7_C_7_ contained the nucleosides ^15^N(1)-I3 and ^15^N(1,3)-C12. In a paired duplex, the corresponding base pairs of ^15^N(1)-I3:^15^N(1,3)-C12 are positioned within the continuous purine-pyrimidine tracks. As expected, the ^1^H,^15^N HSQC spectrum of the RNA showed one resonance for I3, with a correlation to C12 in the HNN COSY spectrum.

In summary, the above experimentation reveals the intricate conformational interplay of poly I:C RNA pairing for the first time and contributes to a better understanding of the sequence requirements for secondary structure switches of these highly relevant biological RNAs.

### Atomic mutagenesis with c^1^I to explore ribozyme catalysis

One of the main benefits of c^1^I is that it creates new opportunities to evaluate the role of guanines in RNA general acid/base catalysis.^29^ In particular, c^1^I's application in atomic mutagenesis experiments enables investigations of the chemical mechanism of self-cleaving ribozymes. The reaction is thought to pass through a pentavalent phosphorane transition state that can become stabilized by interactions with the Watson–Crick face of a conserved guanine in the active site (β-catalysis) (Fig. 1B).^29,65,66^ However, these guanines may also facilitate deprotonation of the attacking 2′-OH (γ-catalysis) (Fig. 1C) or be involved in both β- and γ-catalysis.^67^

Compared to guanine, the nucleobase c^1^I lacks an N1 atom, which is replaced by a carbon atom (Fig. 1). This deletion accounts for a “general base knockout” and consequently eliminates acid/base properties of this position. Additionally, the exocyclic C2-NH_2_ is absent, which can play a primary role in β- (Fig. 1B) but not in γ-catalysis (Fig. 1C).

For an exemplary c^1^I atomic mutagenesis study, we focus on the well-investigated Twister (Tw) ribozyme (Fig. 8).^68^ Proton transfer from the protonated N3 of a conserved adenine (A6) at the cleavage site to the 5′-O leaving group is the primary factor in catalysis of this ribozyme (δ-catalysis) (Fig. 8B). This was shown previously based on the replacement of this adenine by 3-deazaadenine (c^3^A) which rendered Twister inactive.^15,69^

In contrast, the contribution of a conserved active site guanine (G48) remained less clear for a long time. This issue was addressed only very recently with greater precision.^27,28^ When 1-deazaguanosine (c^1^G48) was introduced in the active site, the “general base knockout” resulted in a rate reduction of approximately 10^2^.^27^ When 1,3-dideazaguanosine (c^1^c^3^G48), the resulting rate reduction was approximately 10^5^.^28^ The latter was attributed to the perturbation of c^1^c^3^G in forming the conserved G48-A7 base pairing interaction (Fig. 8B), in addition to its direct impact on β/γ-catalysis.

We expected the c^1^I48 variant of Twister to exhibit a reduced rate, and this expectation was met. However, the extent of the reduction was rather small (approximately 25-fold) for a mutant belonging to the “general base knockout” family in which N1 is absent (Fig. 1C). Additionally, the absence of exocyclic C2-NH_2_ (compared to the native G, and the mutants c^1^G and c^1^c^3^G) appears to reduce the likelihood of transition state stabilization (Fig. 1B). To gain a clearer understanding, we re-investigated two additional Twister variants: inosine-48 (I48) and 2-aminopurine (Ap48).^15,69^ We confirmed the previously reported rate reductions of approximately 10^2^ to 10^3^ for I48 and approximately 10 to 10^2^ for Ap48 (see SI Fig. S15).

One possible explanation for the small rate reduction of c^1^I is that c^1^I has sufficient space to shift in the pocket, allowing the phenolic oxygen atom to take over the role of acid/base catalysis and transition state stabilization, either in the deprotonated or neutral state. However, this hypothesis needs further clarification in future studies.

## Conclusions

Deazanucleoside modifications are powerful tools for atomic mutagenesis because replacing a ring nitrogen with carbon perturbs hydrogen-bond donor/acceptor capabilities, thereby altering RNA base pairing, recognition, and catalysis. Expanding this toolbox, the present study develops a robust synthesis of an appropriately protected 1-deazainosine (c^1^I) phosphoramidite and demonstrates its efficient incorporation into RNA by solid-phase methods. Thermodynamic analyses from UV melting reveal that c^1^I–C pairs are markedly less stabilizing than the corresponding I–C pairs and can even induce shifts in base-pairing register to avoid the weakened interaction. NMR spectroscopy with selectively ^15^N3-labeled pyrimidines indicates that c^1^I–C adopts a Watson–Crick-like geometry with a single classical H-bond, rather than the conceivable two-H-bond Hoogsteen pairing with protonated cytidine.

These weakened but structurally compatible pairs can be accommodated within duplex RNA without disrupting adjacent base pairs. Complementary poly(I:C) analyses show length-dependent preferences for duplex *versus* hairpin conformations, refining our understanding of viral dsRNA mimics used in therapeutic contexts. Together, the work fills a key gap by providing the chemistry, structural characterization, and biophysical properties of c^1^I-modified RNA. It establishes c^1^I as a distinct probe whose single-H-bond pairing modulates helix stability and register. These insights guide atomic mutagenesis designs to dissect RNA catalytic mechanisms with increased precision. Overall, c^1^I strengthens the deazanucleoside repertoire and offers predictable, context-dependent impacts on RNA structure and function.

## Author contributions

C. M. and R. B. synthesized the c^1^I phosphoramidite and carried out oligonucleotide syntheses. C. M. conducted the chemical, biochemical, and biophysical characterization of the modified RNAs. C. M. and C. K. performed the RNA NMR experiments. A. S. conducted RNA crystallization experiments. R. M., C. K., and E. E. supervised the study. R. M. conceived the project and wrote the manuscript with input from all authors.

## Conflicts of interest

CK is an advisor to and holds an ownership interest in Innotope, a company providing RNA SI-labelling products. The remaining authors declare no competing interests.

## Supplementary Material

SC-OLF-D6SC04009H-s001

## Data Availability

The data supporting this article have been included as part of the supplementary information (SI). Supplementary information is available. See DOI: https://doi.org/10.1039/d6sc04009h.
