# Supplementary material for: 1-Deazainosine-impact on RNA structure and role in exploring ribozyme catalysis
Source: Chem Sci. 2026 Jun 8;17(28):13874–83. doi: 10.1039/d6sc04009h (PMC13245881; doi:10.1039/d6sc04009h)
Supplement: SC-017-D6SC04009H-s001 [file SC-017-D6SC04009H-s001.pdf]

Supplementary Information (SI) for Chemical Science.
This journal is © The Royal Society of Chemistry 2026

Supporting Information

1-Deazainosine – Impact on RNA structure and role in exploring ribozyme catalysis

Christoph Mitteregger, Raphael Bereiter, Antoine Schramm, Eric Ennifar, Christoph Kreutz and Ronald Micura*

Contents

1. Supporting Methods 2

1.1 General procedures 2
1.2 Synthesis of 1-deazainosine phosphoramidite 2
1.3 Solid-phase RNA synthesis 15
1.4 Deprotection and purification of RNA 15
1.5 Mass spectrometric analysis of RNA 15
1.6 UV melting experiments of RNA 16
1.7 NMR sample preparation, NMR experiments, chemical shift assignment and structure calculation 16
1.8 Ribozyme cleavage assays 17

2. Supporting Table 18

Supporting Table S1 18
Supporting Table S2 19
Supporting Table S3 20

3. Supporting Figures 21

Supporting Figure S1 21
Supporting Figure S2 22
Supporting Figure S3 23
Supporting Figure S4 24
Supporting Figure S5 25
Supporting Figure S6 26
Supporting Figure S7 27
Supporting Figure S8 28
Supporting Figure S9 29
Supporting Figure S10 30
Supporting Figure S11 31
Supporting Figure S12 32
Supporting Figure S13 33
Supporting Figure S14 34
Supporting Figure S15 35

4. References 36

1 Supporting Methods

1.1 General procedures

Materials. All chemicals or reagents were purchased from commercial suppliers (Sigma-Aldrich, ABCR, VWR, Chemgenes, BioSynth, Synthonix) in the highest available quality and used without purification. Unless otherwise stated, all reactions were carried out in an argon atmosphere, Reactions were monitored using analytical thin-layer chromatography (TLC) Macherey-Nagel Polygram® SIL G/UV254 plates. 0.2 mm silica gel 60 for column chromatography was purchased from Macherey-Nagel.

NMR spectroscopy. ^{1}H, ^{13}C and ^{31}P spectra were recorded on a Bruker Ultrashield™ 400 Plus spectrometer. Chemical shifts (δ) are reported relative to tetramethylsilane (TMS) and referenced to the residual solvent signal (DMSO-d_6: 2.50 ppm (^{1}H), 39.52 ppm (^{13}C); $CDCl_3$: 7.26 ppm (^{1}H), 77.16 ppm (^{13}C)). The following abbreviations were used to denote multiplicities: s = singlet, d = doublet, t = triplet, q = quadruplet, m = multiplet, b = broad. Signal assignments are based on $^{1}H^{1}H$-COSY, $^{1}H^{13}C$-HSQC and $^{1}H^{13}C$-HMBC experiments.

HR-ESI-MS. High resolution electrospray ionisation mass spectra (HR-ESI-MS) were recorded in a positive mode on a Thermo Scientific Q Exactive Orbitrap, with ionization via electrospray at a spray voltage of 3.7 kV.

1.2 Synthesis of 1-deazainosine phosphoramidite

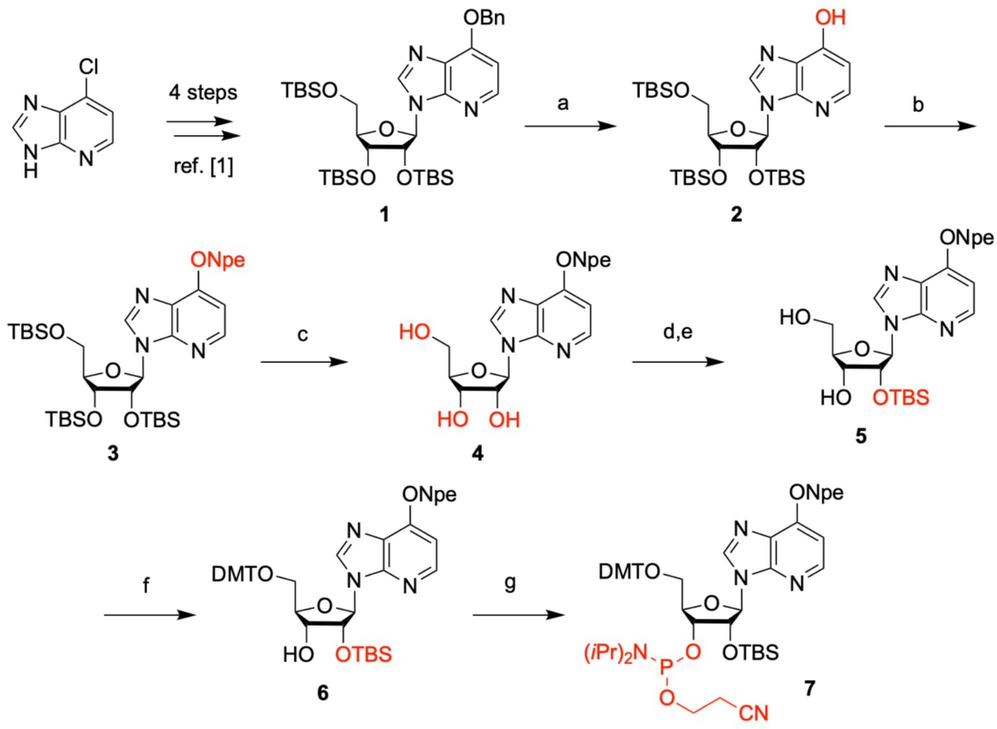

Reaction conditions: **a)** Pd/C, H_2, MeOH, r.t., 2 h, 77%. **b)** NPEOH, DIAD, PPh_3, 1,4-dioxane, 60 °C, 16 h, 89%. **c)** TBAF * 3 H_2O, HOAc, THF, r.t., 2 h, 75%. **d)** t-$Bu_2Si(OTf)_2$, imidazole, TBSCl, DMF, 0 – 60 °C, 3 h. **e)** HF, pyridine, CH_2Cl_2, 0 °C., 2 h, 98% over two steps. **f)** DMT-Cl, DMAP, pyridine, r.t., 16 h, 87%. **g)** CEP-Cl, 1-methylimidazole, DIPEA, CH_2Cl_2, r.t., 2 h, 86%.

2',3',5'-tris-*O*-(*tert*-butyldimethylsilyl)-1-deazainosine (2)

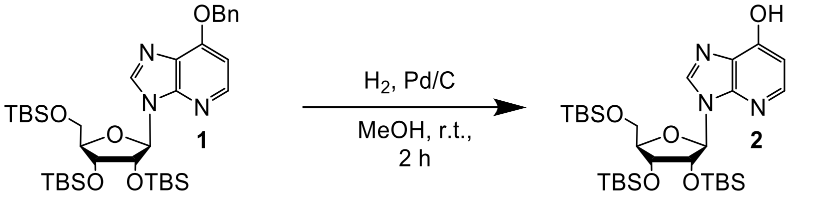

Compound **1** (4.80 g, 6.80 mmol) was dissolved in 20 mL of methanol. Then, 10% palladium on charcoal (Pd/C, 531 mg, 449 µmol) was added, and a septum was attached. Hydrogen was bubbled through the solution for 10 minutes using a balloon and a syringe, and the solution was stirred vigorously under a hydrogen atmosphere for two hours at room temperature. After the reaction, the catalyst was filtered over a celite pad, and the solvent was removed under reduced pressure. The crude compound was purified by silica gel chromatography using 0 to 20% ethyl acetate in cyclohexane as gradient. Yield: 3.20 g of compound **2** as a white foam (77%). TLC: (cyclohexane / ethyl acetate, 85:15): R_f = 0.33. ESI-MS (m/z): $[M+H]^+$ calcd.: 610.3522, found: 610.3513. ^{1}H-NMR: (400 MHz, DMSO-d_6, 25 °C): δ = -0.41 (3H, s, Si-C***H₃***), -0.14 (3H, s, Si-C***H₃***), 0.08 - 0.13 (12H, m, Si-C***H₃***), 0.69 (9H, s, Si-C(C***H₃***)₃)), 0.91 (18H, d, *J* = 9.75, Si-C(C***H₃***)₃)), 3.73 (1H, m, ***H***bC(5')), 4.02 (1H, m, ***H***aC(5')) & ***H***C(4')), 4.33 (1H, m, ***H***C(3')), 4.99 (1H, m, ***H***C(2')), 6.02 (1H, d, *J* = 6.43 Hz, ***H***C(1')), 6.66 (1H, d, *J* = 5.48 Hz, ***H***C(1)), 8.03 (1H, d, *J* = 5.48 Hz, ***H***C(2)), 8.42 (1H, s, ***H***C(8)), 11.20 (1H, s, O***H***). ^{13}C-NMR: (100 MHz, DMSO-d_6, 25 °C): δ = (-4.91) - (-4.68) (-***C***H_3-Si-***C***H_3), 17.47 - 18.00 (Si-C(***C***H_3)$_3$), 25.44 - 26.32 (Si-***C***(CH_3)$_3$), 62.49 (***C***(5')), 72.41 ***C***(3'), 73.88 (***C***(2')), 85.07 (***C***(4')), 86.91 (***C***(1')), 105.62 (***C***(1)), 125.22 (***C***(5)), 141.09 (***C***(8)), 145.29 (***C***(2)), 148.61 (***C***(4)), 156.13 (***C***(6)).

O^6-(*p*-Nitrophenylethyl)-2',3',5'-tris-*O*-(*tert*-butyldimethylsilyl)-1-deazainosine (3)

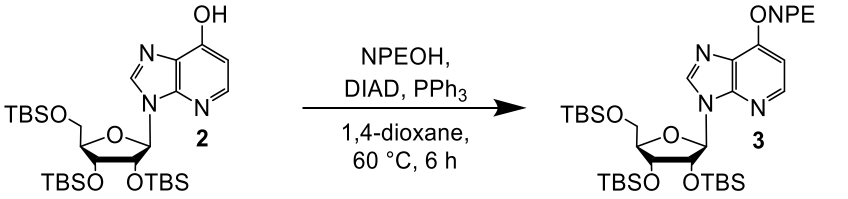

Compound **2** (2.80 g, 4.60 mmol), triphenylphosphine (PPh_3, 4.20 g, 16.1 mmol) and 2-(4-nitrophenyl)-ethanol (NPEOH, 2.70 g, 16.1 mmol) were dissolved in 25 mL of dry 1,4-dioxane and the mixture was stirred for 10 minutes. Then, diisopropyl azodicarboxylate (DIAD, 3.30 g, 3.20 mL, 13.4 mmol) was added over a syringe and the mixture was stirred for 6 hours at 60 °C. Subsequently, the solvent and all volatiles were removed under reduced pressure and the crude product was purified by silica gel chromatography using 0 to 20% ethyl acetate in cyclohexane as gradient. Yield: 3.10 g of compound **3** as a colorless oil (89%). TLC: (ethyl acetate/ cyclohexane 15:85): R_f = 0.36. ESI-MS (m/z): $[M+H]^+$ calcd.: 759.3999, found: 759.3988. ^{1}H-NMR: (400 MHz, $CDCl_3$, 25 °C): δ = -0.28 (3H, s, Si-C***H₃***), -0.07 (3H, s, Si-C***H₃***), 0.10 - 0.14 (12H, m, Si-C***H₃***), 0.77 (9H, s, Si-C(C***H₃***)₃)), 0.94 (18H, d, *J* = 8.33, Si-C(C***H₃***)₃), 3.31 (2H, t, *J* = 6.78 Hz, -OCH_2C***H₂*** (NPE)), 3.79 (1H, m, ***H***bC(5')), 4.03 (1H, m, ***H***aC(5')), 4.13 (1H, m, ***H***C(4')), 4.33 (1H, m, ***H***C(3')), 4.74 (1H, m, ***H***C(2') & -OC***H₂***CH_2 (NPE)), 6.12 (1H, d, *J* = 5.40 Hz, ***H***-C(1')), 6.65 (1H, d, *J* = 5.59 Hz, ***H***C(1)), 7.51 (2H, d, *J* = 8.72 Hz, o-***H***-phenyl (NPE)), 8.19 (3H, m, m-***H***-phenyl (NPE) & ***H***C(2)), 8.24 (1H, s, ***H***C(8)). ^{13}C-NMR: (100 MHz, $CDCl_3$, 25 °C): δ = (-4.97) - (-4.27) (-***C***H_3-Si-***C***H_3), 18.00 - 18.69 (Si-***C***(CH_3)$_3$), 25.84 - 26.25 (Si-C(***C***H_3)$_3$), 35.77 (-OCH_2***C***H_2O-), 62.81 (***C***(5')), 69.50 (-O***C***$H_2$$CH_2$O-), 72.31 (***C***(3')), 75.63 (***C***(2')), 85.54 (***C***(4')), 88.36 (***C***(1')), 103.68 (***C***(1)), 123.92 (m-***C***H-phenyl (NPE)), 125.92 (***C***(5)),

130.14 (o-***C***H-phenyl (NPE)), 141.08 (***C***(8)), 145.91 & 145.94 (***C***(2) & CH_2-***C***(phenyl)NPE), 147.07 (p-C-phenyl(NPE)), 149.15 (***C***(4)), 156.57 (***C***(6)).

O^6-(*p*-Nitrophenylethyl)-1-deazainosine (4)

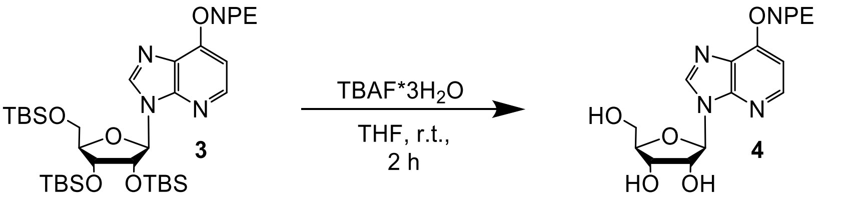

Compound **3** (2.90 g, 3.80 mmol), tetra-*n*-butylammoniumfluoride trihydrate (TBAF * 3 H_2O, 4.80 g, 15.3 mmol) and a few drops of acetic acid were dissolved in 35 mL tetrahydrofuran and stirred at room temperature for two hours. Next, the solvent was removed and the crude product was purified by silica gel chromatography using 0 to 10% methanol in dichloromethane as gradient. Yield: 1.20 g of compound **4** as a white solid (75%). TLC: (methanol / dichloromethane 2:8): R_f = 0.65. ESI-MS (m/z): $[M+H]^+$ calcd.: 417.1405, found: 417.1397 ^{1}H-NMR: (400 MHz, DMSO-d_6, 25 °C): δ = 3.30 (2H, t, *J* = 6.58 Hz, -OCH_2C***H_2*** (NPE)), 3.56 (1H, m, ***H***aC(5')), 3.67 (1H, m, ***H***bC(5')), 4.16 (1H, q, *J* = 4.32 Hz, ***H***C(4')), 4.63 (1H, q, *J* = 5.74 Hz, ***H***C(3')), 4.73 (1H, t, *J* = 6.62 Hz, -OC***H_2***CH_2 (NPE)), 5.18 (1H, d, *J* = 4.77 Hz, ***H***O(3')), 5.35 (1H, q, *J* = 3.84 Hz, ***H***O(5')), 5.43 (1H, d, *J* = 6.21 Hz, ***H***O(2')), 6.00 (1H, d, *J* = 6.02 Hz, ***H***C(1')), 6.91 (1H, d, *J* = 5.67 Hz, ***H***C(1)), 7.66 (2H, d, *J* = 8.77 Hz, o-***H***-phenyl (NPE)), 8.19 (3H, m, m-***H***-phenyl (NPE) & ***H***C(2)), 8.52 (1H, s, ***H***C(8)). ^{13}C-NMR: (100 MHz, DMSO-d_6, 25 °C): δ = 34.61 (-OCH_2***C***H_2O-), 61.56 (***C***(5')), 68.97 (-O***C***H_2CH_2O-), 70.54 (***C***(3')), 73.35 (***C***(2')), 85.65 (***C***(4')), 87.82 (***C***(1')), 103.65 (***C***(1)), 123.41 (m-***C***H-phenyl (NPE)), 125.29 (***C***(5)), 130.34 (o-***C***H-phenyl (NPE)), 141.88 (***C***(8)), 145.44 (***C***(2)), 146.29 (CH_2-***C***(phenyl)NPE), 146.54 (p-C-phenyl(NPE)), 148.46 (***C***(4)), 156.36 (***C***(6)).

O^6-(*p*-Nitrophenylethyl)-2'-***O***-(*tert*-butyldimethylsilyl)-1-deazainosine (5)

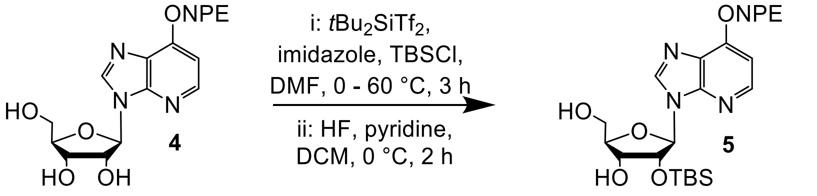

Step i: Compound **4** (1.10 g, 2.64 mmol) was co-evaporated twice with dry pyridine, then dissolved in 5.30 mL of *N*,*N*-dimethylformamide and cooled to 0 °C in an ice bath. Then, di-*tert*-butylsilyl-bis(trifluoro-methansulfonate) (*t*Bu_2SiTf_2, 1.30 g, 948 µL, 2.90 mmol) was added dropwise and stirred for 30 minutes at the same temperature. Imidazole (899 mg, 13.2 mmol) was added and stirring was continued for 30 minutes at room temperature. Next, *tert*-butyldimethylsilylchloride (TBSCl, 478 mg, 3.20 mmol) was added, and the reaction mixture was heated to 60 °C and stirred for two hours. After quenching with methanol, the mixture was concentrated under reduced pressure, dissolved in dichloromethane and transferred to a separatory funnel. Then, the organic layer was washed once with saturated sodium bicarbonate solution and twice with brine, dried over Na_2SO_4 and evaporated to dryness. **Step ii:** The residue of step i was dissolved in 13 mL of dry dichloromethane and cooled to 0 °C. Meanwhile, a solution made of 1.7 mL of pyridine and HF in pyridine (70%, 290 mg, 264 µL, 10.2 mmol) was prepared and added to the dissolved compound and stirred for 2 hours at 0

°C. Afterwards, the mixture was washed successively with water, twice with saturated sodium bicarbonate solution and brine. The resulting organic layer was dried over Na_2SO_4, filtered, concentrated and purified by silica gel chromatography using 0 to 3% methanol in dichloromethane as gradient. Yield: 1.40 g of compound **5** as a white foam (98%). TLC: (5% methanol in dichloromethane): R_f = 0.46. ESI-MS (m/z): $[M+H]^+$ calcd.: 531.2270, found: 531.2263. ^{1}H-NMR: (400 MHz, DMSO-*d*$_6$, 25 °C): δ = -0.26 (3H, s, Si-C***H₃***), -0.15 (3H, m, Si-C***H₃***), 0.70 (9H, s, Si-C(C***H₃***)₃), 3.29 (2H, m, -OCH₂C***H₂*** (NPE)), 3.59 (1H, m, ***H***bC(5')), 3.72 (1H, m, ***H***aC(5')), 4.01 (1H, m, ***H***C(4')), 4.14 (1H, m, ***H***C(3')), 4.74 (1H, m, ***H***C(2') & -OC***H₂***CH₂ (NPE)), 5.06 (1H, d, *J* = 5.05 Hz, ***H***O(3')), 5.49 (1H, m, ***H***O(5')), 6.03 (1H, d, *J* = 6.03 Hz, ***H***C(1')), 6.90 (1H, d, *J* = 5.69 Hz, ***H***C(1)), 7.65 (1H, d, *J* = 8.75 Hz, o-***H***-phenyl (NPE)), 8.18 (3H, m, m-***H***-phenyl (NPE) & ***H***C(2)), 8.54 (1H, s, ***H***C(8)). ^{13}C-NMR: (100 MHz, DMSO-*d*$_6$, 25 °C): δ = -5.42 & -5.06 (-***C***H₃-Si-***C***H₃), 17.75 (Si-***C***(CH₃)₃), 25.50 (Si-C(***C***H₃)₃), 34.60 (-OCH₂***C***H₂O-), 61.45 (***C***(5')), 68.94 (-O***C***H₂CH₂O-), 70.53 (***C***(3')), 75.07 (***C***(2')), 86.03 (***C***(4')), 88.05 (***C***(1')), 103.60 (***C***(1)), 123.38 (m-***C***H-phenyl (NPE)), 125.39 (***C***(5)), 130.35 (o-***C***H-phenyl (NPE)), 141.88 (***C***(8)), 145.44 (CH₂-***C***(phenyl)NPE), 146.29 (p-C-phenyl(NPE)), 146.48 (***C***(4)), 148.23 (***C***(4)), 156.40 (***C***(6)).

5'-*O*-(4,4'-Dimethoxytrityl)-*O*^{6}-(*p*-nitrophenylethyl)-2'-*O*-(*tert*-butyldimethylsilyl)-1-deazainosine (6)

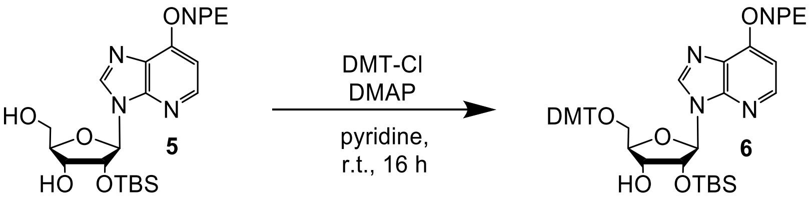

Compound **5** (1.40 g, 2.60 mmol) and 4-dimethylaminopyridine (DMAP, 63.5 mg, 0.50 mmol) were co-evaporated three times with dry pyridine and finally dissolved in 28 mL of dry pyridine. Then, 4,4'-dimethoxytrityl chloride (DMTCl, 1.20 g, 3.40 mmol) was added in two portions over the course of one hour. With each addition DMTCl, the reaction solution was heated to 35 °C for one minute. After the complete addition of DMTCl, the mixture was stirred overnight at room temperature (16 hours). Next, the reaction was quenched with 2.0 mL of methanol, and the mixture was evaporated to dryness. The oily residue was dissolved in dichloromethane and successively washed with 5% citric acid solution, saturated sodium bicarbonate solution and brine. The organic layer was dried over Na_2SO_4, filtered, concentrated and purified by silica gel chromatography using 0 to 30% ethyl acetate in cyclohexane as gradient. Yield: 1.90 g of compound **6** as a slightly yellow foam (87%). TLC: (cyclohexane / ethyl acetate, 6:4): R_f = 0.43. ESI-MS (m/z): $[M+H]^+$ calcd.: 833.3576, found: 833.3558. ^{1}H-NMR: (400 MHz, DMSO-*d*$_6$, 25 °C): δ = -0.19 (3H, s, Si-C***H₃***), -0.07 (3H, m, Si-C***H₃***), 0.73 (9H, s, Si-C(C***H₃***)₃)), 3.26 (4H, m, ***H₂***-C(5')) & -OCH₂C***H₂*** (NPE)), 3.72 (6H, s, 2xO-C***H₃***), 4.10 (1H, q, *J* = 4.37 Hz, ***H***C(4')), 4.22 (1H, q, *J* = 5.13 Hz, ***H***C(3')), 4.71 (1H, t, *J* = 6.64, -OC***H₂***CH₂ (NPE)), 4.88 (1H, t, *J* = 5.10 Hz, ***H***O(3')), 5.10 (1H, d, *J* = 5.92 Hz***,*** ***H***C(2')), 6.05 (1H, d, *J* = 5.11 Hz, ***H***C(1')), 6.82 – 7.41 (13H, ***H***-C(DMT)), 6.89 (1H, d, *J* = 5.65 Hz, ***H***C(1)), 7.65 (2H, d, *J* = 8.73 Hz, o-***H***-phenyl (NPE)), 8.12 (1H, d, *J* = 5.58 Hz, ***H***C(2)), 8.18 (2H, d, *J* = 8.74 Hz, m-***H***-phenyl (NPE), 8.40 (1H, s, ***H***-C(8)). ^{13}C-NMR: (100 MHz, DMSO-*d*$_6$, 25 °C): δ = -5.31 & -4.90 (-***C***H₃-Si-***C***H₃), 17.79 (Si-***C***(CH₃)₃), 25.52 (Si-C(***C***H₃)₃), 34.61 (-OCH₂***C***H₂O-), 54.98 (2xO***C***H₃), 63.50 (***C***(5')), 68.88 (-O***C***H₂CH₂O-), 70.28 (***C***(3')), 74.68 (***C***(2')), 83.29 (***C***(4')), 85.50 (***C***-quart. DMT), 87.89 (***C***(1')), 103.59 (***C***(1)), 113.12 - 135.47 (H***C***-DMT), 123.37 (m-***C***H-phenyl (NPE)), 126.63 ***C***(5), 130.33 (o-***C***H-phenyl (NPE)), 141.57 ***C***(8), 144.90(CH₂-***C***-phenyl(NPE)), 145.67 (p-C-phenyl(NPE)), 148.44 (***C***(4)), 156.15 (***C***(2)), 158.03 (***C***(6)).

5'-*O*-(4,4'-Dimethoxytrityl)-O^6-(*p*-nitrophenylethyl)-2'-*O*-(*tert*-butyldimethylsilyl)-1-deazainosine 3'-*O*-2-cyanoethyl-*N,N*-diisopropylphosphoramidite (7)

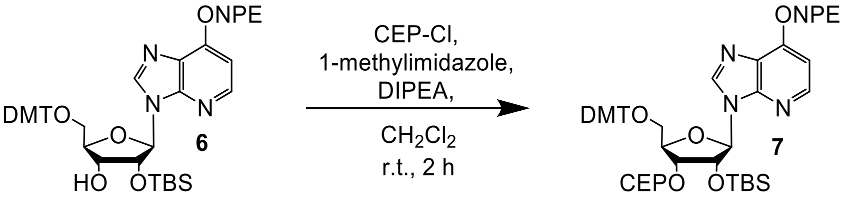

Compound **6** (1.70 g, 2.00 mmol) was dissolved in 1.8 mL of dry dichloromethane. Then, diisopropyl-ethylamine (DIPEA, 1.90 g, 2.60 mL, 15.0 mmol) and 1-methylimidazole (81.8 mg, 79.4 µL, 1.00 mmol) were added. Next, 2-cyanoethyl-*N,N*-diisopropylchlorophosphoramidite (Cep-Cl, 1.21 g, 1.14 mL, 5.10 mmol) was added, and the solution was stirred for 2 hours at room temperature. Thin layer chromatography revealed complete consumption of the starting material. Then, the solvent and all volatiles were removed under reduced pressure, and the crude compound was purified by silica gel chromatography using 10 to 30% ethyl acetate in cyclohexane as gradient. Yield: 1.8 g of compound **7** as a white foam (86%). TLC: (toluene / ethyl acetate, 6:4): R_f = 0.76 & 0.65. ESI-MS (m/z): $[M+H]^+$ calcd.: 1033.4655, found: 1033.4630. ^{1}H-NMR: (400 MHz, $CDCl_3$, 25 °C): δ = -0.25 (3H, s, Si-C***H₃***), -0.06 (3H, d, *J* = 18.66 Hz, Si-C***H₃***), 0.75 (9H, s, Si-C(C***H₃***)₃), 1.03-1.19 (12H, m, ((C***H₃***)₂-CH)₂-N), 2.26 -2.67 (2H, m, OCH₂C***H₂***CN), 3.24-3.34 (3H, m, ***H***aC(5') & -OCH₂C***H₂*** (NPE), 3.53-3.67 (4H, m, ((CH₃)₂-C***H***)₂-N & ***H***bC(5') & C***H₂***(b)-O-P)), 3.77 (6H, s, 2xO-C***H₃***), 3.84-4.01 (1H, m, C***H₂***(a)-O-P), 4.33 - 4.42 (2H, m, ***H***-C(4') & ***H***C(3')), 4.73 (2H, m, -OC***H₂***CH₂ (NPE)), 5.15 (1H, m, ***H***C(2')), 6.05 - 6.13 (1H, dxd, *J* = 10.76 Hz, ***H***C(1')), 6.64 (***H***C(1)), 6.78-7.52 (15H, m, C***H***-arom.- DMT & o-***H***-phenyl (NPE), 8.08-8.19 (4H, m, o-***H***-phenyl (NPE), ***H***C(8)) & ***H***C(2)). ^{31}P-NMR: (162 MHz, $CDCl_3$, 25 °C)= 150.86 & 148.92 ppm.

^{1}H-NMR (400 MHz, $CDCl_3$, 25 °C) of compound **1**

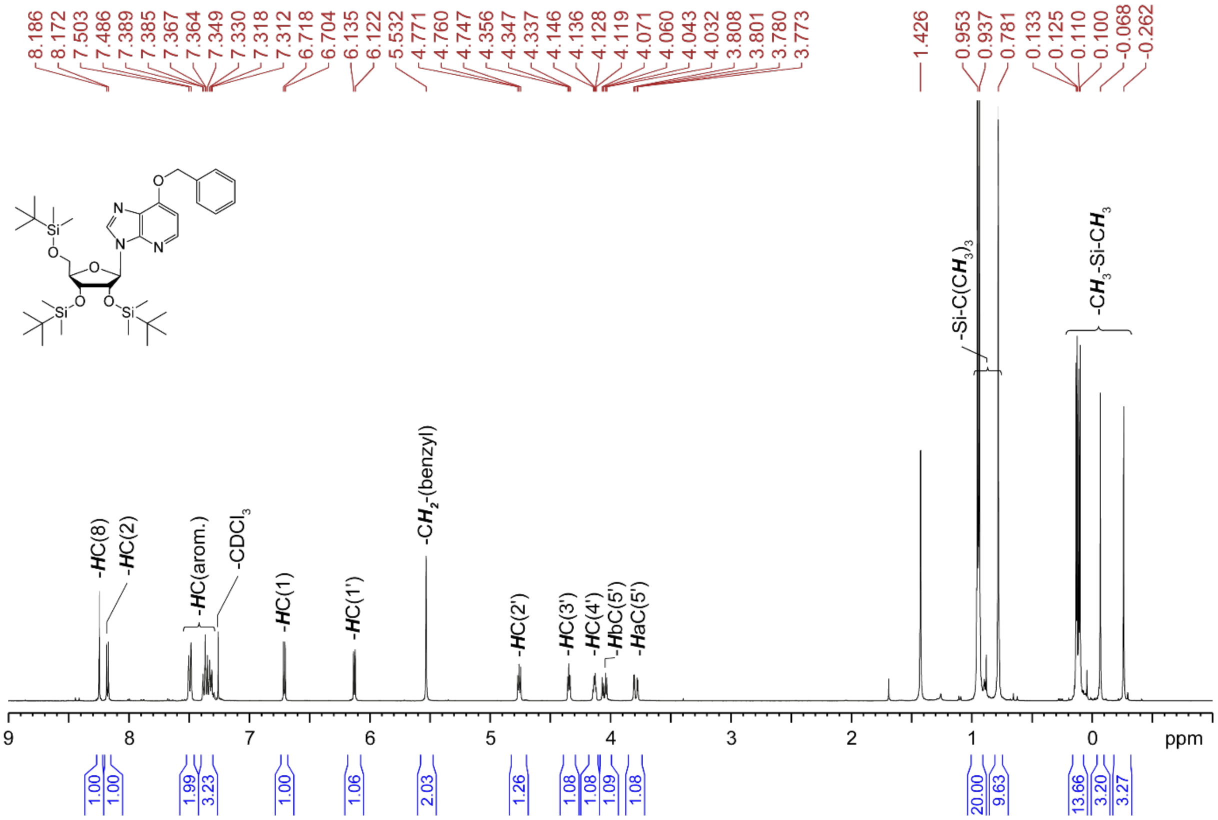

^{13}C-NMR (100 MHz, $CDCl_3$, 25 °C) of compound **1**

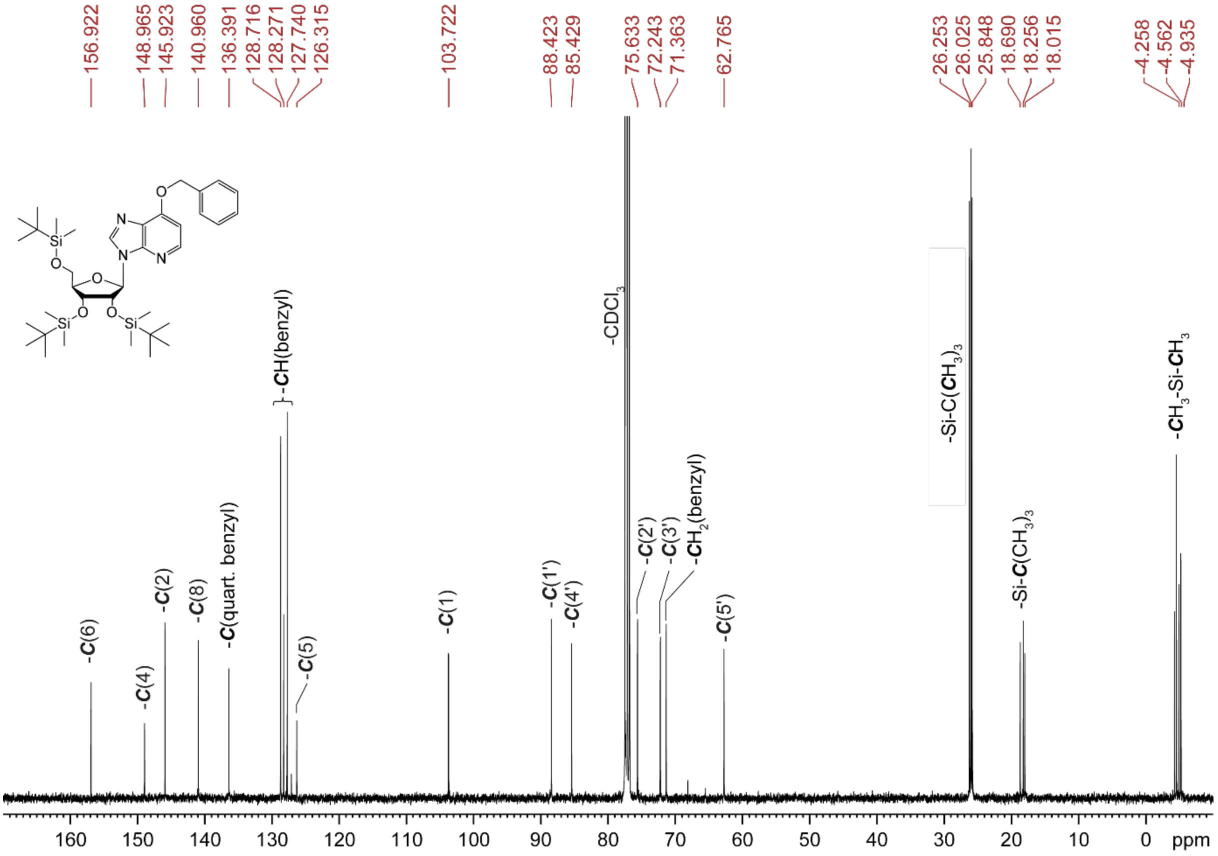

^{1}H-NMR (400 MHz, DMSO-d_6, 25 °C) of compound **2**

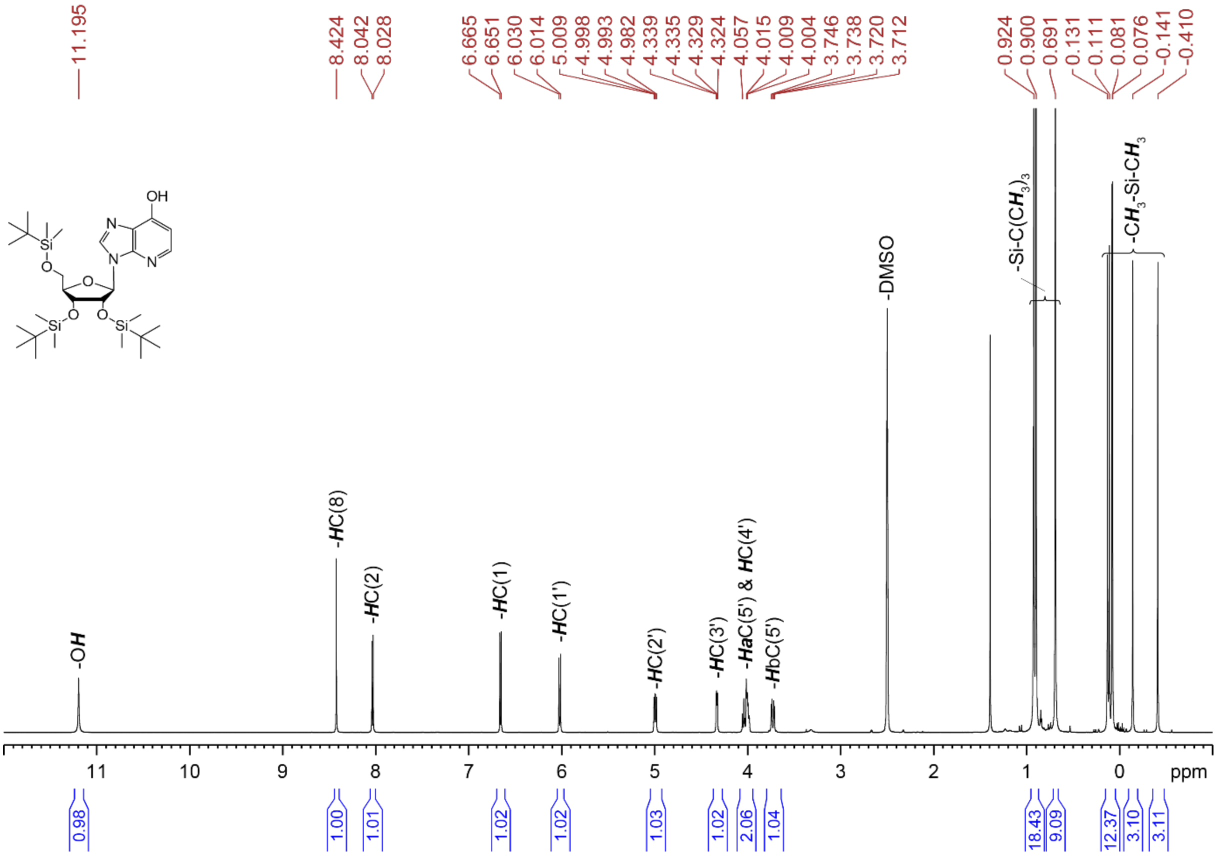

^{13}C-NMR (100 MHz, DMSO-d_6, 25 °C) of compound **2**

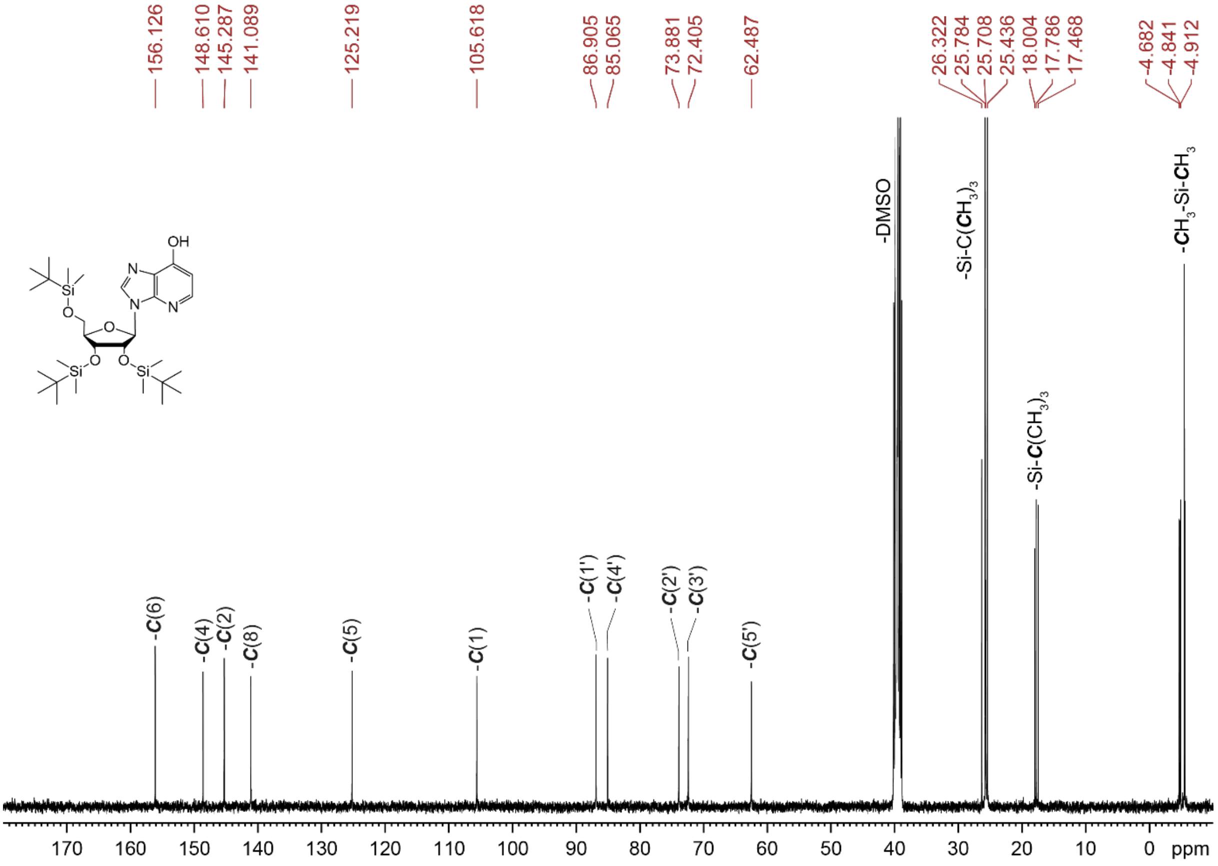

^{1}H-NMR (400 MHz, $CDCl_3$, 25 °C) of compound **3**

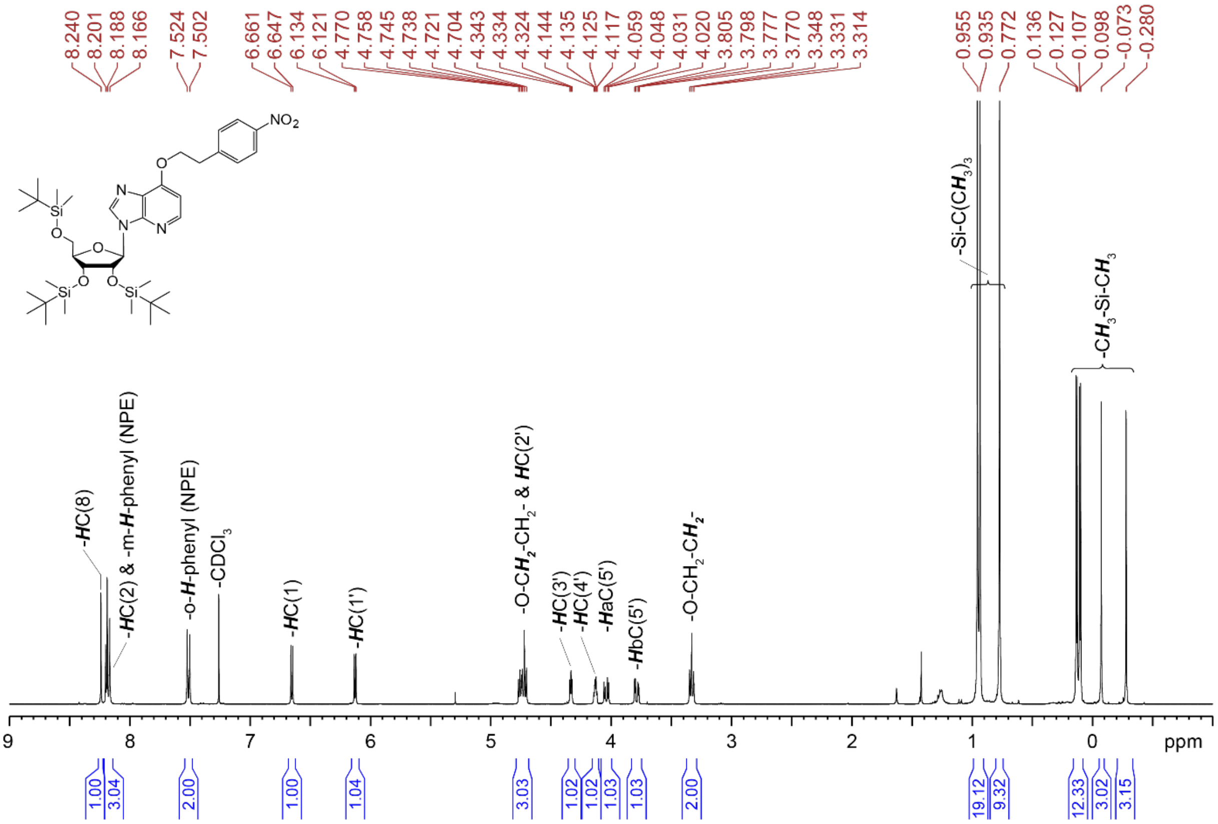

^{13}C-NMR (100 MHz, $CDCl_3$, 25 °C) of compound **3**

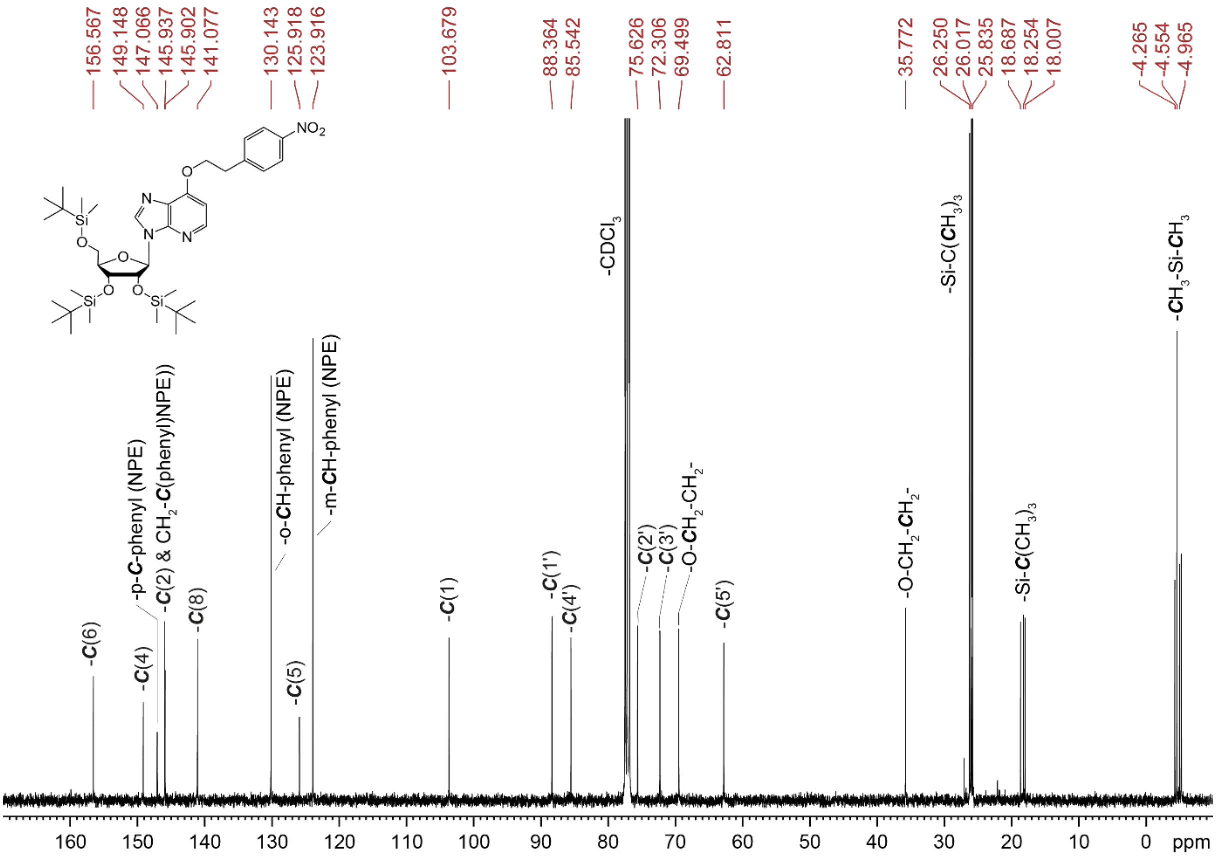

^{1}H-NMR (400 MHz, DMSO-d_6, 25 °C) of compound **4**

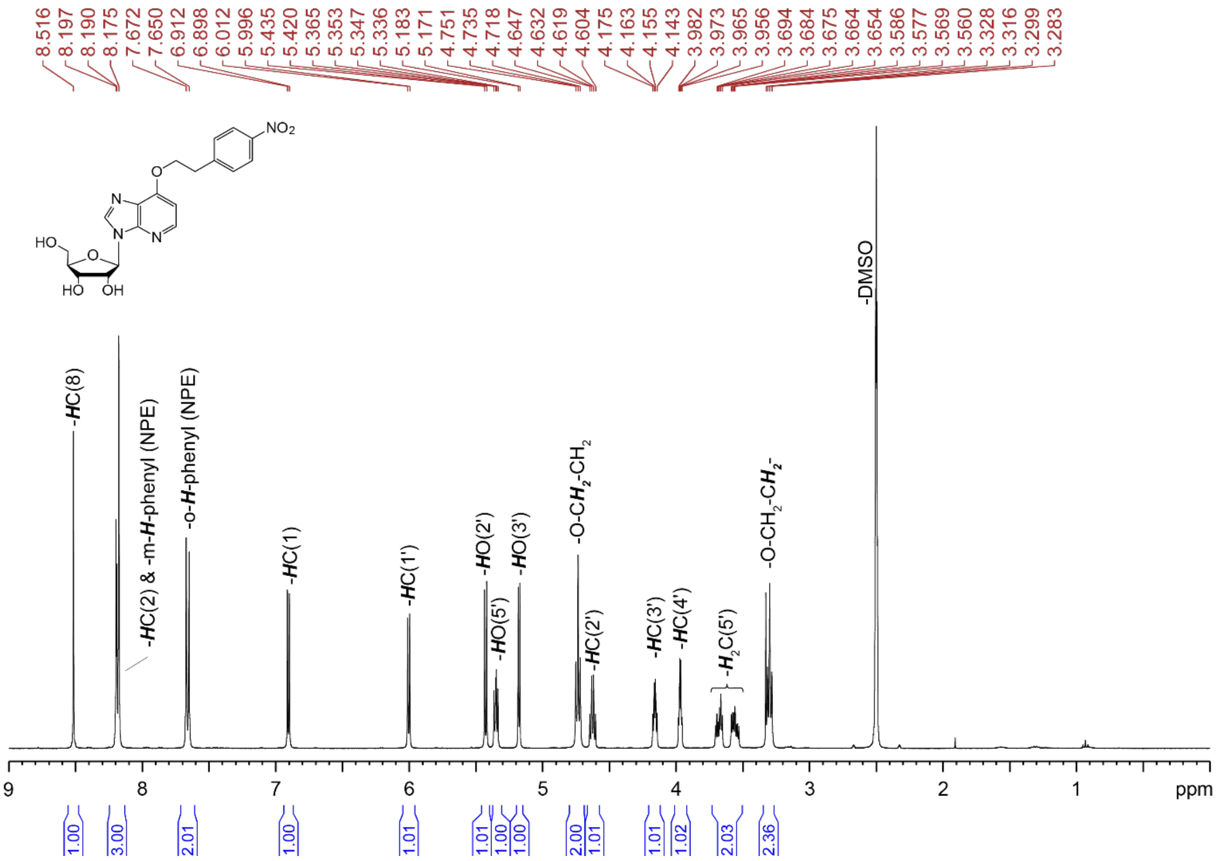

^{13}C-NMR (100 MHz, DMSO-d_6, 25 °C) of compound **4**

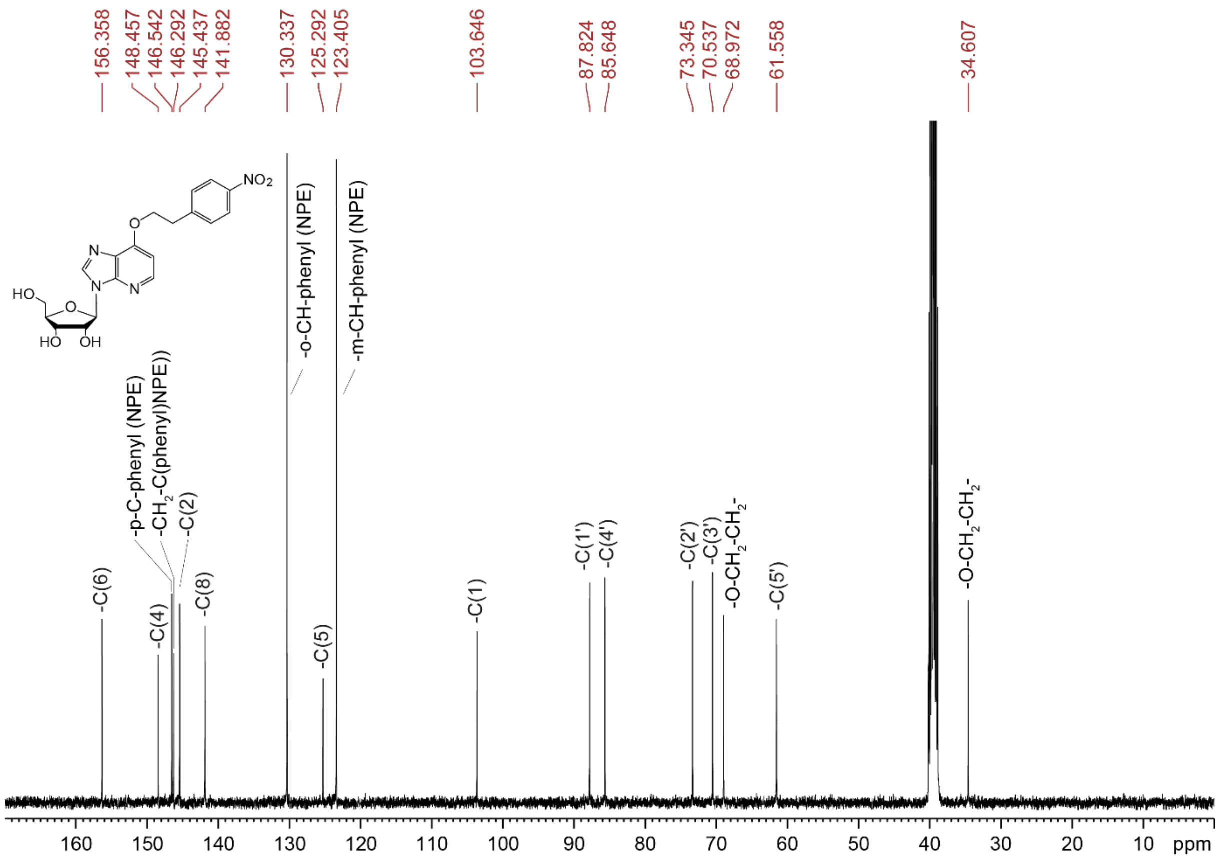

^{1}H-NMR (400 MHz, DMSO-d_6, 25 °C) of compound **5**

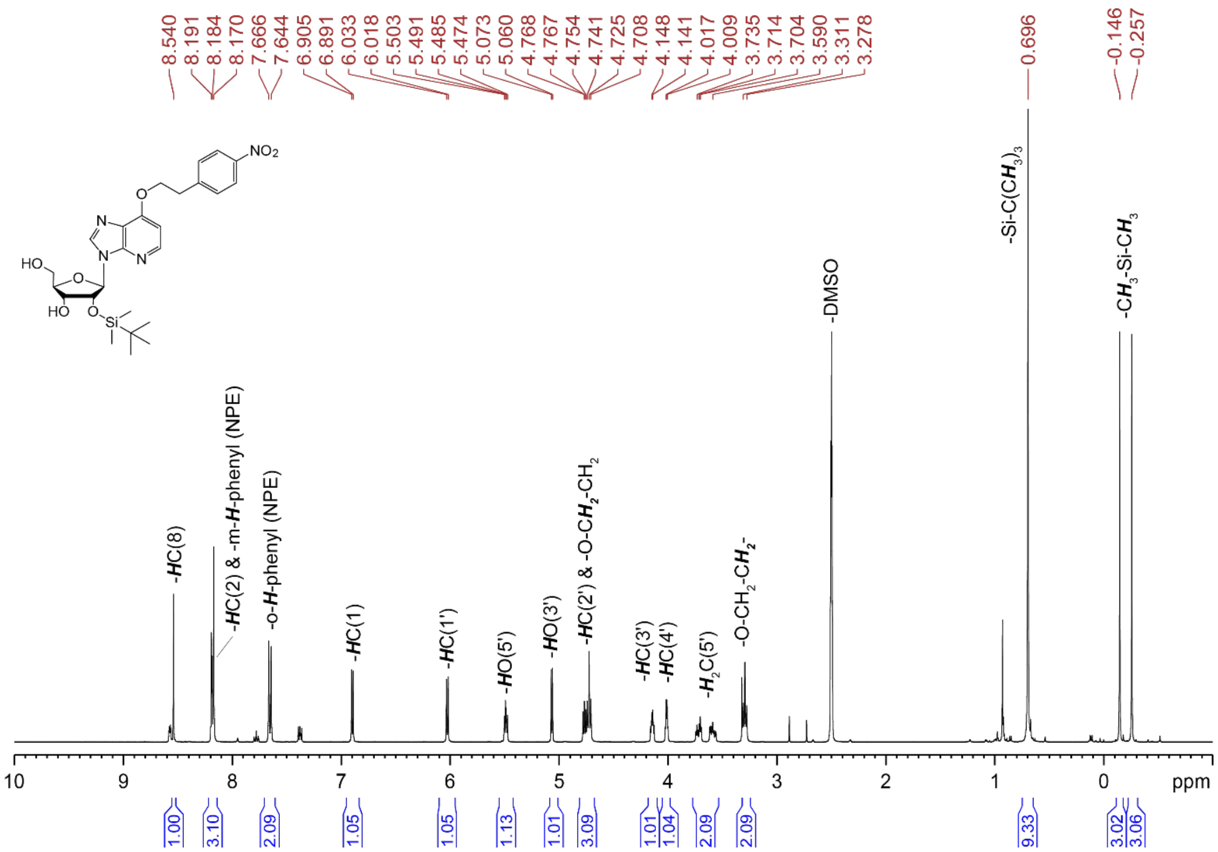

^{13}C-NMR (100 MHz, DMSO-d_6, 25 °C) of compound **5**

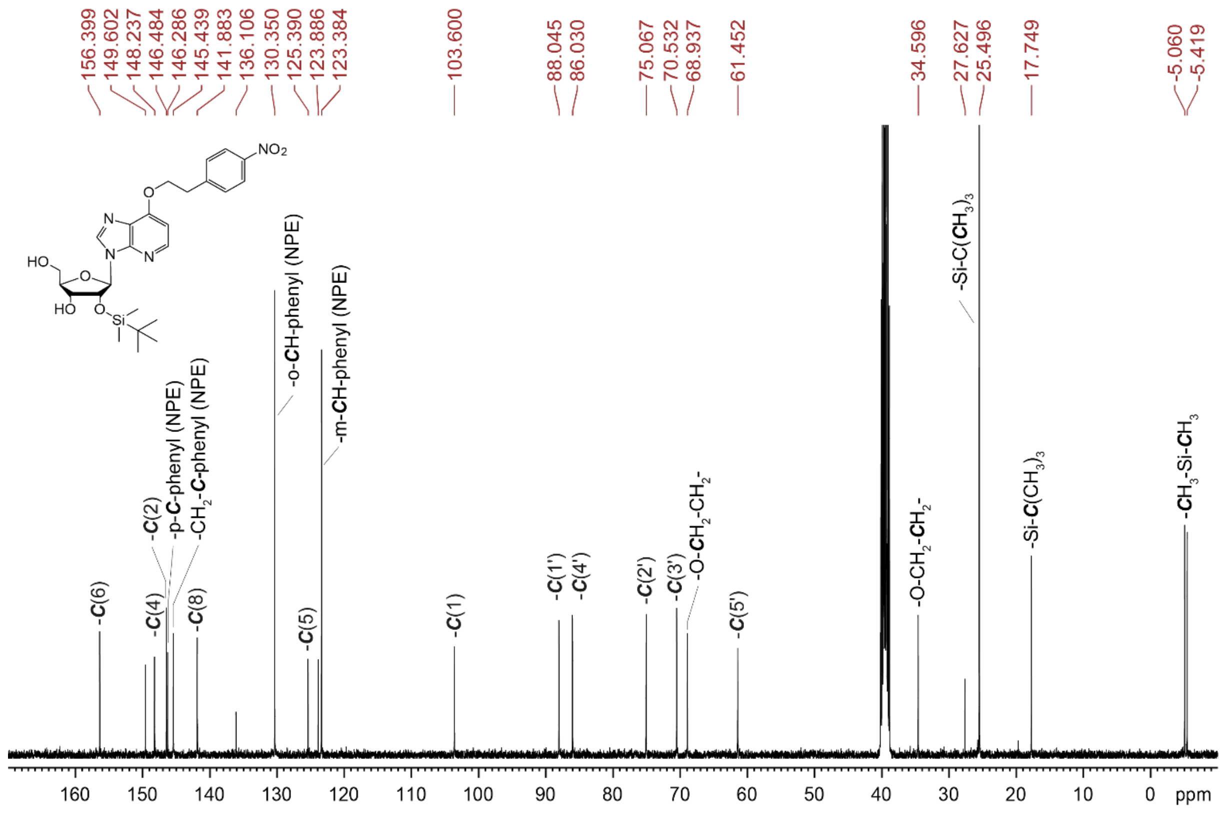

^{1}H-NMR (400 MHz, DMSO-d_6, 25 °C) of compound **6**

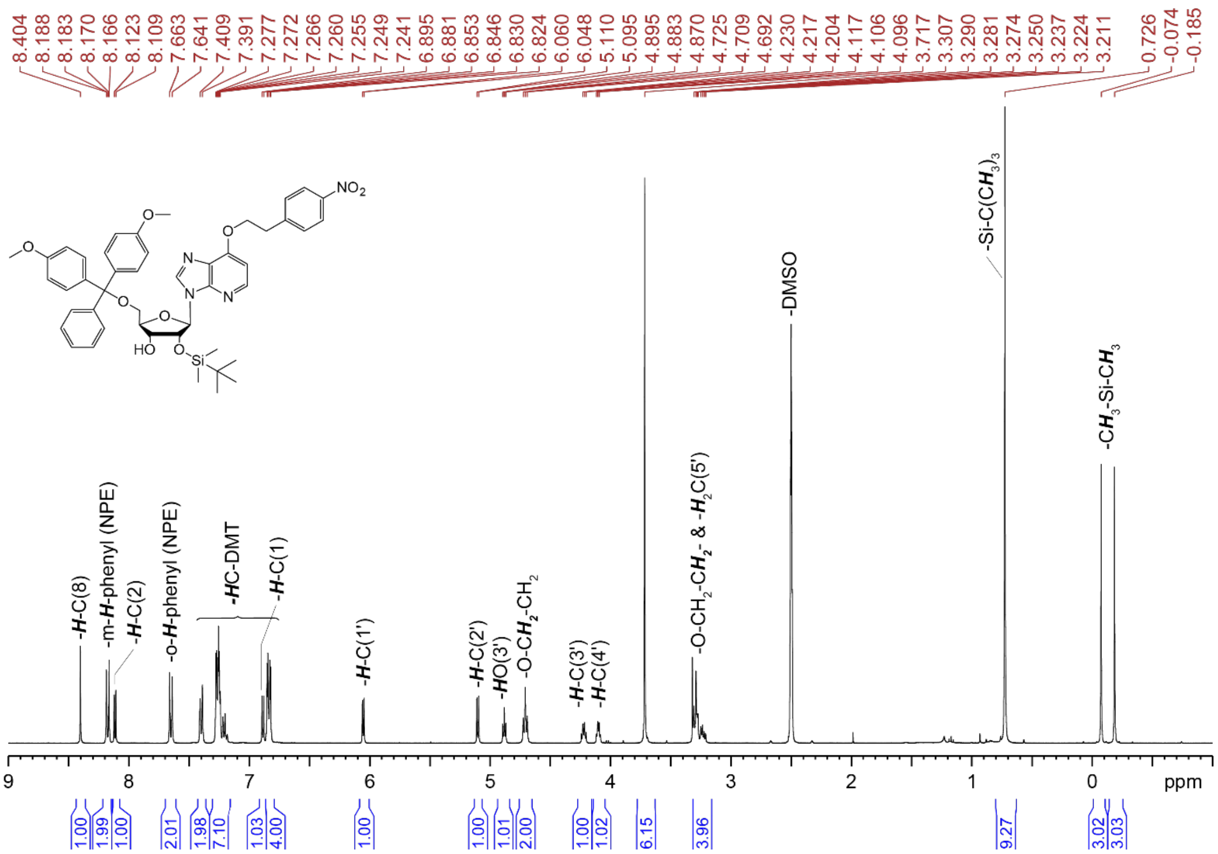

^{13}C-NMR (100 MHz, DMSO-d_6, 25 °C) of compound **6**

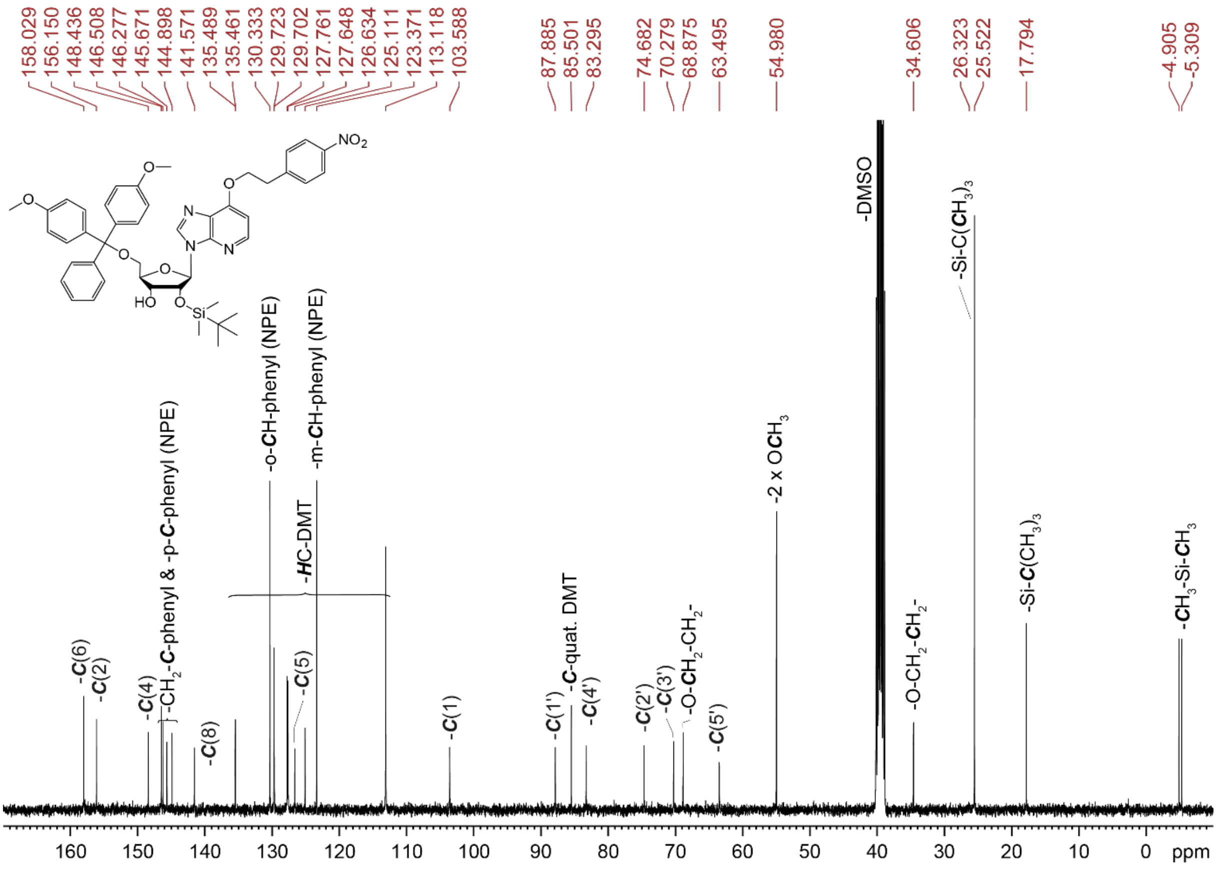

^{1}H-NMR (400 MHz, $CDCl_3$, 25 °C) of compound **7**

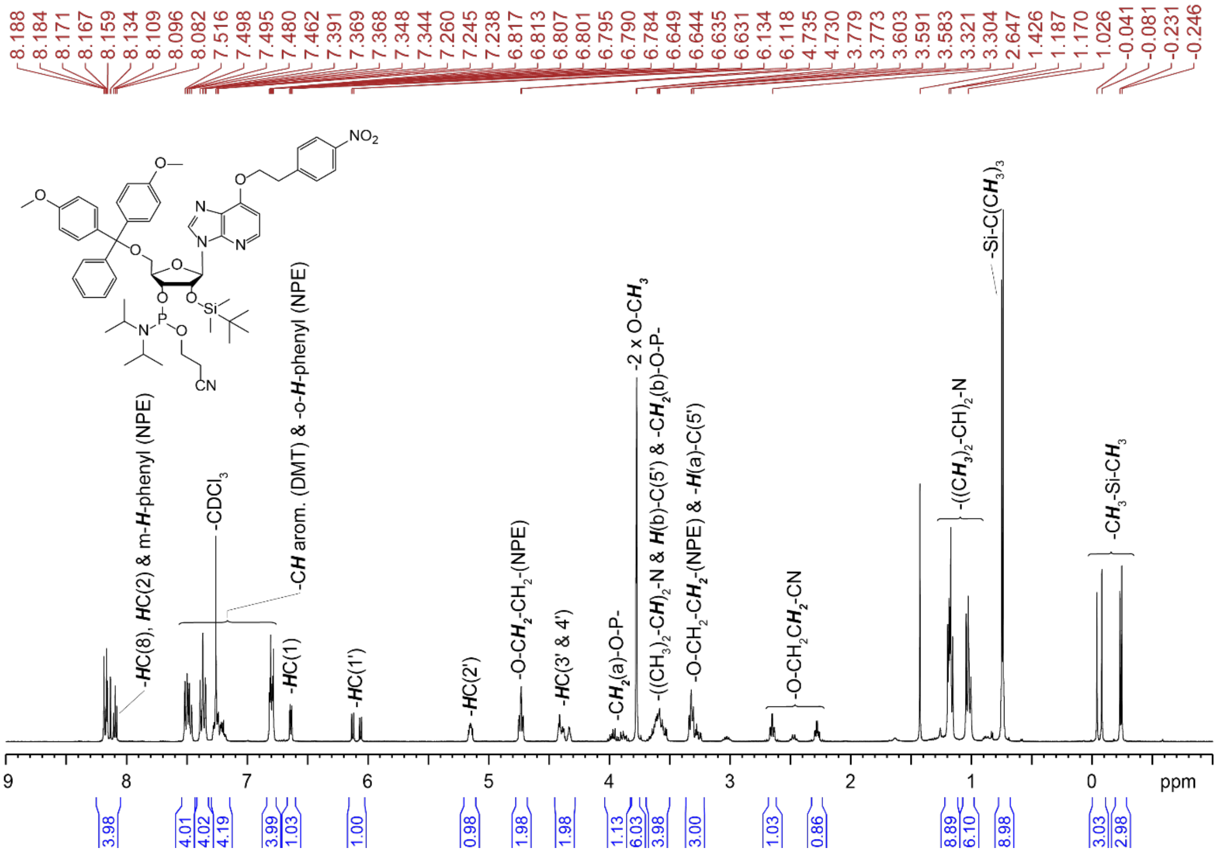

^{31}P-NMR (162 MHz, DMSO-d_6, 25 °C) of compound **7**

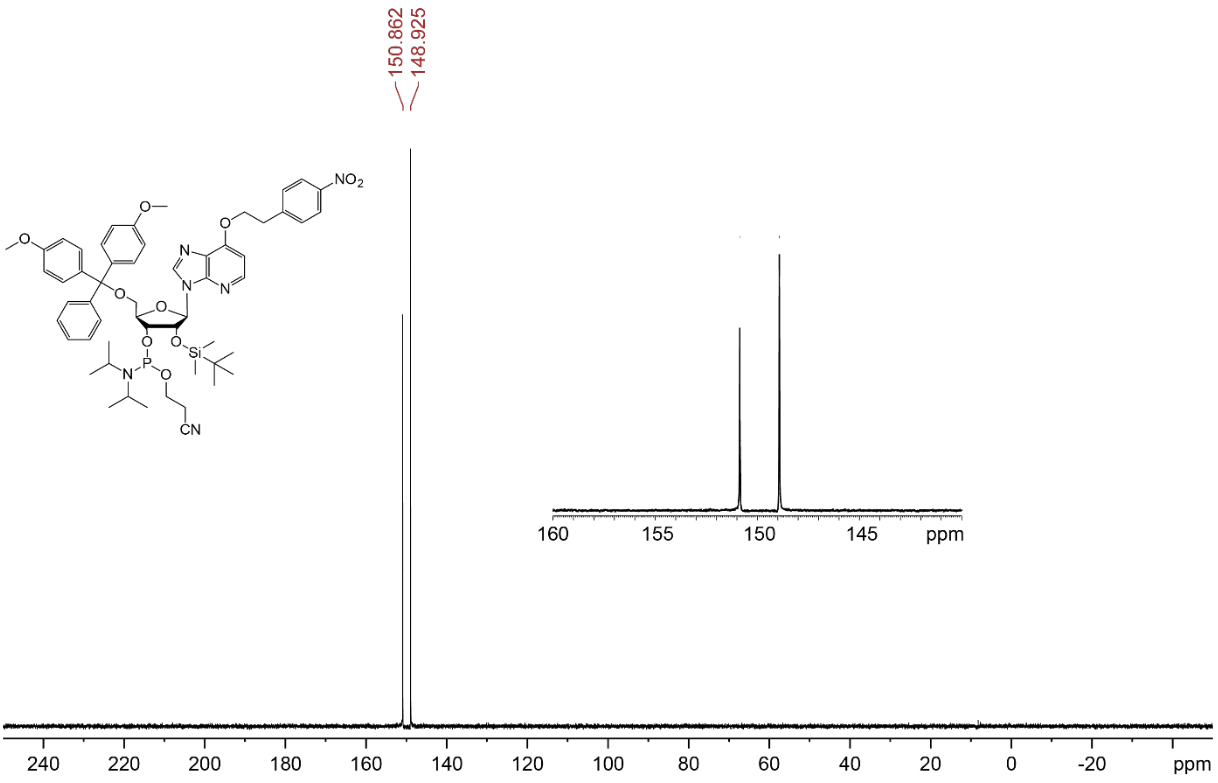

$^{1}H,^{13}C$-HMBC NMR (400 MHz, DMSO-d_6, 25 °C) of c^{1}I nucleoside.

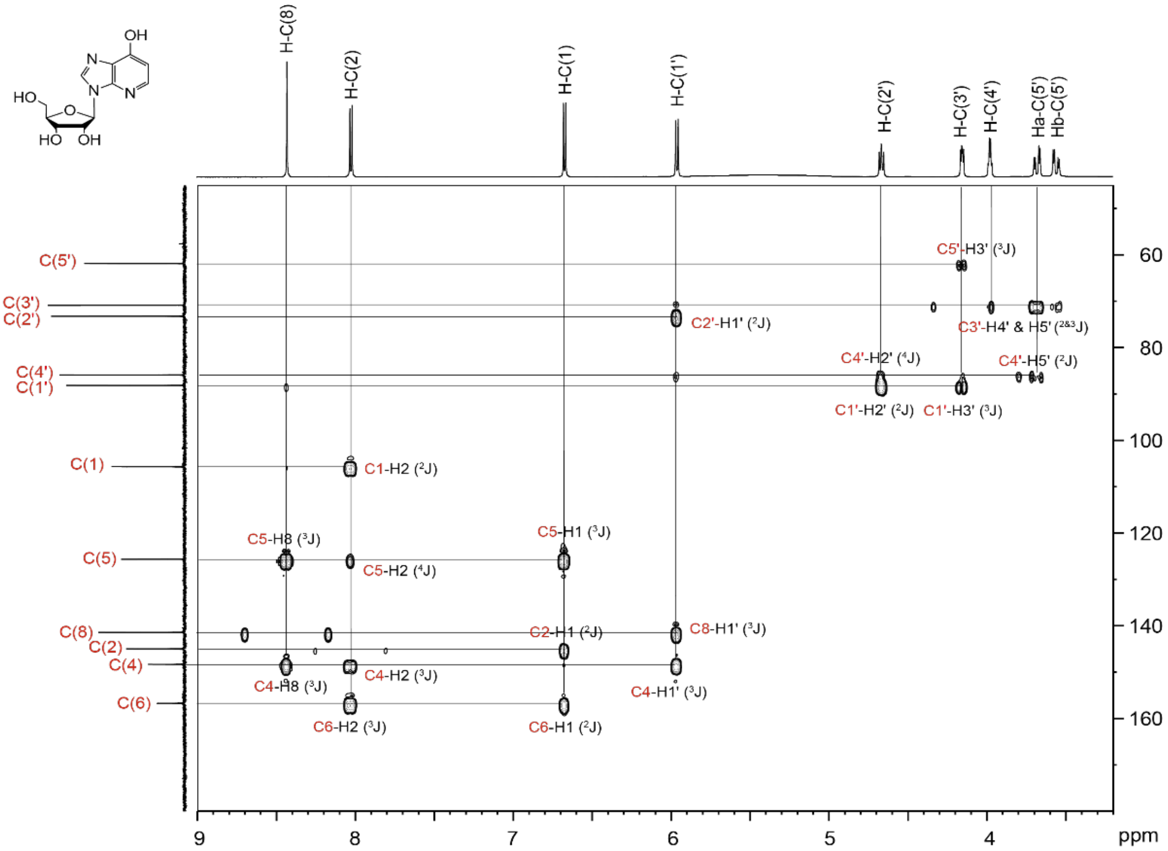

$^{1}H,^{13}C$-HMBC (400 MHz, sodium phosphate buffer: 15 mM Na_2HPO_4, 25 mM NaCl, pH 6.5, 10% D_2O in H_2O, 25 °C) of c^{1}I nucleoside.

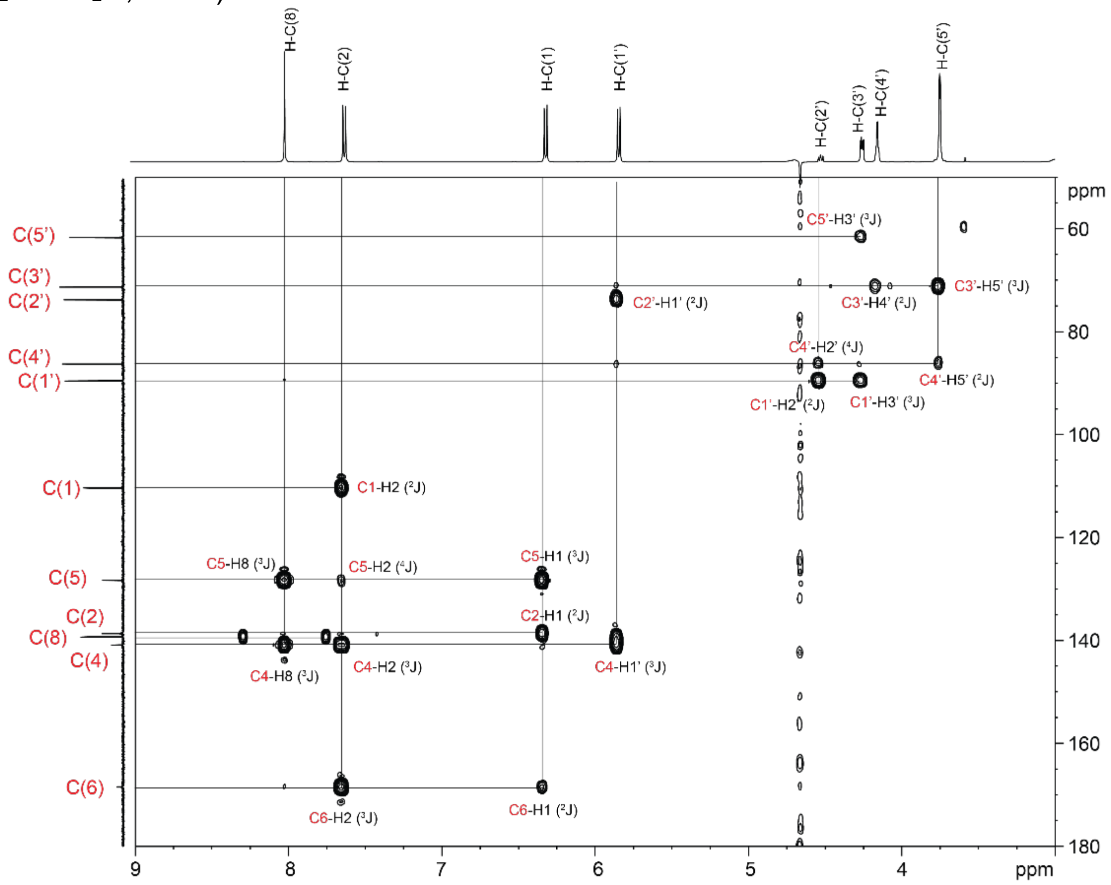

1.3 Solid-phase RNA synthesis

Standard phosphoramidite chemistry was used for RNA strand elongation and 1-deazainosine incorporation. *N*-acetylated 2′-*O*-[(triisopropylsilyl)oxy]methyl (TOM) and *N*-acetylated 2′-*O*-tertbutyldimethylsilyl (TBDMS) nucleoside phosphoramidites, along with 2′-O-TBDMS 1000 Å CPG solid support were purchased from Chemgenes. All oligonucleotides were synthesized on an ABI 392 Nucleic Acid Synthesizer using standard protocols. Detritylation was performed for 90 seconds using dichloroacetic acid/1,2-dichloroethane (4:96). Coupling was carried out for 5 minutes with phosphoramidites (100 mM, 200 µL in acetonitrile) and benzylthiotetrazole (300 mM, 500 µL in acetonitrile). The capping step (2 × 25 seconds) involved Cap A and Cap B (1:1 v/v), where Cap A consisted of *N,N*-dimethylaminopyridine in acetonitrile (491 mM), and Cap B contained acetic anhydride, sym-collidine, and acetonitrile (10:15:25 v/v/v). Oxidation was performed for 60 seconds using a 20 mM iodine solution in tetrahydrofuran/pyridine/ H_2O (35:10:5 v/v/v). The phosphoramidites were prepared at a final concentration of 0.1 M and dried overnight over activated 3 Å molecular sieves.

1.4 Deprotection and purification of RNA

To deprotect unmodified and c^1I-modified RNA, the solid support was treated with aqueous methylamine (40%, 0.75 mL) and aqueous ammonia (28%, 0.75 mL) at 65 °C for 20 minutes. After the reaction, the supernatant was removed, and the solid support was washed twice with a 1:1 mixture of tetrahydrofuran and H_2O (0.5 mL). The combined supernatant and wash fractions were evaporated to dryness.

The resulting residue was dissolved in a 1.0 M solution of tetra-n-butylammonium fluoride in tetrahydrofuran (1.5 mL), and the solution incubated at 37 °C for 14 hours to remove the 2'-O-silyl protecting groups. The reaction was quenched by adding 1.5 mL of 1.0 M aqueous tetraethylammonium acetate solution (pH 7.4). Tetrahydrofuran was then removed under reduced pressure, and the residue was desalted using size-exclusion chromatography with a (column: GE Healthcare HiPrep™ 26/10, Sephadex G25), eluting with H_2O. The collected fractions were evaporated, and the RNA was dissolved in 1 mL of water. Crude RNA purification was performed via anion-exchange chromatography using a GE Healthcare Äkta Explorer HPLC system equipped with a semipreparative Dionex DNAPac™ PA-100 column (9 mm × 250 mm) at 80 °C, with a pressure range of 1.7 to 2.0 MPa and a flow rate of 1 mL/min. The mobile phase consisted of eluent A (25 mM Tris·HCl, 0.01 M $NaClO_4$, 20% acetonitrile, pH 8.0) and eluent B (25 mM Tris·HCl, 0.6 M $NaClO_4$, 20% acetonitrile, pH 8.0).

RNA-containing fractions were diluted with 0.1 M triethylammonium bicarbonate solution, loaded onto a C18 SepPak Plus® cartridge (Waters/Millipore), washed with H_2O, and eluted with a mixture of acetonitrile/H_2O (1:1). Both the crude and the purified RNA were analyzed by anion exchange chromatography using a ThermoFisher Vanquish HPLC system with a Dionex DNAPac™ PA-100 column (4 mm × 250 mm), at 80 °C with a flow rate of 1 mL/min, unless otherwise specified. For RNA sequences ≤15 nucleotides, a gradient of 0 to 40% eluent B over 30 minutes was applied. For RNA sequences >15 nucleotides, a gradient of 0 to 60% eluent B over 45 minutes was applied. UV absorbance at 260 nm was recorded to generate HPLC traces, and RNA quantification was conducted using an Implen P300 Nanophotometer.

1.5 Mass spectrometric analysis of RNA

All experiments were performed using either a Finnigan LCQ Advantage MAX ion trap instrument with a spray voltage of 4.0 kV in negative mode or a ThermoFisher Orbitrap Exploris™ 240 mass spectrometer with a spray voltage of 3.0 kV in negative mode. The mass

spectrometer was connected to a ThermoFisher Ultimate 3000 HPLC system. RNA sequences were analyzed using the following LC conditions: sample (200 pmol of RNA dissolved in 30 µL solution of 20 mm EDTA; average injection volume: 30 µL), column (Waters XBridge, Oligo BEH C18 2.5 µm; 2.1×50 mm) at 30 °C; and flow rate: 0.1 mL min^{-1}; eluent A: Et_3N (8.6 mM), 1,1,1,3,3,3-hexafluoroisopropanol (100 mM) in H_2O (pH 8.0); eluent B: MeOH; gradient: 0 to 100 % B in A within 20 min; UV detection at 254 nm.

1.6 UV melting experiments of RNA

For the UV melting curve analysis the pure RNA samples were lyophilized as sodium salts and dissolved in a phosphate buffer (10 mM Na_2HPO_4, 100 mM KCl, pH = 7.0). The volume of buffer solution depended on RNA concentration: 800 µL for concentrations of 2, 5, 8 and 12 µM, and 330 µL for concentrations of 35 and 90 µM. Thermal melting curves were recorded using a Varian Cary 100 spectrometer equipped with a Peltier temperature controller. Five different concentrations were monitored for duplex RNA (2, 5, 12, 35, and 90 µM), and two different concentrations were monitored for hairpin systems (2 and 8 µM). Three heating and three cooling ramps, with a rate of 0.5 °C/min were measured within a temperature range of 0 to 85 °C, and were monitored at 250 and 260 nm.

1.7 NMR sample preparation, NMR experiments and chemical shift assignment and structure calculation

NMR samples were prepared in 15 mM sodium phosphate (pH 6.5), 25 mM NaCl, using 90% H_2O/10% D_2O or 100% D_2O, with data acquired at 278 to 298 K and a field strength of 600 or 700 MHz. An initial 1D ^1H NMR spectrum with water suppression (Bruker pulse program zgesgp) was collected on a sample of 0.4 mM hairpin and in 90% H_2O/10% D_2O to confirm proper RNA folding, followed by a 1D ^1H spectrum with selective excitation of the imino protons (IBS_1D_iminos). Then, 2D $^1H,^1H$-TOCSY (dipsi2esfbgpph_ck, 90 ms mixing time) and 2D $^1H,^1H$ NOESY (noesygpphjrrs, 150 ms mixing time) experiments were recorded. For the assignment of non-exchangeable resonances, a 2D $^1H,^1H$ NOESY (noesyesgpph, 150 ms mixing time), two 2D $^1H,^{13}C$-HSQC spectra (hsqcetfpgpsi2, one with focus on aromatic resonances (o2p 140 ppm, ^{13}C spectral width 50 ppm and $^1J_{CH}$ 185 Hz; one with focus on sugar resonances (o2p 90 ppm, ^{13}C spectral width 50 ppm, $^1J_{CH}$ 170 Hz)) and a 2D $^1H,^1H$-TOCSY (dipsi2gpph19, 80 ms mixing time) in 100% D_2O (15 mM sodium phosphate, pH 6.5, 25 mM NaCl) were measured. Due to the similar NMR spectra observed for the wild-type and 1-deazainosine containing hairpin it was possible to directly transfer most of the already determined chemical shift assignments. For the purpose of structure determination, it was necessary to determine the chemical shift assignments at 298 K of the 2D $^1H,^1H$-NOESY spectra (noesyesgpph, 150 ms mixing time) and the ^1H and ^{13}C sugar resonances are based on 2D $^1H,^{13}C$-HSQC spectra (hsqcetgpsp.3, with a ^{13}C spectral width of 30 ppm centered on 75 ppm). For the resonance assignment the software package *Poky* was used.[2]

For the structure calculation, the program Xplor-NIH[3] was used. First an ensemble of structures was calculated using distance restraints involving exchangeable proton resonances from the 9/1 H_2O/D_2O NOESY at 278 K.

No strong H8-H1' purine nucleotide NOE cross peak was found in the D_2O NOESY, thus all A/G residues were restrained to the anti-conformation. Based on the intensities of the H2′-H1′ TOCSY cross peaks, sugar puckers were determined. For most residues in the stem, low intensity or no H1'-H2' cross peaks were observed, in-line with a C3'-endo sugar pucker. The sugar puckers of residues G15 and A9 were kept unrestrained. The final structure ensemble

was obtained as 10 lowest energy structures from a total of 100 calculated models. Complete refinement statistics for this step are presented in Supporting Table S3. Additionally, to further refine the structure residual dipolar couplings (RDCs) were measured and used in the refinement step. For this purpose, 2D $^{1}H,^{13}C$-HSQC spectra at 298K of an RNA sample containing 1.4 mM RNA (15 mM sodium phosphate, 25 mM NaCl, pH 6.5, D_2O) were measured without and with Pf1 phages (15 Hz splitting in D_2O resonance). The RDCs were determined by running the HSQC experiments without ^{13}C decoupling during the acquisition.

1.8 Ribozyme cleavage assays

The ribozyme and substrate strands (5.5 nmol each) were lyophilized as triethylammonium salts and dissolved in H_2O (33 µL). The dissolved RNA strands were heated to 90 °C for 2 minutes and subsequently cooled to room temperature. 2 µL of this solution were used for HPLC analysis (time point zero; “start")). The remaining solution was mixed with 6 µL of 200 mM HEPES pH 7.5, 2 µL KCl solution (2 M), and finally with 2 µL $MgCl_2$ solution (40 mM) to reach a final concentration of 55 µM of each RNA strand, 30 mM HEPES, 100 mM KCl and 2 mM Mg^{2+}. The cleavage reaction was initiated by the addition of the $MgCl_2$ solution. Samples (3 x 2 µL each) were drawn after the indicated time points, quenched by the addition of equal amounts of 40 mM Na_2H_2EDTA, and diluted with H_2O (to a total volume of 100 µL). The ribozyme cleavage reaction was analyzed in triplicate. We assayed the ribozyme under single turnover conditions for reasons of comparison to our previous studies on the twister ribozyme.

2 Supporting Table

Supporting Table S1. Mass spectrometric analysis of unmodified und modified RNAs used in this study.

#	Sequence 5' – 3'	nt	Molecular weight Calc.	found
**I**	GGC AGA GGC	9	2932.87	2932.97
**Ia**	GGC AIA GGC	9	2917.85	2917.57
**Ib**	GGC Ac^{1}IA GGC	9	2916.87	2916.57
**II**	GGC UAG CC	8	2524.59	2524.40
**IIa**	GGC UAI CC	8	2509.57	2509.25
**IIb**	GGC UAc^{1}I CC	8	2508.58	2508.32
**III**	GGA CCG GUC C	10	3174.98	3174.48
**IIIa**	GGA CCG IUC C	10	3159.96	3159.96
**IIIb**	GGA CCG c^{1}IUC C	10	3158.97	3158.46
**IV**	GAA GGG CAA CCU UCG	15	4813.99	4813.80
**Iva**	GAA IGG CAA CCU UCG	15	4798.97	4798.83
**IVb**	GAA c^{1}IGG CAA CCU UCG	15	4797.98	4797.69
**V**	$I_{15}C_{15}$	30	9468.59	9468.43
**Va**	I_7C_7	14	4385.63	4385.28
**Vb**	$I_6c^1IC_7$	14	4384.63	4384.61

Supporting Table S2 Thermodynamic parameters of modified RNAs (and reference) under different ionic conditions obtained by UV melting analysis.[a]

#	RNA sequence	c_{NaCl} [mM]	T_m [°C]	ΔT_m [°C]
**IV**	5’ GAAGG-GCAA-CCUUCG	30	66.5	-
**IV**	5’ GAAGG-GCAA-CCUUCG	60	68.4	1.9
**IV**	5’ GAAGG-GCAA-CCUUCG	150	71.0	4.5
**IV**	5’ GAAGG-GCAA-CCUUCG	300	72.8	6.3
**IVa**	5’ GAAIG-GCAA-CCUUCG	30	52.7	-
**IVa**	5’ GAAIG-GCAA-CCUUCG	60	54.6	1.9
**IVa**	5’ GAAIG-GCAA-CCUUCG	150	57.4	4.7
**IVa**	5’ GAAIG-GCAA-CCUUCG	300	59.4	6.7
**IVb**	5’ GAAc^1IG-GCAA-CCUUCG	30	36.1	-
**IVb**	5’ GAAc^1IG-GCAA-CCUUCG	60	38.3	2.2
**IVb**	5’ GAAc^1IG-GCAA-CCUUCG	150	41.3	5.2
**IVb**	5’ GAAc^1IG-GCAA-CCUUCG	300	43.4	7.3

[a] Conditions: c(RNA) = 10 µM, 10 mM sodium phosphate buffer, pH = 7.0; NaCl concentrations as indicated.

Supporting Table S3. Statistics of the NMR solution structure of the GAAIG-GCAA-CCUUCG **IVa** containing an inosine-cytidine base pair (I4-C11).

	**GAAIG-GCAA-CCUUCG**
**PDB ID**	30SC
Total numbers of restrains	342
NOEs	199
-Intra-residue	140
-Inter-residue	59
Torsion angles	91
Planarity	15
H-bonds	12
RDC	25
**NMR ensemble**	
RMSD NOE restraints (Å)	0.110 ± 0.001
NOE violation (>0.5 Å)	0
Torsion violations (>5°)	0
RMSD of the mean coordinates (Å)	0.511 ± 0.019
RDC violation	0.841 ± 0.091 Hz
**RMSD from ideal geometry**	
-bond lengths (Å)	0.004 ± 0.000
-bond angles (°)	0.678 ± 0.017
Total energy (kcal/mol)	-33.62 ± 7.65

3 Supporting Figures

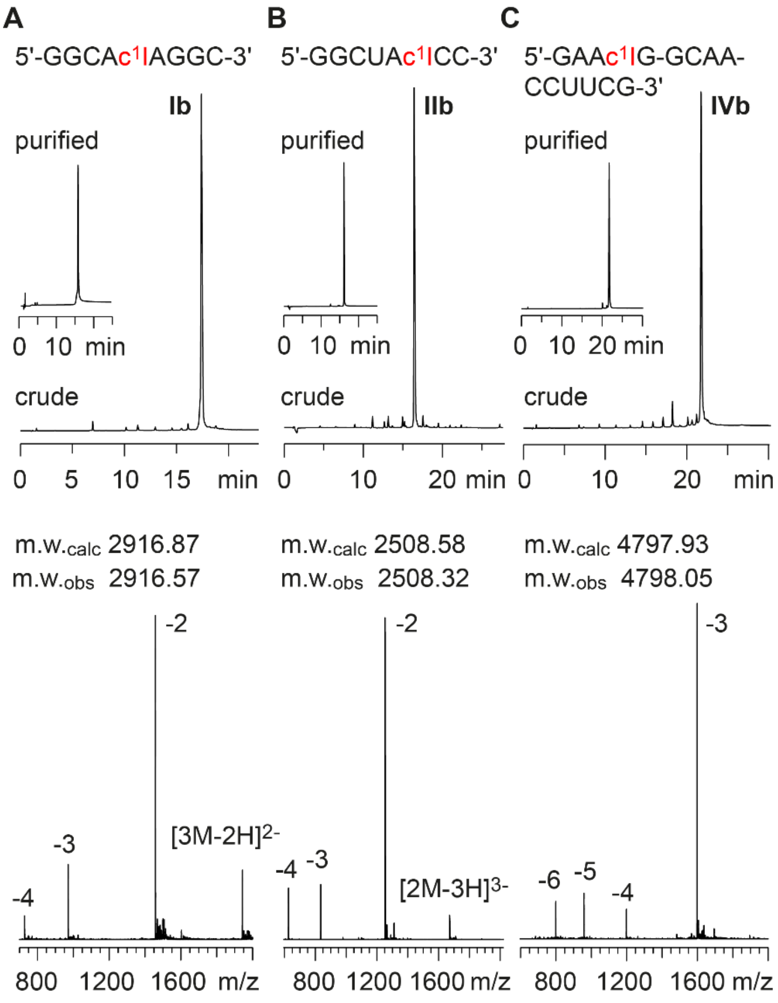

Supporting Figure S1. Characterization of c^1I-modified RNA synthesized by standard RNA solid-phase synthesis using c^1I building block **7**. Anion-exchange HPLC traces (top) of purified 9 nt RNA (**A**), 8 nt RNA (**B**), and 15 nt RNA (**C**), and corresponding LC-ESI mass spectra (bottom). HPLC conditions: Dionex DNAPac column (4 × 250 mm), 80 °C (or as indicated), 1 mL min^{-1}, 0−60% buffer B in 45 min; buffer A: Tris−HCl (25 mM), 10 mM $NaClO_4$, pH 8.0, 20% acetonitrile; buffer B: Tris−HCl (25 mM), 600 mM $NaClO_4$, pH 8.0, 20% acetonitrile. For LC-ESI MS conditions, see Supporting Methods, section 1.5.

A

II

5'-G C
G C
C G
U A
A U
G C
C G
C G

concentration / µM		T_m (duplex) / °C
1.92		55.4
5.24		58.2
13.2		60.5
38.9		63.2
101		66.3
ΔG^0 kcal mol^{-1}	ΔH^0 kcal mol^{-1}	ΔS^0 cal mol^{-1} K^{-1}
-15.4 ± 0.3	-81.7 ± 2.8	-223 ± 10

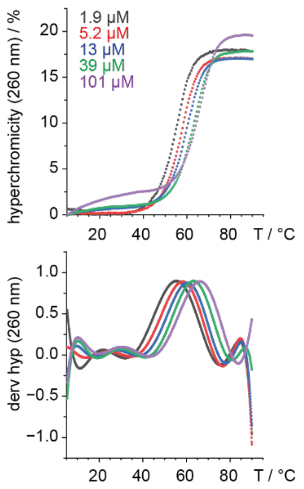

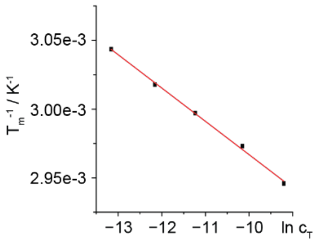

B

IIa

5'-G C
G C
C I
U A
A U
I C
C G
C G

concentration / µM		T_m (duplex) / °C
1.58		36.2
4.17		39.0
10.7		42.1
32.0		45.7
80.2		47.8
ΔG^0 kcal mol^{-1}	ΔH^0 kcal mol^{-1}	ΔS^0 cal mol^{-1} K^{-1}
-10.3 ± 0.1	-65.1 ± 3.4	-184 ± 11

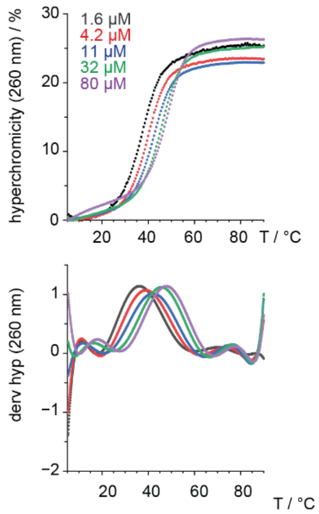

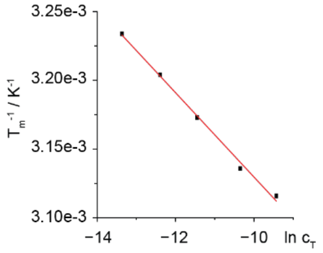

C

IIb

5'-G C
G C
C c¹I
U A
A U
c¹I C
C G
C G

concentration / µM		T_m (duplex) / °C
1.70		15.8
4.52		18.1
12.9		21.2
38.4		26.3
93.5		29.6
ΔG^0 kcal mol^{-1}	ΔH^0 kcal mol^{-1}	ΔS^0 cal mol^{-1} K^{-1}
-6.22 ± 0.6	-50.2 ± 8.4	-148 ± 26

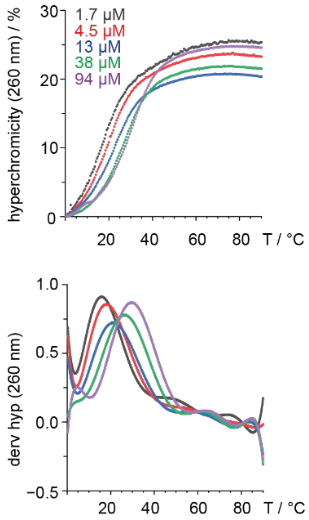

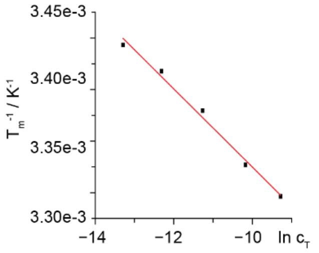

Supporting Figure S2. UV melting analysis of RNA base pairing. **A)** Palindromic 8 nt RNA duplex **II**: Sequence, secondary structure, and summary of RNA concentrations, T_m values and thermodynamic values with error limits derived from the deviation of three independent measurements for a confidence interval of 95%. Graph illustrating the superposition of UV-melting profiles (middle top), superposition of the first derivatives (middle bottom), and ln c versus 1/T plot (right); bimolecular concentration dependent melting transitions, assuming a two-state melting process for evaluating the van't Hoff parameters. **B)** Same as (A), but for the inosine modified RNA **IIa**. **C)** Same as (A), but for the c¹I modified RNA **IIb**. Note that the strand register is dominantly shifted to a 4 bp duplex with 3' overhangs **IIb'** (instead of 8 bp duplex **II**; see main text). Conditions: 10 mM Na_2HPO_4, 150 mM NaCl, pH 7.0.

A

III

5' G = C
G = C
A – U
C   G
C = G
G = C
G   C
U – A
C = G
C = G

concentration / µM	T_m (duplex) / °C
2.05	70.7
5.67	72.5
13.6	74.1
38.8	77.6
106	82.3

ΔG^0 kcal mol^{-1}	ΔH^0 kcal mol^{-1}	ΔS^0 cal mol^{-1} K^{-1}
-18.7 ± 0.9	-83.9 ± 6.3	-219 ± 18

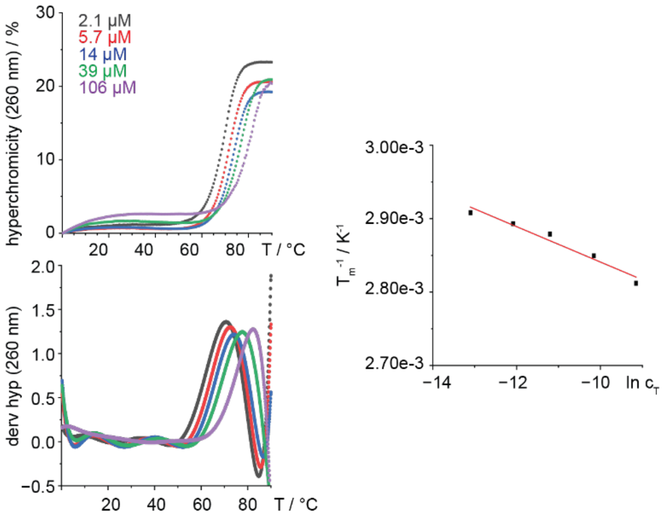

B

IIIa

5' G = C
G = C
A – U
C   I
C = G
G = C
I   C
U – A
C = G
C = G

concentration / µM	T_m (duplex) / °C
1.87	56.0
5.62	58.1
14.5	59.8
106	68.3

ΔG^0 kcal mol^{-1}	ΔH^0 kcal mol^{-1}	ΔS^0 cal mol^{-1} K^{-1}
-14.4 ± 0.1	-72.7 ± 0.3	-195 ± 1

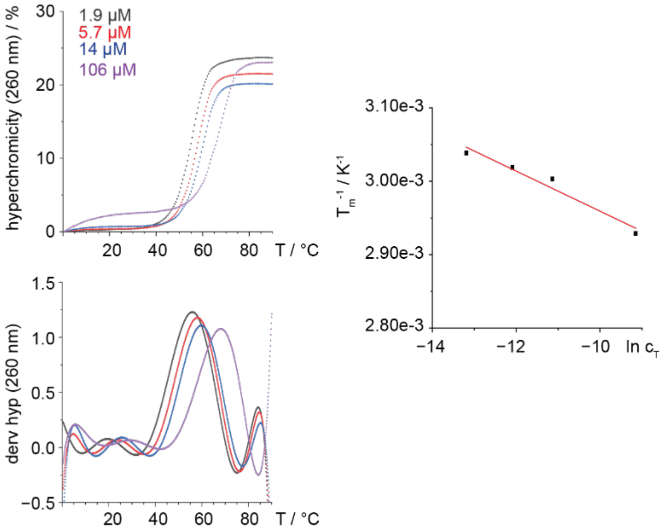

C

IIIb

5' G = C
G = C
A – U
C   c^{1}I
C = G
G = C
c^{1}I   C
U – A
C = G
C = G

concentration / µM	T_m (duplex) / °C
1.58	37.8
5.00	40.6
13.3	42.6
44.3	44.0
92.5	47.1

ΔG^0 kcal mol^{-1}	ΔH^0 kcal mol^{-1}	ΔS^0 cal mol^{-1} K^{-1}
-11.8 ± 0.9	-93.4 ± 7.2	-274 ± 21

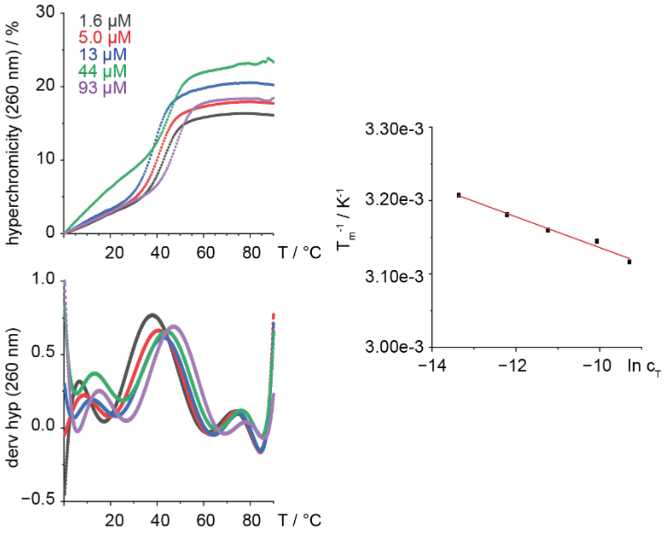

Supporting Figure S3. UV Melting Analysis of RNA base pairing. **A)** Palindromic 10 nt RNA duplex **III**: Sequence, secondary structure, and summary of RNA concentrations, T_m values and thermodynamic values with error limits derived from the deviation of three independent measurements for a confidence interval of 95%. Graph illustrating the superposition of UV-melting profiles (middle top), superposition of the first derivatives (middle bottom), and ln c versus 1/T plot (right); bimolecular concentration dependent melting transitions, assuming a two-state melting process for evaluating the van't Hoff parameters. **B)** Same as (A), but for inosine modified RNA **IIIa**. **C)** Same as (A), but for the c^{1}I modified RNA **IIIb**. Conditions: 10 mM Na_2HPO_4, 150 mM NaCl, pH 7.0.

A

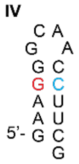

concentration / µM	T_m (hairpin) / °C	
2.2	71.2	
8.9	71.2	
ΔG^0 kcal mol^{-1}	ΔH^0 kcal mol^{-1}	ΔS^0 cal mol^{-1} K^{-1}
-7.53 ± 0.2	-57.9 ± 1.8	-169 ± 5

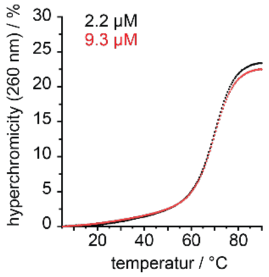

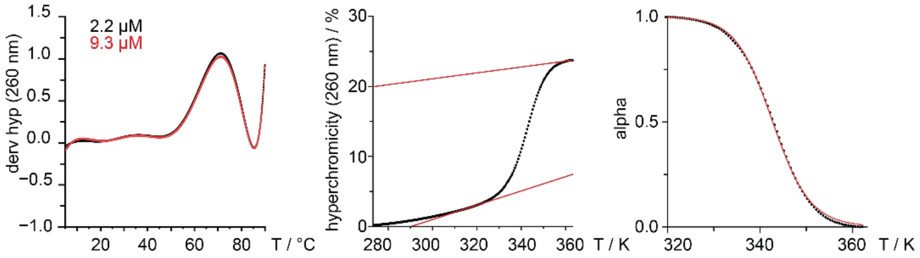

B

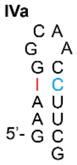

concentration / µM	T_m (hairpin) / °C	
2.2	57.7	
9.3	57.4	
ΔG^0 kcal mol^{-1}	ΔH^0 kcal mol^{-1}	ΔS^0 cal mol^{-1} K^{-1}
-4.87 ± 1.3	-50.7 ± 0.9	-154 ± 3

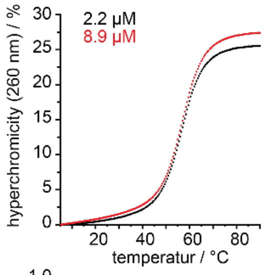

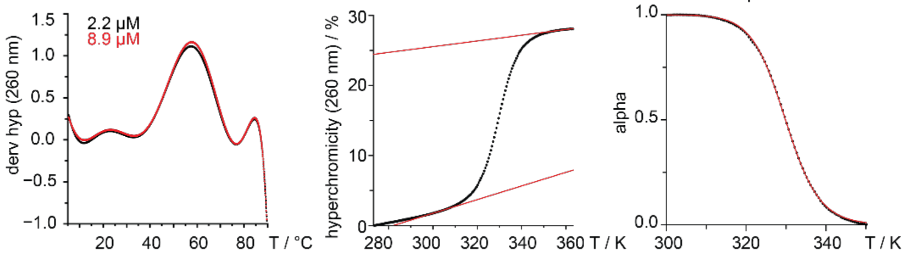

C

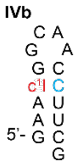

concentration / µM	T_m (hairpin) / °C	
2.1	41.5	
9.0	41.5	
ΔG^0 kcal mol^{-1}	ΔH^0 kcal mol^{-1}	ΔS^0 cal mol^{-1} K^{-1}
-2.68 ± 0.6	-41.8 ± 8.8	-131 ± 29

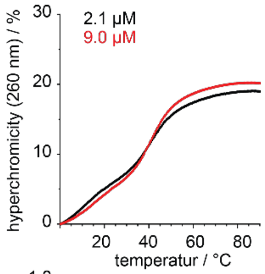

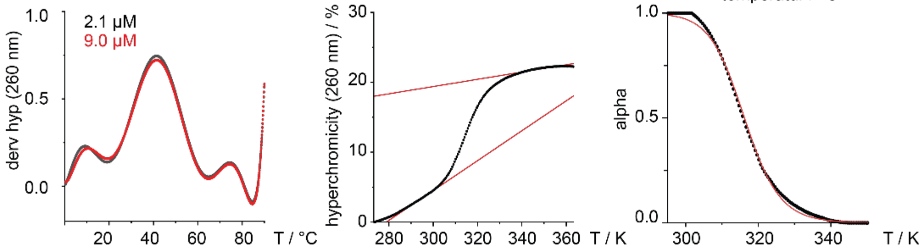

Supporting Figure S4. UV melting analysis of RNA base pairing. **A)** 15 nt RNA hairpin **IV**: Sequence, secondary structure, summary of RNA concentrations, T_m values and thermodynamic values with error limits derived from the deviation of three independent measurements for a confidence interval of 95%. Graph illustrating the superposition of UV-melting profiles (middle top), superposition of the first derivatives (bottom left), and graphical analysis of melting curves (right), unimolecular concentration independent melting transition, assuming a two-state melting process for evaluating the van't Hoff parameters. **B)** Same as (A), but for the RNA **IVa**. **C)** Same as (A), but for the RNA **IVb**. Conditions: 10 mM Na_2HPO_4, 150 mM NaCl, pH 7.0.

A

Va

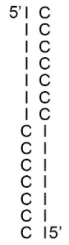

concentration / µM	T$_m$ (duplex) / °C
1.6	24.5
4.1	26.7
10.2	28.4
29.1	30.3
79.6	32.7

ΔG^0 kcal mol^{-1}	ΔH^0 kcal mol^{-1}	ΔS^0 cal mol^{-1} K^{-1}
-7.71 ± 0.2	-88.4 ± 2.3	-271 ± 7

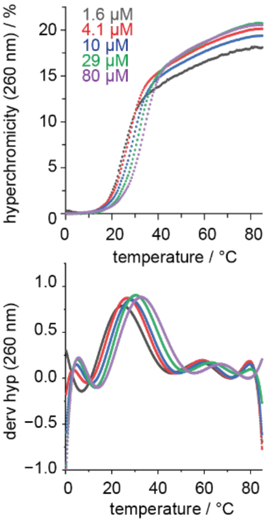

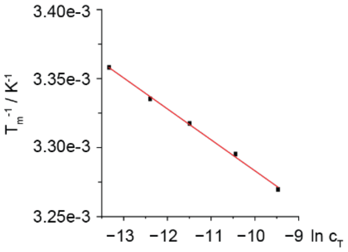

B

Vb

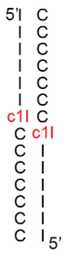

concentration / µM	T$_m$ (duplex) / °C
1.9	4.79
4.6	6.66
11.9	9.29
35.2	13.3
89.1	15.3

ΔG^0 kcal mol^{-1}	ΔH^0 kcal mol^{-1}	ΔS^0 cal mol^{-1} K^{-1}
-3.71 ± 0.3	-56.8 ± 4.6	-178 ± 15

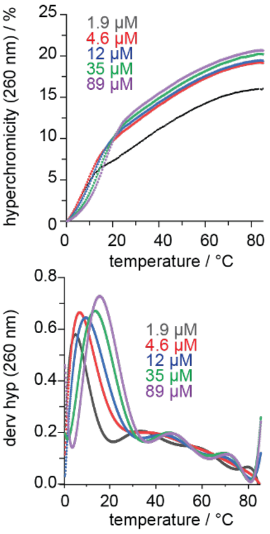

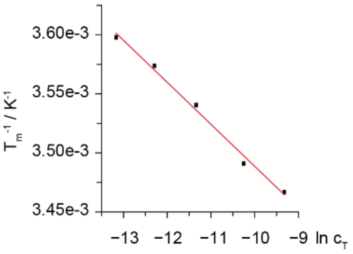

C

V

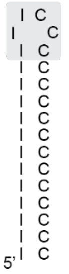

concentration / µM	T$_m$ (hairpin) / °C
2.2	50.2
8.3	50.3

ΔG^0 kcal mol^{-1}	ΔH^0 kcal mol^{-1}	ΔS^0 cal mol^{-1} K^{-1}
-5.15 ± 0.4	-57.3 ± 3.3	-174 ± 10

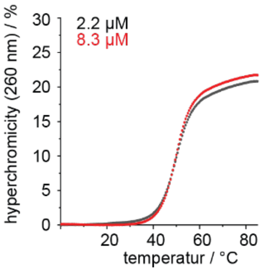

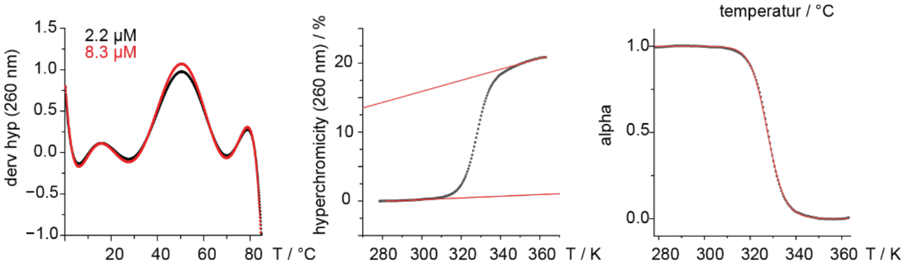

Supporting Figure S5. UV melting analysis of RNA base pairing. **A)** 14 nt poly I:C RNA **Va**: Sequence, secondary structure, summary of RNA concentrations, T$_m$ values and thermodynamic values with error limits derived from the deviation of three independent measurements for a confidence interval of 95%. Graph illustrating the superposition of UV-melting profiles (middle top), superposition of the first derivatives (middle bottom) and ln c versus 1/T plot (right; bimolecular concentration dependent melting transitions, assuming a two-state melting process for evaluating the van't Hoff parameters). **B)** Same as (A) but for the RNA **Vb**. **C)** Same as (A) but for the RNA **V**; graphical analysis of melting curves (right); unimolecular concentration independent melting transition, assuming a two-state melting process for evaluating the van't Hoff parameters. Conditions: 10 mM Na_2HPO_4, 150 mM NaCl, pH 7.0.

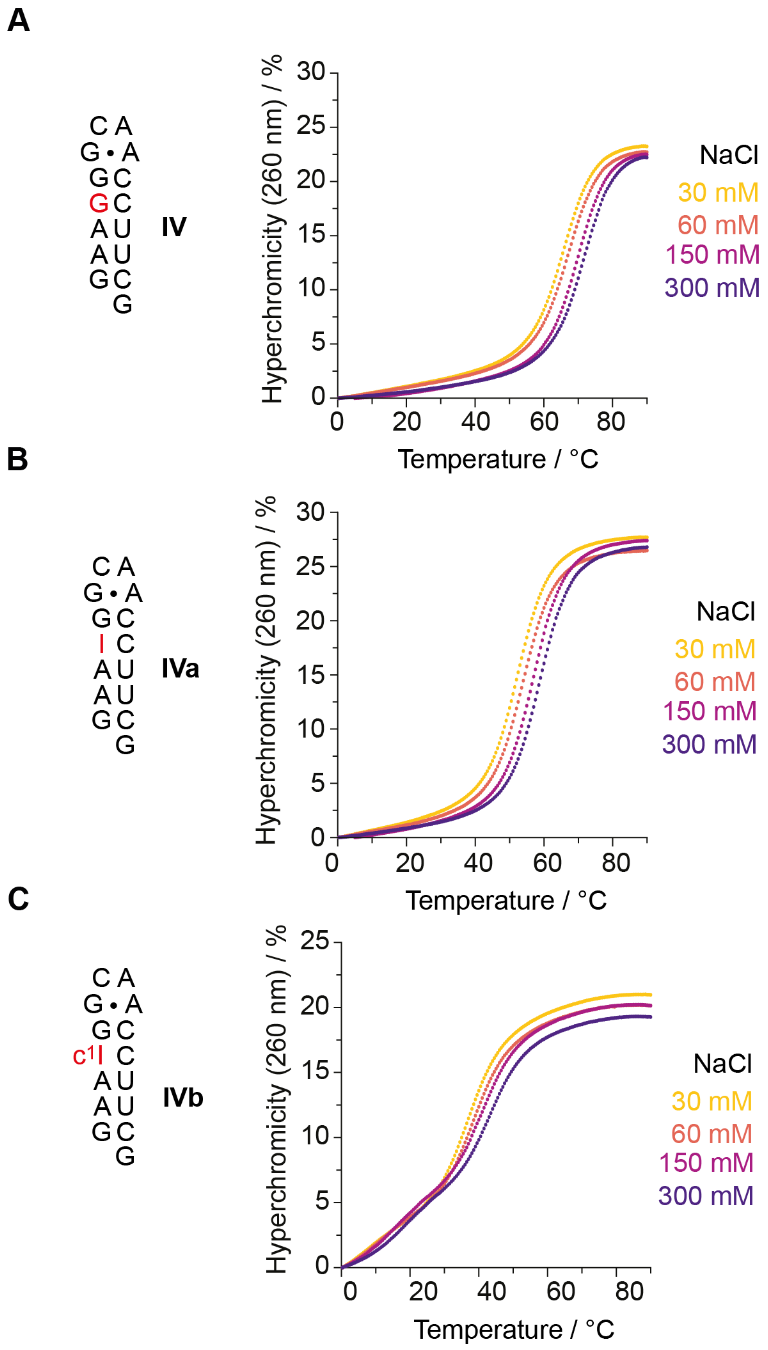

Supporting Figure S6. UV melting analysis of RNA base pairing – Salt dependence. **A)** 15 nt RNA hairpin **IV**: Sequence, secondary structure, and overlay of melting profiles at different NaCl concentrations. **B)** Same as (A), but for the RNA **IVa**. **C)** Same as (A), but for the RNA **IVb**. For hairpin **IVb** a second melting transition at low temperature is emerging which seem dependent on the NaCl concentration. This transition may account for the competing formation of a mismatched duplex. For melting temperatures see Supporting Table S2 Conditions: 10 mM Na_2HPO_4, pH 7.0, NaCl concentrations as indicated.

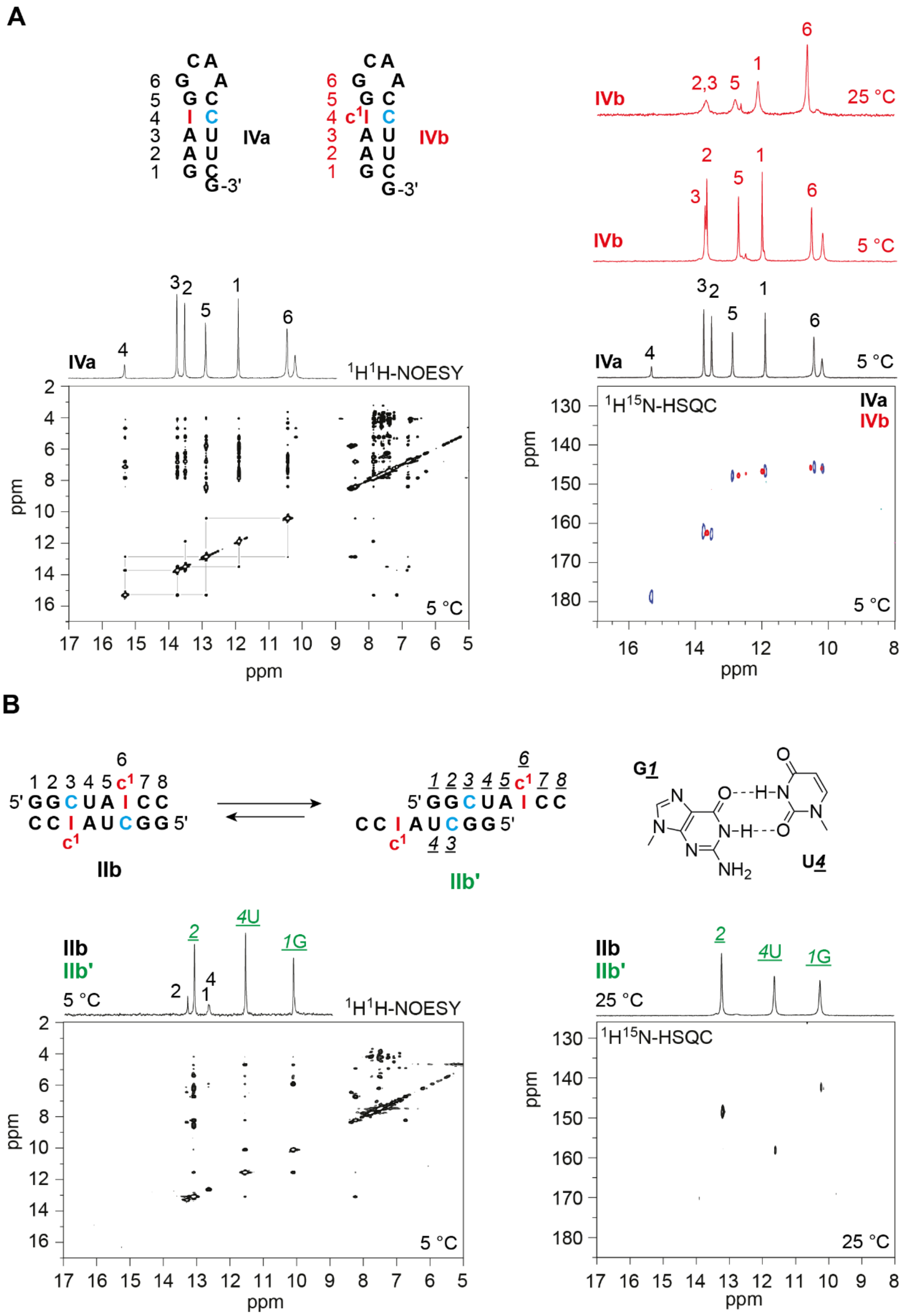

Supporting Figure S7. NMR spectroscopy of I and c^{1}I modified RNA. **A)** Sequences, secondary structures, and NMR spectra of inosine (**IVa**) and 1-deazainosine (**IVb**) modified 15 nt hairpins. ^{1}H,^{1}H NOESY spectrum (bottom left) shows the correlation of the imino protons and their assignment in the ^{1}H spectrum. An overlay of the ^{1}H,^{15}N HSQC spectra (bottom right) of **IVa** and **IVb** is shown. The correlation between imino protons and the corresponding nitrogen atoms is shown (typical ^{15}N chemical shifts: inosine N1 resonance at 178 ppm, uridine N3 resonances around 160 ppm, and guanine N1 resonances around 145 ppm. **B)** The palindrome GGCUAc^{1}ICC prefers a 4-base pair (**IIb'**) over an 8-base pair (**IIb'**) register in a temperature dependent equilibrium. ^{1}H^{1}H NOESY spectrum (bottom left) shows the strong correlation between G1 and 4U which is typical for a G-U mismatch [4]. ^{1}H^{15}N HSQC spectrum (bottom right), note that at 25°C, **IIb'** is the dominantly formed with hardly any **IIb** pairing register observed. Conditions: c(RNA) = 0.5 mM,15 mM NaH_2PO_4, 25 mM NaCl, 10% D_2O, pH 6.5, temperature as indicated.

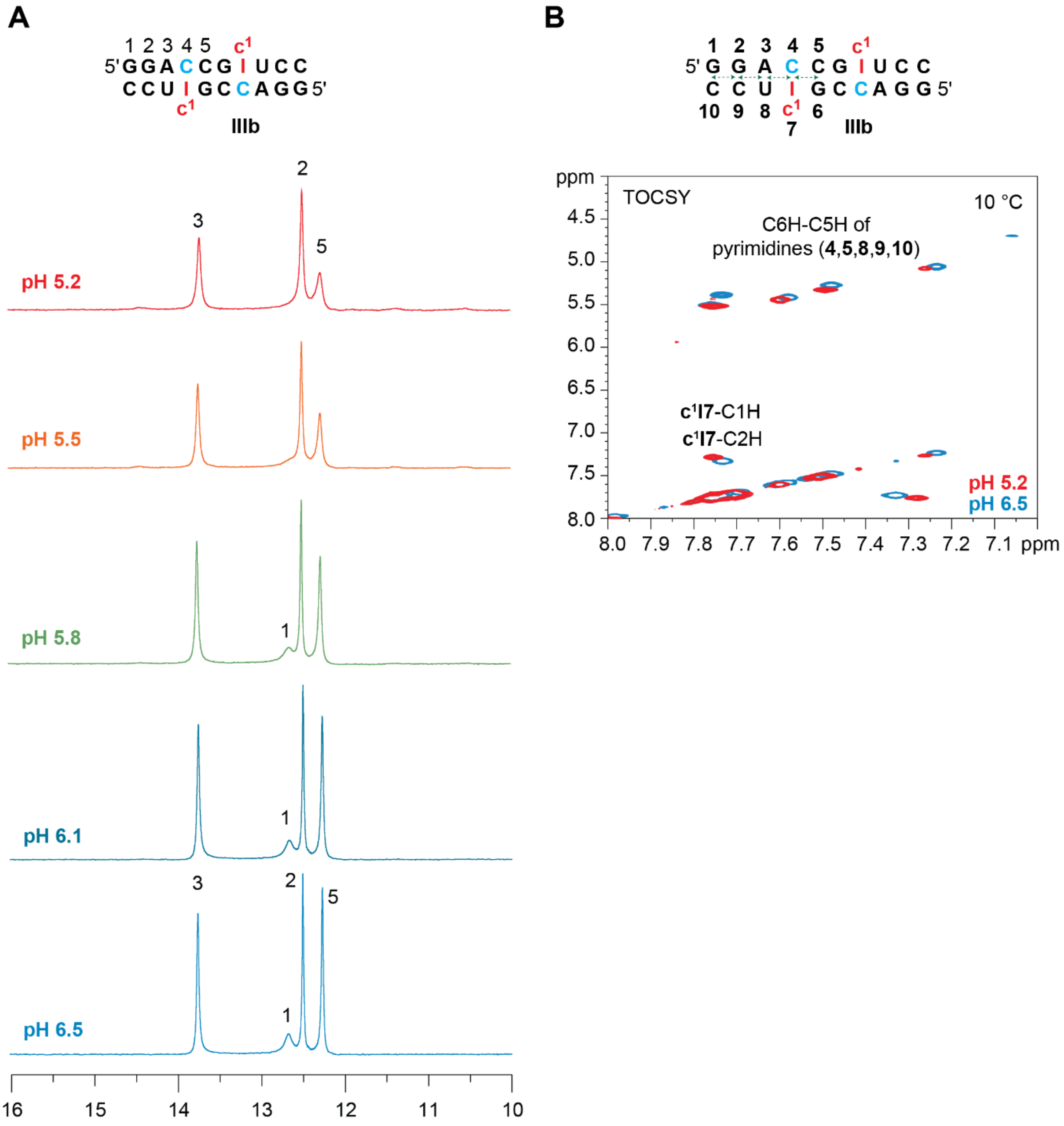

Supporting Figure S8. ^{1}H-NMR measurements at varying pH levels of a c^{1}I modified RNA. **A)** For the palindromic duplex GGACCGc^{1}IUCC **IIIb**, the four imino proton signals (^{1}H-NMR) broadened at lowering the pH (6.5, 6.1, 5.8, 5.5, and 5.2), and the signal of the terminal G-C base pair disappeared (pH 5.5, 5.2), most likely due to proton exchange with the solvent. **B**) Overlay of ^{1}H,^{1}H-TOCSY spectra of RNA **IIIb** at two different pH levels. The correlation peak between c^{1}I7-C1H and c^{1}I7-C2H shifted similarly to the correlation peaks between the C6H and cC5H atoms of pyrimidines. Therefore, we found no indication that the c^{1}I-C pairing mode would change within the tested pH range. Conditions: c(RNA) = 0.4 mM, 25 mM NaCl, 15 mM sodium phosphate buffer, H_2O/D_2O 9/1, pH values as indicated.

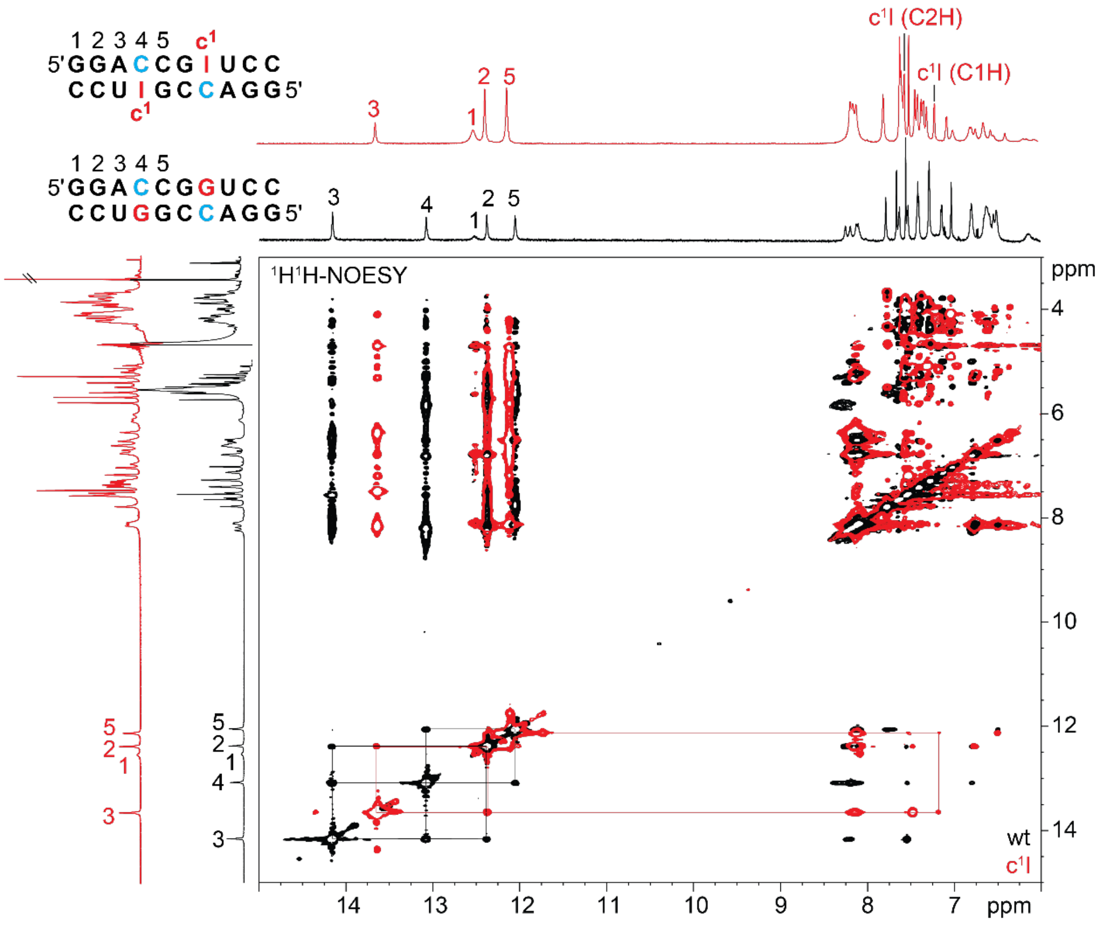

Supporting Figure S9. Overlay of the ^{1}H,^{1}H-NOESY spectrum of the unmodified RNA **III** and c^{1}I modified RNA **IIIb**. Conditions: c(RNA) = 0.5 mM,15 mM NaH_2PO_4, 25 mM NaCl, 10% D_2O, pH 6.5 at 5°C.

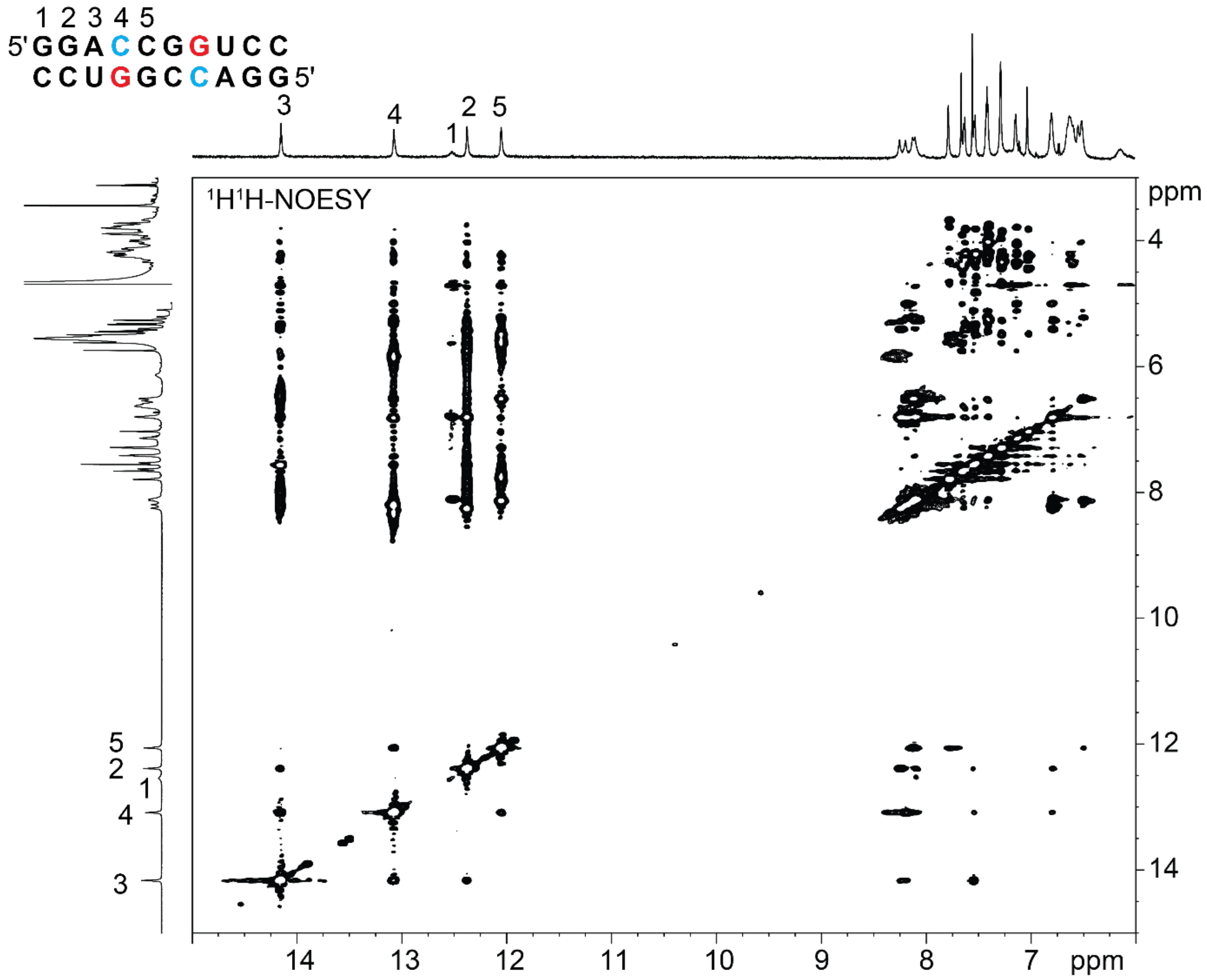

Supporting Figure S10. ^{1}H,^{1}H-NOESY spectrum of the unmodified RNA **III**. Conditions: c(RNA) = 0.5 mM, 15 mM NaH_2PO_4, 25 mM NaCl, 10% D_2O, pH 6.5 at 5°C.

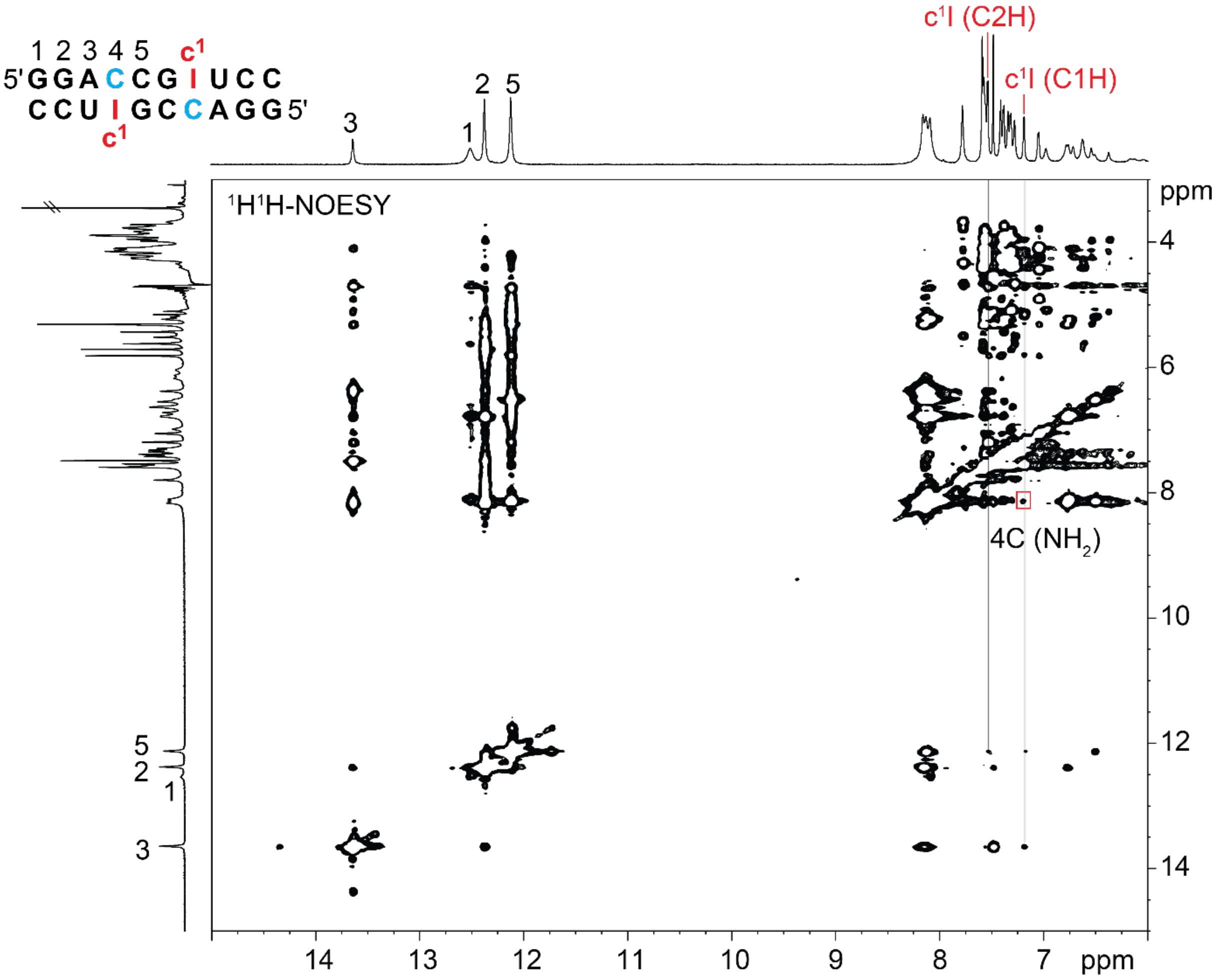

Supporting Figure S11. $^{1}H,^{1}H$-NOESY spectrum of the c1I modified RNA **IIIb**. Conditions: c(RNA) = 0.5 mM, 15 mM NaH_2PO_4, 25 mM NaCl, 10% D_2O, pH 6.5 at 5°C.

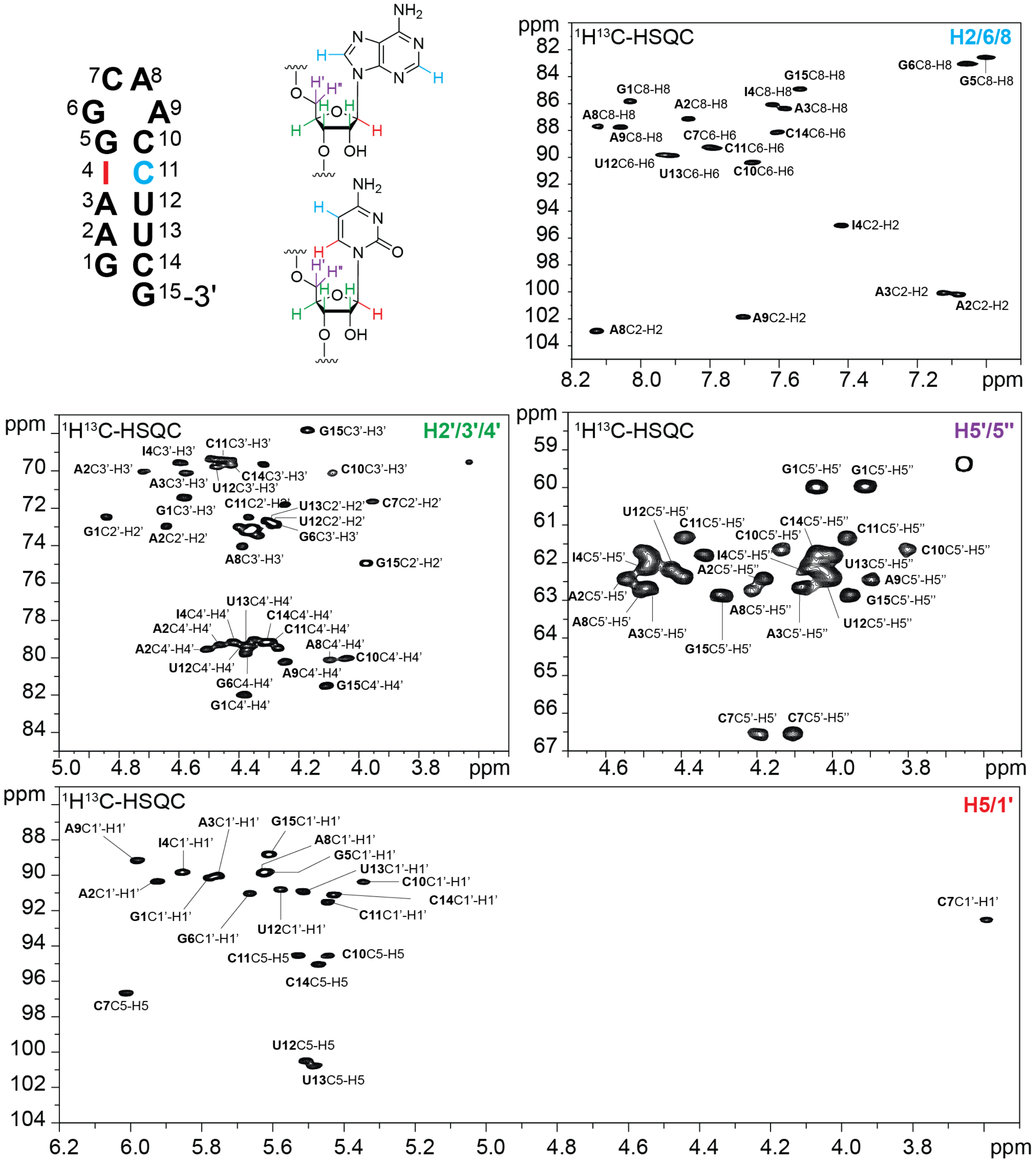

Supporting Figure S12. $^1H^{13}C$-HSQC spectra of the 15 nt hairpin GAAIG-GCAA-CCUUCG (**IVa**) highlighting characteristic resonance clusters used for proton assignment. Shown are selected spectral regions corresponding to aromatic H2/6/8 protons, ribose H2'/H3'/H4', H5'/H5'', and H6/H1' correlations, annotated according to proton type. The distinct clustering of sugar and base resonances provides the initial framework for resonance classification and serves as the starting point for the combined HSQC-NOESY assignment strategy. Conditions: c(RNA) = 0.5 mM, 15 mM NaH_2PO_4, 25 mM NaCl, 10% D_2O, pH 6.5 at 5°C

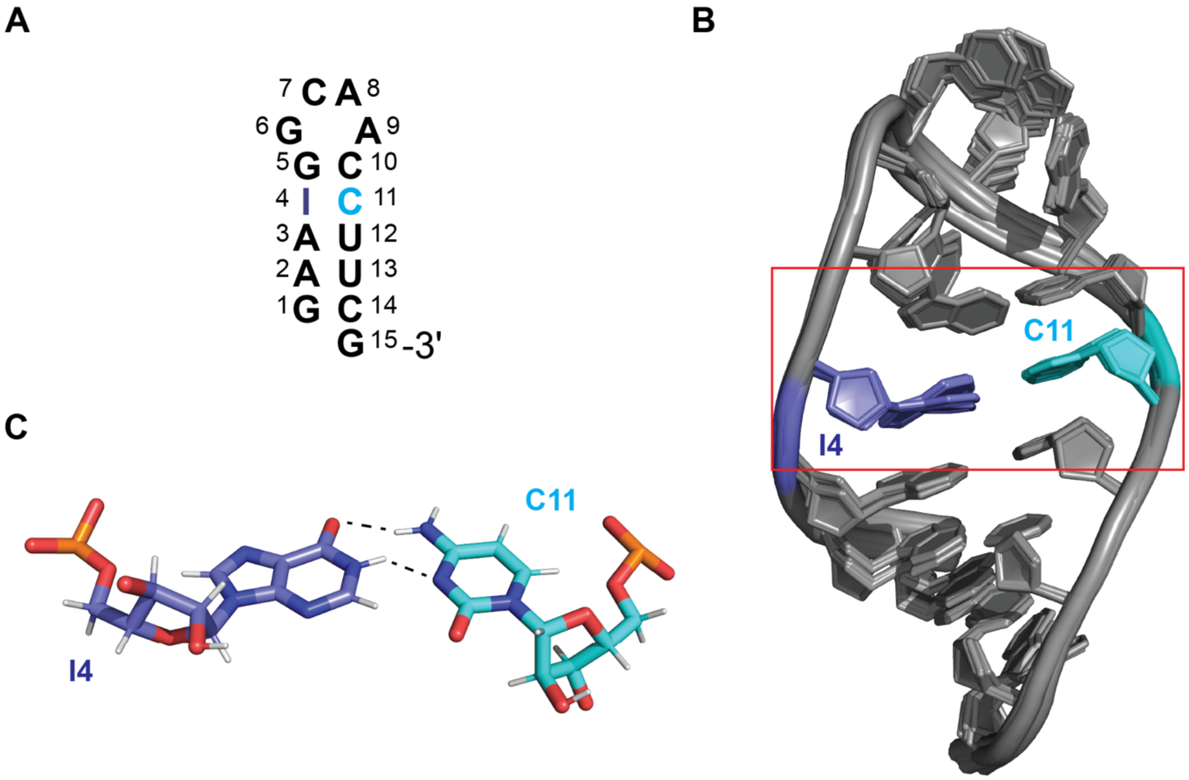

Supporting Figure S13. Solution structure of a 15 nt hairpin containing an inosine-cytidine base-pair. (**A**) RNA sequence and numbering, GAAIG-GCAA-CCUUCG (**IVa**). (**B**) Overlay of the NMR-derived structural ensemble of the 15 nt hairpin **IVa**, with the I-C pair highlighted (red box) (PDB ID SC30). (**C**) Expanded view of the I-C base pair.

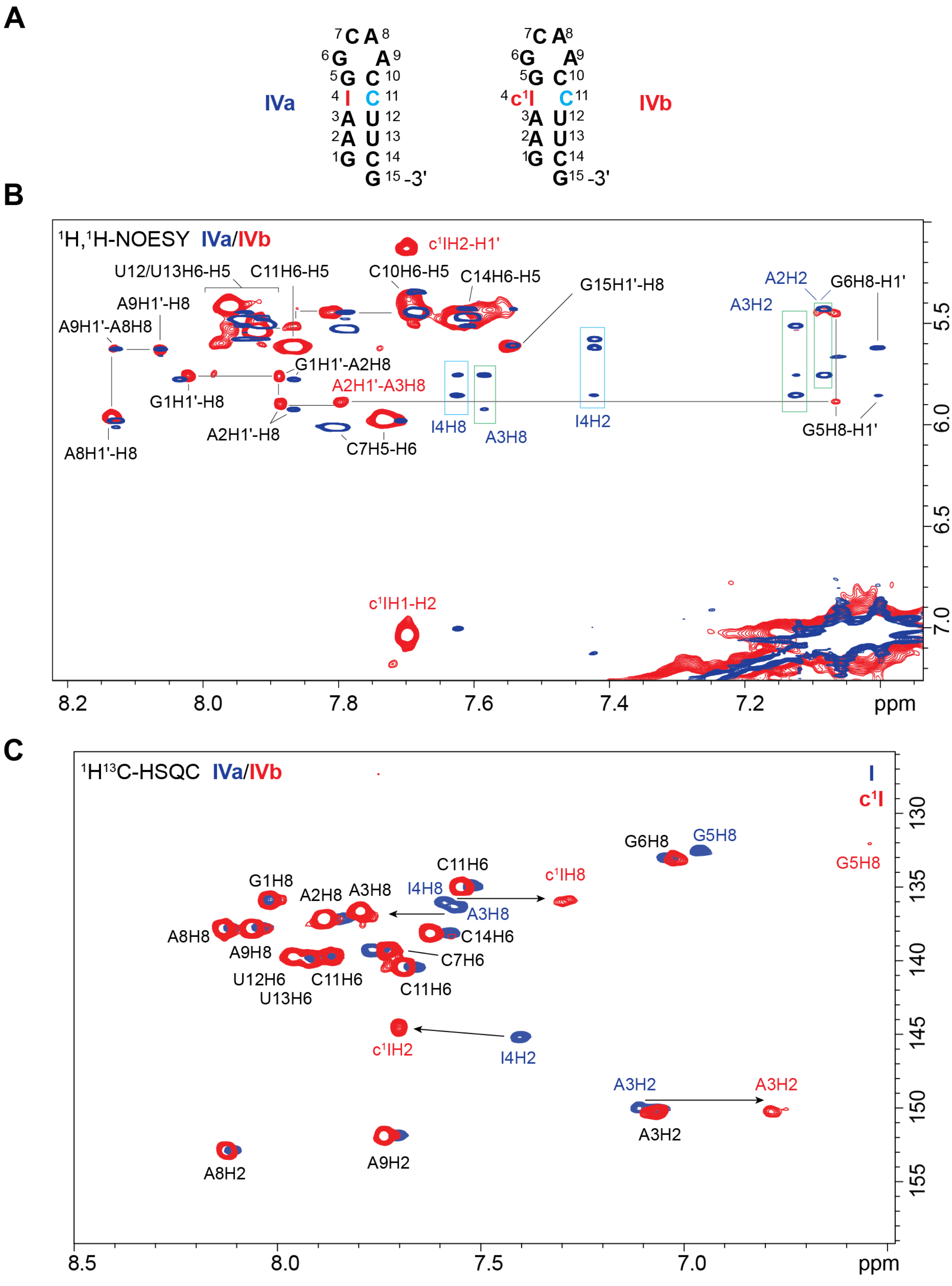

Supporting Figure S14. NMR spectroscopic comparison of the 15 nt hairpins GAAIG-GCAA-CCUUCG (**IVa**) versus GAAc^{1}IG-GCAA-CCUUCG (**IVb**). (**A**) Overlay of ^{1}H,^{1}H-NOESY spectra of inosine RNA **IVa** (blue) and 1-deazainosine RNA **IVb** (red). (**B**) Overlay of ^{1}H,^{13}C-HSQC NMR spectra of inosine RNA **IVa** (blue) and 1-deaza-inosine RNA **IVb** (red). Assigned resonances are labelled, and selected diagnostic cross-peaks are highlighted by rectangles. The close agreement of the NOE pattern and the overall similarity of the ^{1}H-^{13}C correlations indicate that both modified oligonucleotides adopt the same hairpin fold. The replacement of inosine (I4) with 1-deazainosine (c^{1}I4) results in modest spectral differences of the neighbouring base pairs (G5–C10; A3–U12) only, indicated that they remain conformationally largely unaffected. Conditions: c(RNA) = 0.5 mM, 15 mM NaH_2PO_4, 25 mM NaCl, 10% D_2O, pH 6.5 at 5°C

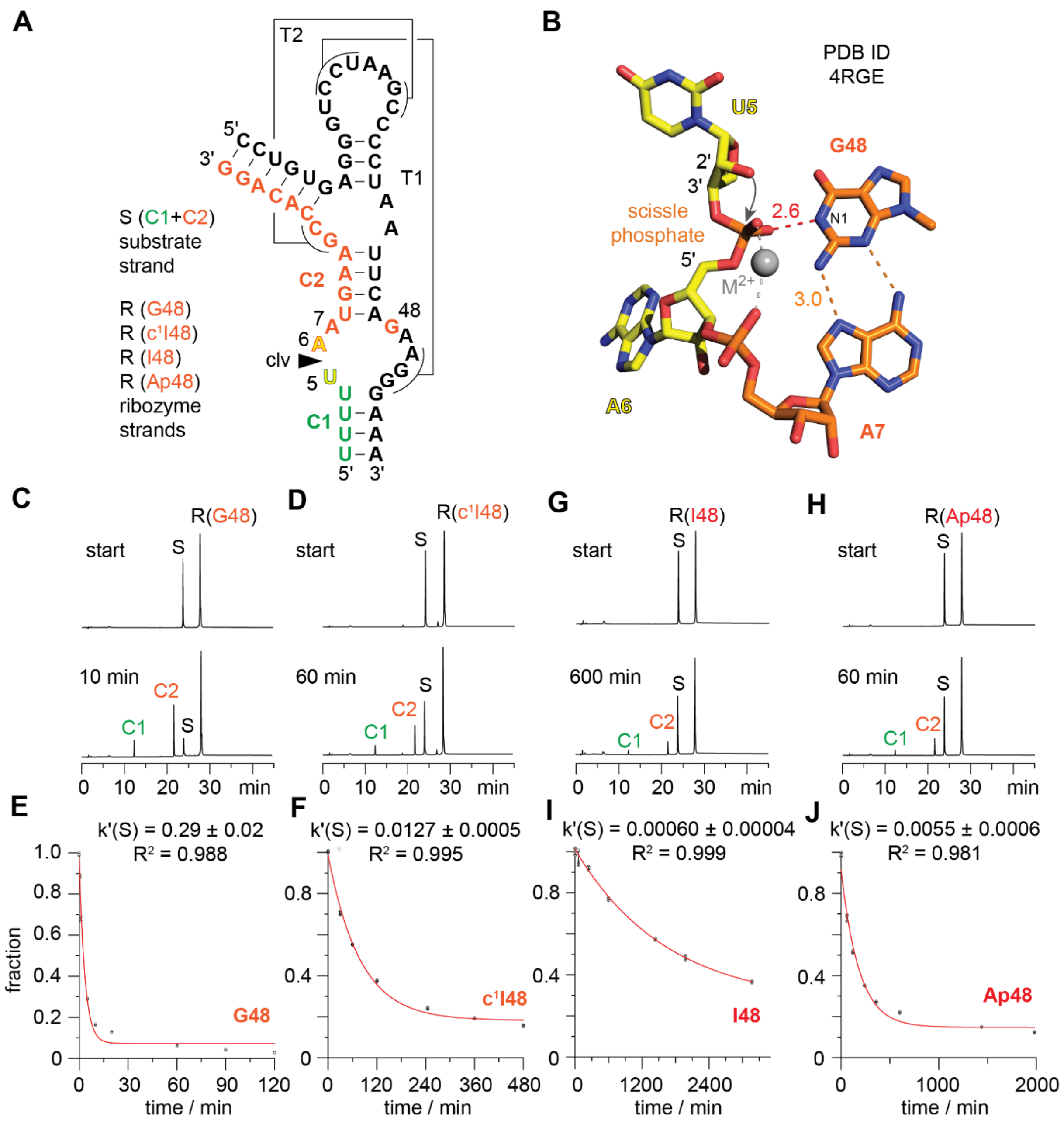

Supporting Figure S15. Extended Figure 8: Atomic mutagenesis of the twister ribozyme: impact of an active site G-to-c^{1}I mutation on activity to elucidate the mechanism of the phosphodiester cleavage. (**A**) Secondary structure of the two-strand ribozyme assembly used for functional cleavage assays. (**B**) X-ray structure of the twister ribozyme (precatalytic state; PDB ID 4RGE); interactions of guanine-48 with the scissile phosphate and A7 is shown; the 2′-OH nucleophile is modeled on dU5; distances in Å. (**C, D**) HPLC traces of wild-type G48 and c^{1}I48 modified ribozymes at two time points each illustrate that product formation of the c^{1}I-modified ribozyme is impeded under otherwise same reaction conditions. (**E, F**) Cleavage rate determination of wild-type G48 and c^{1}I48 ribozymes. The latter is 23-fold slower. (**G, H**) HPLC traces of inosine (I48) and 2-aminopurine (Ap48) modified ribozymes at two time points each illustrate that product formation of the I-modified ribozyme is impeded as well compared to wt (G48) ribozyme, under otherwise same reaction conditions. (**I, J**) Cleavage rate determination of I48 and Ap48 modified ribozymes. The I48 modified ribozyme is 480-fold slower, the Ap48 modified ribozyme is 53-fold slower, compared to the wt (G48) ribozyme.

4 References

1. R. Bereiter, E. Renard, K. Breuker, C. Kreutz, E. Ennifar, R. Micura, *J. Am. Chem. Soc.* **2022**, *144*, 10344–10352.
2. W. Lee, M. Rahimi, Y. Lee, A. Chiu, *Bioinformatics (Oxford, England)* **2021**, *37*, 3041–3042.
3. a) C. D. Schwieters, J. J. Kuszewski, N. Tjandra, G. M. Clore, *Journal of Magnetic Resonance* **2003**, *160*, 65–73; b) C. Schwieters, J. Kuszewksi, G. Mariusclore, *Progress in Nuclear Magnetic Resonance Spectroscopy* **2006**, *48*, 47–62;
4. B. Fürtig, C. Richter, J. Wöhnert, H. Schwalbe, *ChemBioChem* **2003**, *4*, 936–962.
